# Exploring the Effect of Halogenation in a Series of Potent and Selective A_2B_ Adenosine Receptor Antagonists

**DOI:** 10.1021/acs.jmedchem.2c01768

**Published:** 2022-12-14

**Authors:** Rubén Prieto-Díaz, Manuel González-Gómez, Hugo Fojo-Carballo, Jhonny Azuaje, Abdelaziz El Maatougui, Maria Majellaro, María I. Loza, José Brea, Víctor Fernández-Dueñas, M. Rita Paleo, Alejandro Díaz-Holguín, Beatriz Garcia-Pinel, Ana Mallo-Abreu, Juan C. Estévez, Antonio Andújar-Arias, Xerardo García-Mera, Iria Gomez-Tourino, Francisco Ciruela, Cristian O. Salas, Hugo Gutiérrez-de-Terán, Eddy Sotelo

**Affiliations:** † Center for Research in Biological Chemistry and Molecular Materials (CIQUS), University of Santiago de Compostela, 15782Santiago de Compostela, Spain; ‡ Department of Organic Chemistry, Faculty of Pharmacy, University of Santiago de Compostela, 15782Santiago de Compostela, Spain; § Department of Cell and Molecular Biology, Uppsala University, Biomedical Center, 75124Uppsala, Sweden; ∥ Center for Research in Molecular Medicine and Chronic Diseases (CiMUS), University of Santiago de Compostela, 15782Santiago de Compostela, Spain; ⊥ Department of Pharmacology, Pharmacy and Pharmaceutical Technology, Faculty of Pharmacy, University of Santiago de Compostela, 15782Santiago de Compostela, Spain; # Department of Biochemistry and Molecular Biology, Faculty of Pharmacy, University of Santiago de Compostela, 15782Santiago de Compostela, Spain; ∇ Pharmacology Unit, Department of Pathology and Experimental Therapeutics, Faculty of Medicine and Health Sciences, Institute of Neuroscience, University of Barcelona, 08907L’Hospitalet de Llobregat, Spain; ○ Neuropharmacology and Pain Group, Neuroscience Program, Institut d’Investigació Biomèdica de Bellvitge, IDIBELL, 08907L’Hospitalet de Llobregat, Spain; ◆ Department of Organic Chemistry, Faculty of Chemistry and Pharmacy, Pontificia Universidad Católica de Chile, Vicuña Mackenna 4860, Macul, Santiago7820436, Chile

## Abstract

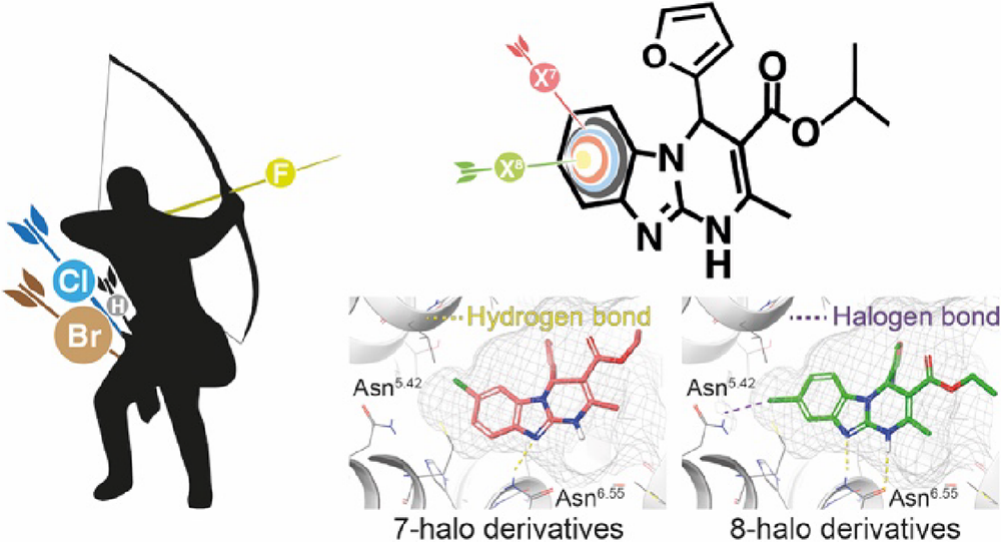

The modulation of the A_2B_ adenosine receptor is a promising strategy in cancer (immuno) therapy, with A_2B_AR antagonists emerging as immune checkpoint inhibitors. Herein, we report a systematic assessment of the impact of (di- and mono-)halogenation at positions 7 and/or 8 on both A_2B_AR affinity and pharmacokinetic properties of a collection of A_2B_AR antagonists and its study with structure-based free energy perturbation simulations. Monohalogenation at position 8 produced potent A_2B_AR ligands irrespective of the nature of the halogen. In contrast, halogenation at position 7 and dihalogenation produced a halogen-size-dependent decay in affinity. Eight novel A_2B_AR ligands exhibited remarkable affinity (*K*_*i*_ < 10 nM), exquisite subtype selectivity, and enantioselective recognition, with some eutomers eliciting sub-nanomolar affinity. The pharmacokinetic profile of representative derivatives showed enhanced solubility and microsomal stability. Finally, two compounds showed the capacity of reversing the antiproliferative effect of adenosine in activated primary human peripheral blood mononuclear cells.

## Introduction

Adenosine (Ado) is an essential signaling nucleoside that is either released from cells or extracellularly generated, by sequential hydrolysis of adenosine 5′-triphosphate and adenosine 5′-monophosphate by the ectonucleotidases CD39 and CD73.^1^ Ado is ubiquitous in mammalian cells, being a key intermediate metabolite that modulates several biochemical processes,^2,3^ ranging from energy transfer, signal transduction, sleep–wake cycle,^4^ inflammation,^5^ to immunity.^6,7^ In healthy tissues, the extracellular concentration of Ado is generally low, although it can increase rapidly from nanomolar to micromolar,^1^ which contributes to the protection of tissues against damage caused by stress, injury, hypoxia, or inflammation.^3,8,9^ Four rhodopsin-like G protein-coupled receptors (GPCRs) sense the extracellular Ado levels, constituting the class 1 purinergic receptors (P_1_) A_1_AR, A_2A_AR, A_2B_AR, and A_3_AR. Each adenosine receptor (AR) subtype has unique sequence homology, tissue distribution, second messenger coupling, and pharmacology.^8^ The physiology of Ado signaling through these receptors and its pathophysiological implications in human diseases have been recently reviewed.^1^

A_2B_AR remains the most puzzling and poorest-characterized AR subtype.^10^ Ubiquitously expressed but usually at low levels (except at human cecum, large intestine, mast cells, and hematopoietic cells), A_2B_AR displays the lowest affinity for Ado among all four subtypes (30–300 nM), remaining silent in healthy conditions.^1^ Such a behavior, together with its high structural homology with the well-characterized A_2A_AR, led to the initial perception that A_2B_AR would have only minor physiological relevance. However, it is now well documented that A_2B_AR becomes activated under particular pathological conditions such as hypoxia, inflammation, infection, and cancer, where extracellular Ado levels are increased up to micromolar concentrations, consequently increasing attention to the therapeutic potential of A_2B_AR as a drug target.^11^ A_2B_AR antagonists have been recently proposed as potential drugs for the treatment of inflammation, diabetes, pain, asthma, and Alzheimer disease.^12−15^ More recent is the identification of A_2B_AR as an important player to different facets of cancer progression, e.g., tumor growth, metastasis, and angiogenesis.^16,17^ Advances in cancer immunotherapy highlight the importance of Ado as a key metabolite that suppresses anti-tumor immune response by T and NK cells in the tumor microenvironment,^18^ this immune role being specifically mediated by A_2A_ and A_2B_ ARs.^19−21^ Indeed, several A_2A_AR antagonists are under clinical trials as immune checkpoint inhibitors for the treatment of different cancer types.^22^ A_2A_AR and A_2B_AR exhibit high homology and are often co-expressed on cells.^23^ Recent studies suggested formation of heteromeric A_2A_AR–A_2B_AR complexes and revealed that ligand recognition, signaling, and pharmacology of A_2A_AR are blocked by A_2B_AR.^24^ According to all this evidence, A_2B_AR antagonists and dual A_2A_AR-A_2B_AR antagonists are emerging as effective cancer (immuno)therapeutics, where the consequent reactivation of the immune system results in antiproliferative, antiangiogenic, and antimetastatic effects.^25,26^

**Figure 1:**
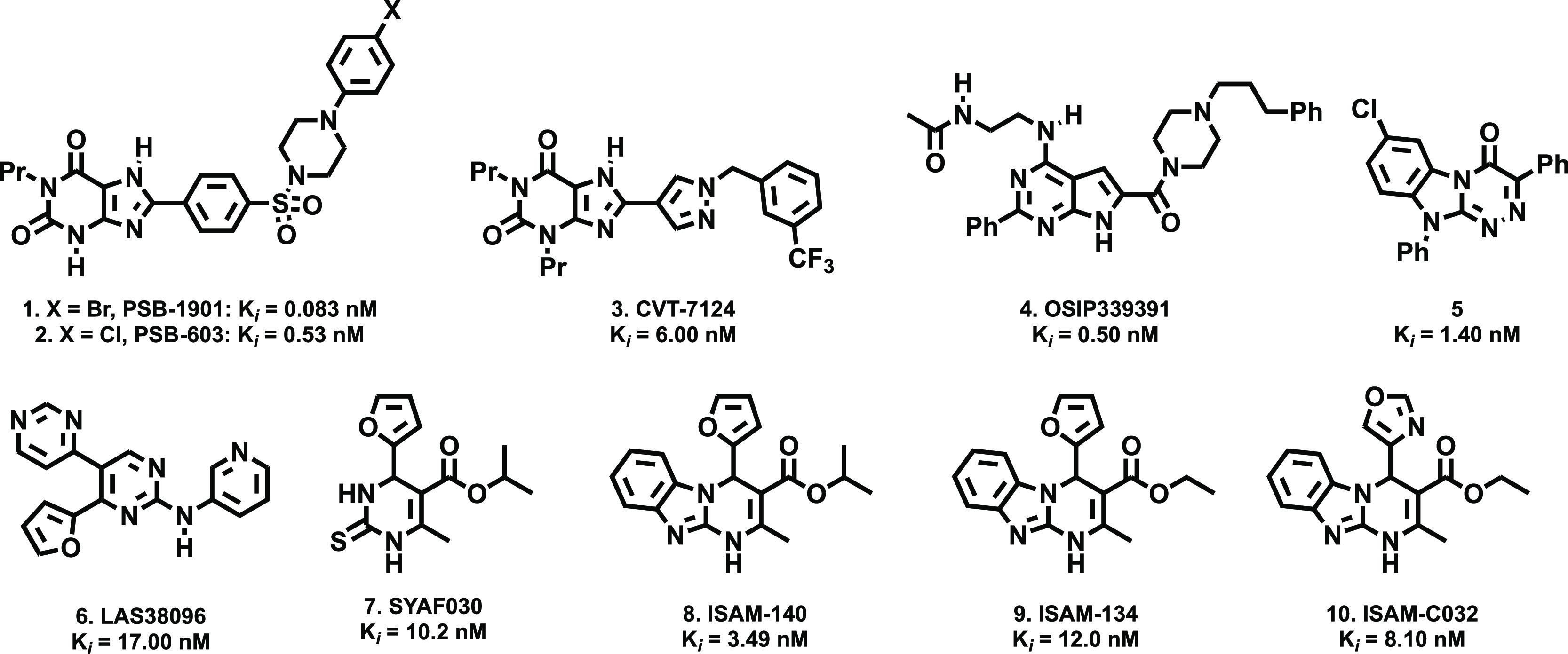
Structure of representative potent and selective A_2B_ antagonists.

The therapeutic opportunities arising from A_2B_AR modulation have driven the development of A_2B_AR antagonists. The naturally occurring xanthine derivatives (e.g., caffeine and theophylline) early inspired the discovery and optimization of xanthine congeners and structurally related deaza analogues and purine derivatives.^27^ Xanthines have been intensively studied, allowing the identification of derivatives eliciting optimal affinity–selectivity profiles (Figure 1, Cmpds **1**–**4**), being so far the most widely explored A_2B_AR antagonists.^28^ However, their challenging physicochemical features and pharmacokinetic (PK) profiles remain as the major drawback of this chemotype to advance further on the drug discovery pipeline. Consequently, recent efforts have been focused on the development of non-xanthinic A_2B_AR antagonists (Figure 1, Cmpds **5**–**10**).^29−32^ In this context, we have reported novel series of pyrimidine-based ligands (Figure 1, Cmpds **7**–**10**), which encompass structural novelty, exquisite affinity and selectivity, and excellent synthetic feasibility.^29−31^ A hybrid approach, combining scaffold hopping and thorough SAR investigation, have guided the expansion of these series. Importantly, these compounds contain a chiral center within the heterocyclic core (Figure 1, Cmpds **7**–**10**), offering a novel structural element as compared to classical, planar A_2B_AR antagonists. Consequent racemate separation provided the first examples of antagonists with enantiospecific A_2B_AR recognition.^25,31,33−35^

In the frame of an optimization program of non-xanthine A_2B_AR antagonists as promising cancer (immuno)therapeutics,^25,36^ we determined to analyze the impact of di- and mono-halogenation at positions 7 and 8 of the tricyclic core of prototypical A_2B_AR antagonists (Figure 1, Cmpds **8**–**10**).^30,31^ The main goal was to improve the pharmacodynamic and PK profiles of this chemotype, while maintaining (or even improving) the affinity and selectivity profiles. The phenyl ring of the tricyclic system is the most easily metabolizable part, only next to the exocyclic pentagonal ring that was already optimized in a previous work,^31^ and halogenation arises as a strategy to reduce the probability of intensive metabolization. At the same time, the size variability of halogen substitution would allow a deeper exploration of the interactions proposed by earlier modeling studies in the internal cavity of A_2B_AR.^29−31^ The synthesis and pharmacological characterization of a collection of 80 novel tricyclic ligands is here assessed by free energy perturbation (FEP) simulations, evidencing the superior profile of 8-halo derivatives by optimizing interactions with the modeled binding site. This study further confirms the previously modeled enantiospecific A_2B_AR recognition of the pentagonal ring at position 4, and the antagonist profile of the most promising compounds. In addition, examination of the ADMET profile of representative antagonists confirmed that the introduction of a fluorine atom improves solubility and microsomal stability. Two of the ligands here optimized were selected to investigate the effect of A_2B_AR antagonism on immune cell proliferation, demonstrating that the ligands reverse the reduction of Ado-related proliferation in human primary immune cells.

## Results and Discussion

### Design

These novel series were conceived in the context of our ongoing projects to develop multi-target cancer (immuno)therapeutics and PET tracers for A_2B_AR, starting from the scaffold of ISAM-140 (Figure 1) as a prototype ligand. Introduction of functional groups at positions 7 and 8 of the heterotricyclic core evidenced conspicuous effects on the affinity and selectivity profile, thus inspiring the design of the present series. It was anticipated that halogenation at positions 7 and 8 of the phenyl ring within the tricyclic core would not only result in compounds with improved pharmacodynamic and PK profiles but also provide valuable data to understand the molecular basis behind the observed SAR trends.

**Figure 2:**
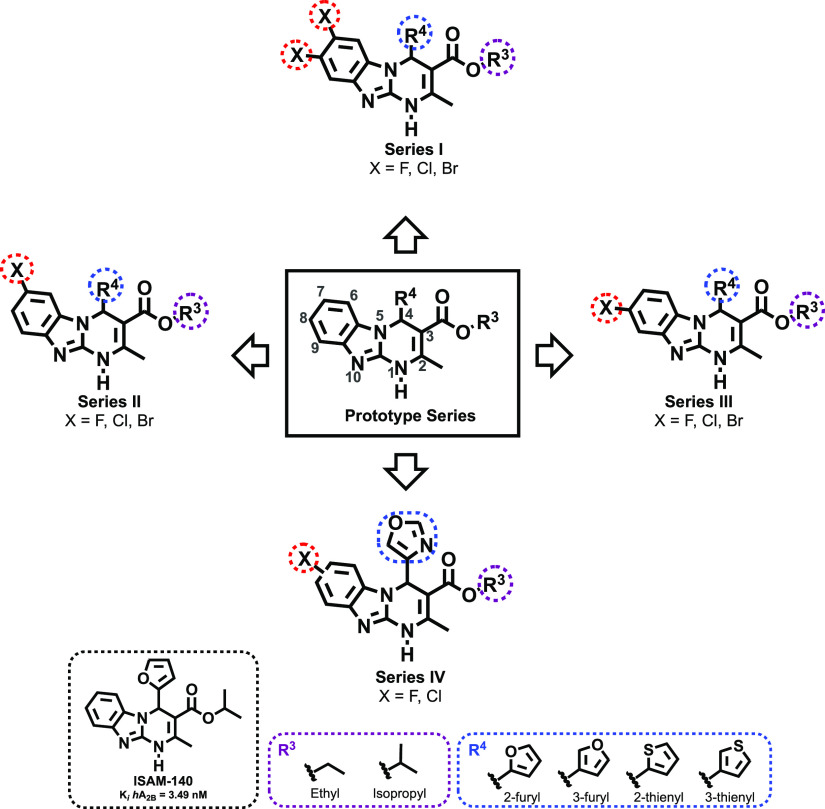
Design strategy and diversity elements explored during the study.

Thus, we envisioned the development of the 80 new ligands (series **I**–**IV**) presented in Figure 2. Being aware that the impact of halogenation on binding affinity and selectivity might be position-dependent and that it can heavily rely on the nature of the halogen, we systematically introduced F, Cl, and Br atoms at positions 7 and 8 of the tricyclic scaffold (Figure 2). To preserve consistency from the early series and facilitate the comparative SAR, the herein obtained ligands (series **I–IV**) retained the structural elements that provided optimal A_2B_AR affinity at positions 3 and 4 (Figure 2). Thus, for R^4^, we initially considered a set of four pentagonal heteroaryl groups (2-furyl, 3-furyl, 2-thienyl, 3-thienyl), while the alkoxy residues of the ester moiety (R^3^) consist of either ethyl or isopropyl groups. Three series were subsequently conceived, each consisting of 24 derivatives (Figure 2 and Tables 2–4). Series **I** contain two identical halogen atoms at positions 7 and 8 (compounds **14a**–**x**), whereas in series **II** and **III** (mono-halogenated), the halogen atom is present in position 7 or 8 respectively (compounds **15a**–**x** and **16a**–**x**). Finally, a focused series consisted of eight 7-halo or 8-halo derivatives bearing a 4-oxazolyl group (Table 5), which we have recently identified as an optimal non-furan pentagonal nucleus for position 4.^31^ This limited series was obtained by prioritizing two halogens (F and Cl) and the substitution patterns that provided better affinity and selectivity profiles in series **I**–**III**.

### Chemistry

**Scheme 1:**
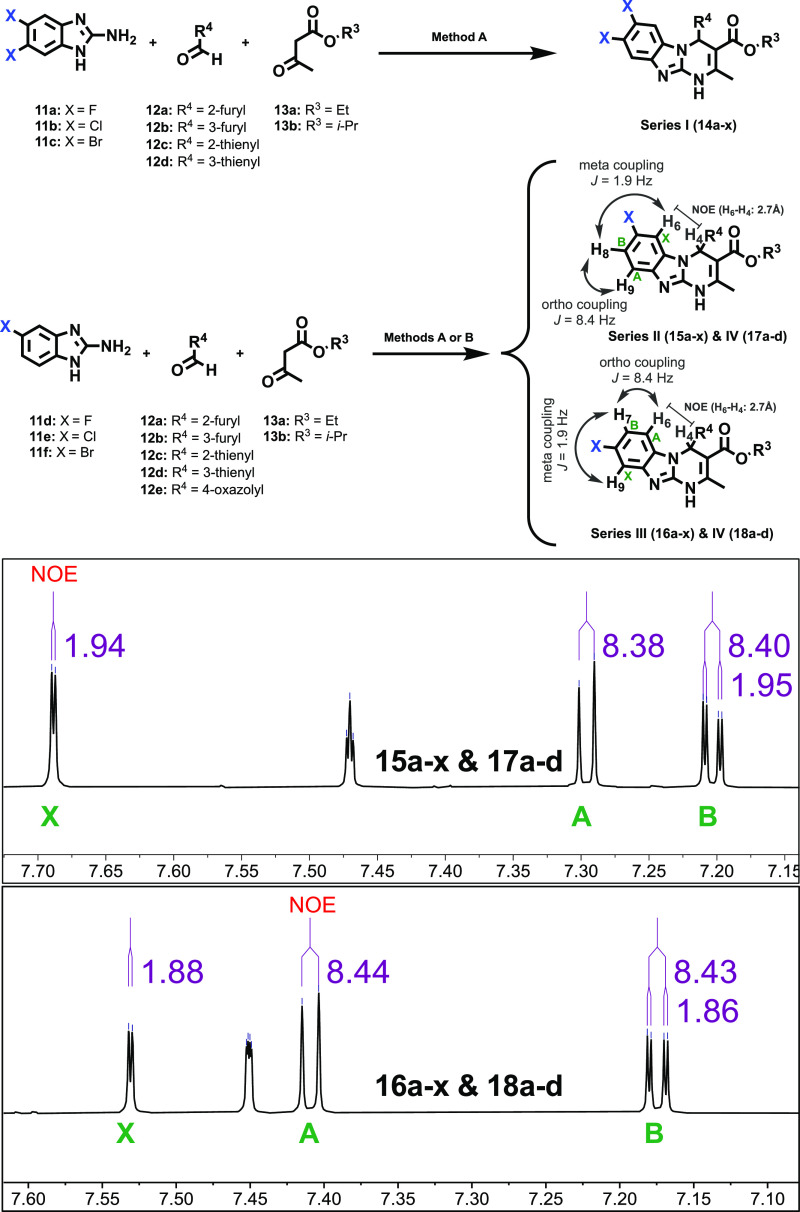
Biginelli-Based Synthesis. Method (A) ZnCl_2_, THF, 90 °C, 12 h. Method (B) AcOH, THF/DMF (2:1), 80 °C, MW, 90 min; and Regioisomer Characterization Strategy (in Red: Signal with NOE Effect Irradiating H_4_)

The halogenated alkyl 4-heteroaryl-2-methyl-1,4-dihydrobenzo[4,5]-imidazo[1,2-*a*]pyrimidine-3-carboxylates (**14**–**18**) were obtained as depicted in Scheme 1. A three-component synthesis, based on the robust and efficient Biginelli reaction (i.e., the three-component reaction of an aldehyde, a β-ketoester, and 1,3-dinucleophiles), enabled a time- and cost-effective assembly of the large collection obtained. A set of pentagonal carbaldehydes (**12a**–**e**) and β-ketoesters (**13a**,**b**) providing optimal substituents for positions 4 and 3 in combination with six halogenated 2-aminobenzimidazoles (**11a**–**f**) were employed as precursors. The starting materials, dissolved in THF and containing a catalytic amount of ZnCl_2_ (or acetic acid), were submitted to orbital stirring at 90 °C (12 h) or microwave radiation at 80 °C (90 min). The targeted tricyclic derivatives were obtained in moderate to satisfactory yields (30–85%). For the synthesis of a series of monohalogenated (7-halo or 8-halo) derivatives (Scheme 1, Cmpds **15**–**18**), the corresponding 2-amino-5-halobenzimidazoles (**11d**–**f**) were employed as precursors. The phenomenon of tautomerization enabled to directly obtain the targeted (7- or 8-)halogenated alkyl 4-heteroaryl-2-methyl-1,4-dihydrobenzo-[4,5]imidazo[1,2-*a*]pyrimidine-3-carboxylates as regioisomeric mixtures in a 1:1 ratio. Both regioisomers (series **II** and **III**) were isolated in pure forms after chromatographic separation. All reactions were monitored by thin-layer chromatography (TLC). After completion of the reaction, the solvent was evaporated to dryness and the isolated solid was purified by column chromatography on silica gel. A detailed description of the synthetic methods and the complete structural, spectroscopic, and analytical data for all compounds are provided in the experimental part. Unambiguous structural characterization of each regioisomer has been carried out with a combination of X-ray crystallography and 2D-NMR experiments (Scheme 1). The monohalogenated compounds (series **II**–**IV**) differ in the position of the halogen atom located at either C7 or C8. Both regioisomers present an ABX aromatic substitution pattern in the ^1^H NMR (Scheme 1). X-ray diffraction analysis of **16s** (see the Supporting Information) shows that protons H4 (alpha hydrogen to the five-membered ring) and H6 (aromatic proton) are 2.7 Å apart, allowing us to perform NOE experiments (Supplementary Figure S1). The 2D-ROESY NMR spectrum of **16s** (Supplementary Figure S1A) showed a correlation of H4 (singlet, δ 6.60 ppm) and the doublet (*J* = 8.4 Hz) at δ 7.41 ppm corresponding to H6. The coupling constant value is typical of H–H *ortho* coupling and indicates the presence of a hydrogen in C7 and therefore of the halogen at C8. However, the 2D-ROESY NMR spectrum of **15s** (Supplementary Figure S1B) showed a correlation between H4 (singlet at δ 6.53 ppm) and H6 (doublet at δ 7.70 ppm). The coupling constant value, *J* = 1.9 Hz, indicates a H–H *meta* coupling, and thus the halogen is in the *ortho* position (C7). Similar correlations were observed for other pairs of regioisomers (Supplementary Figure S2). As in previous series (Figure 1 and Table 1, compounds **7**–**10**), all ligands obtained in this study contain one stereocenter at position 4 of the heterocyclic core and were isolated and evaluated as racemic mixtures. Six ligands eliciting an attractive A_2B_AR affinity/selectivity profile were submitted to chiral resolution to isolate its corresponding enantiomer pairs.

### Biological Evaluation

The adenosinergic profile (affinity and selectivity) of the 80 novel halogenated alkyl 1,4-dihydro-benzo[4,5]imidazo[1,2-*a*]pyrimidine-3-carboxy-lates was evaluated in vitro using radioligand binding assays at the four human AR subtypes.^30,31,33,34^Tables 2–5 contain the binding data obtained, while, to facilitate the comparative assessment, Table 1 shows the binding data for the prototype series.^30^ All ligands reported in Tables 1–5 were obtained and tested as racemic mixtures. The whole set was evaluated in silico with a combination of a Rdkit^37^ PAINS filter and Instant JChem from Chemaxon (https://www.chemaxon.com), to rule out the possibility that these ligands can be promiscuous pan-assay interference compounds (PAINS) and to assess their bioavailability, respectively. Human ARs were expressed in transfected cell lines [e.g., Chinese hamster ovary (CHO) cells (A_1_AR), human epithelial carcinoma (HeLa) cells (A_2A_AR and A_3_AR), and human embryonic kidney (HEK-293) cells (A_2B_AR)]. [^3^H]DPCPX for A_1_AR and A_2B_AR, [^3^H]NECA for A_3_AR, and [^3^H]ZM241385 for A_2A_AR were employed as radioligands. The binding data is presented as *K*_*i*_ ± SEM (nM, *n* = 3) obtained by fitting the data by non-linear regression using Prism 5.0 software (GraphPad, San Diego, California), or as specific binding inhibition percentage at 1 μM (*n* = 2, average) for those compounds that did not completely displace the radioligand due to either little affinity or poor solubility. The binding affinity of reference AR ligands (ISAM-140, DPCPX, NECA, and ZM241385) was assessed using our experimental protocols and reported in Tables 1–5 for comparison. The stereoisomers of selected compounds were obtained by chiral resolution and tested at the four human AR subtypes in their enantiopure forms (Table 6). This data was employed to complement the SAR study and to evaluate the importance of the configuration of the stereogenic center on the affinity.

### Intrinsic Activity Assays

**Figure 3:**
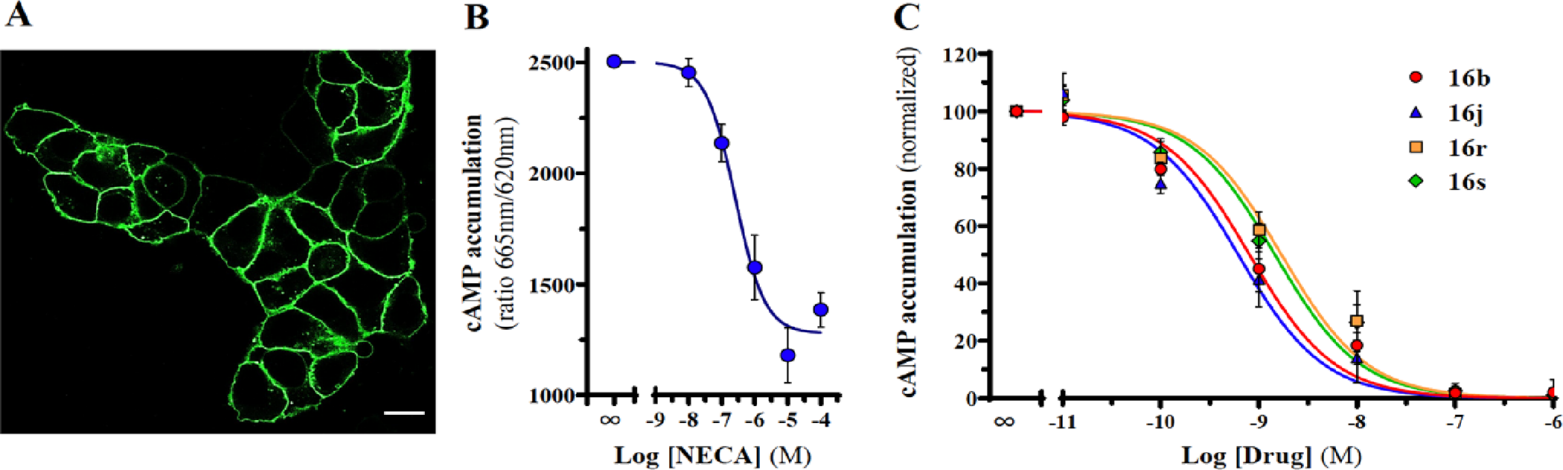
Blockade of A_2B_AR-mediated cAMP accumulation. (A) HEK-293 cells permanently expressing the A_2B_AR^SNAP^ construct were stained with the fluorescent SNAP-488 substrate and visualized using a confocal microscope.^38^ Scale bar: 10 μM. (B) Determination of NECA-mediated cAMP accumulation in A_2B_AR^SNAP^ cells. Cells were incubated in the absence or presence of increasing concentrations of NECA, and the cAMP accumulation determined as described in Experimental Section. The straight TR-FRET signal (ratio 665 nm/620 nm) of the assay is shown. (C) cAMP accumulation in A_2B_AR^SNAP^ cells stimulated with NECA (1 μM) in the absence or presence of increasing concentrations of **16b**, **16j**, **16r**, and **16s**. Data are expressed as mean ± SEM (*n* = 4).

Four representative ligands (**16b**, **16j**, **16r**, and **16s**) were selected to gain insight into the functional effect of the obtained series. To assess their ability to block A_2B_AR agonist intrinsic activity, the effect on A_2B_AR-mediated cAMP accumulation and cellular impedance was evaluated. To this end, we used HEK-293 cells permanently expressing the A_2B_AR^SNAP^ construct, which can be visualized upon staining the cells with a SNAP-fluorescent substrate. As shown in Figure 3A, A_2B_AR^SNAP^ nicely decorated the cell surface of A_2B_AR^SNAP^ cells, indicating that the receptor was readily expressed at the plasma membrane. Subsequently, we evaluated the effect of **16b**, **16j**, **16r**, and **16s** in A_2B_AR signaling by monitoring cAMP accumulation in A_2B_AR^SNAP^ cells treated with NECA, a non-selective Ado receptor agonist. NECA induced a concentration-dependent cAMP accumulation in A_2B_AR^SNAP^ cells with an EC_50_ of 257 ± 94 nM (Figure 3B). Subsequently, A_2B_AR^SNAP^ cells were evaluated with a fixed concentration of NECA (1 μM) in the absence or presence of increasing concentrations of **16b**, **16j**, **16r**, and **16s** and the *K*_B_ value for each compound was calculated, namely, 0.8 ± 0.2, 0.6 ± 0.2, 1.7 ± 0.6, and 1.5 ± 0.5 nM, respectively (Figure 3C). Overall, **16b**, **16j**, **16r**, and **16s** displayed low nanomolar potency while blocking A_2B_AR-mediated cAMP accumulation in A_2B_AR^SNAP^ cells, thus indicating an antagonist intrinsic activity nature. Comparative *K*_B_ and *K*_*i*_ data for selected compounds is shown in Table 7. It should be noticed that two these selective A_2B_AR antagonists exhibit sub-nanomolar *K*_B_ data.

**Figure 4:**
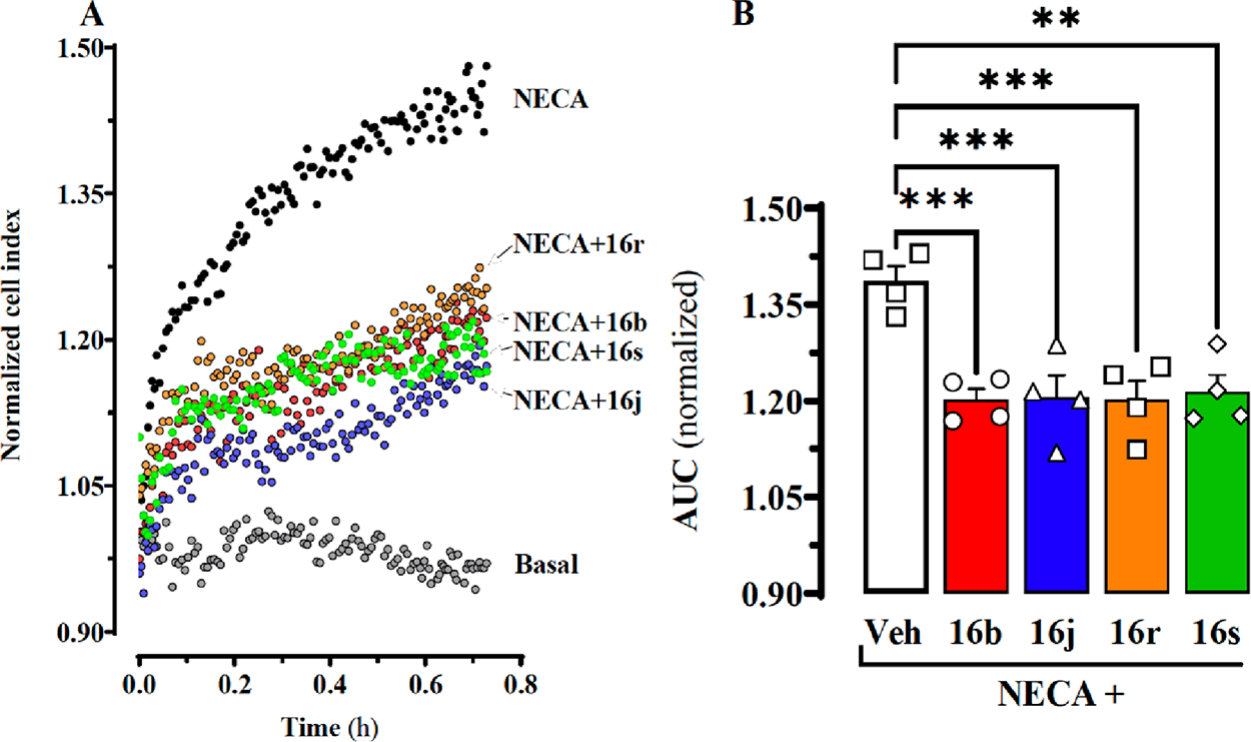
Blockade of A_2B_AR-mediated whole-cell label-free responses. (A) Representative example of the impedance signal obtained over the time upon incubation with A_2B_AR ligands. Cells were stimulated with vehicle or NECA (1 μM) in the absence or presence of **16b**, **16j**, **16r**, and **16s** (1 μM), 18 h after seeding, and the impedance signal recorded in real-time during 45 min. (B) The area under the curve (AUC) derived from the normalized cell index (NCI) shown in (A) was calculated for each condition. Data are expressed as mean ± SEM (*n* = 4). ***P < 0.001 and **P < 0.01, one-way ANOVA with a Dunnett’s multiple-comparison test when compared to vehicle (Veh).

To further evaluate the ability of **16b**, **16j**, **16r**, and **16s** to preclude A_2B_AR function in living cells, we took advantage of the cellular impedance label-free method, a widely accepted morphological and functional biosensor of cell status.^39^ Accordingly, whole-cell NECA-mediated impedance responses of A_2B_AR^SNAP^ cells were monitored in real time in the absence or presence of **16b**, **16j**, **16r**, and **16s**. Indeed, NECA (1 μM) induced a significant increase in A_2B_AR^SNAP^ cell impedance recordings, thus reflecting changes in cell morphology upon agonist challenge (Figure 4A). Importantly, **16b**, **16j**, **16r**, and **16s** partially blocked the NECA-induced impedance increase in A_2B_AR^SNAP^ cells (Figure 4A). When calculating the area under the curve (AUC) for the different experimental conditions, we can observe that **16b**, **16j**, **16r**, and **16s** significantly blocked NECA-induced changes in cellular impedance (Figure 4B). These results from an alternative cellular functional assay further confirmed that **16b**, **16j**, **16r**, and **16s** were able to block A_2B_AR agonist-mediated intrinsic activity.

Interestingly, the absolute **16b**, **16j**, **16r**, and **16s** antagonist potencies in the cAMP accumulation assay were greater (∼10-fold) than those determined in the radioligand binding assays, a fact that has been previously reported in HEK-293 cells stably overexpressing A_2B_AR.^40^ Indeed, this notion can be supported by the spare receptor theory defining that under some circumstances, the potency of a named agonist in functional assays is proportional to the receptor density, thus producing a discontinuity between drug occupancy and cell response.^41^ Our label-free experiments revealed a significant, but partial, blockade of a NECA-induced impedance increase in A_2B_AR^SNAP^ cells. Indeed, HEK-293 cells express endogenous Ado receptors as revealed by microarray analysis.^42^ Thus, while A_3_AR was not detected, the remaining A_2B_AR, A_2A_AR, and A_1_AR were identified in HEK-293 cells.^42^ Since NECA is a non-selective Ado receptor agonist showing affinity for A_2A_AR and A_1_AR (*K*_*i*_ values of 20 and 14 nM, respectively),^43^ it can be speculated that the remaining impedance increase in NECA-treated A_2B_AR^SNAP^ cells in the presence of **16b**, **16j**, **16r**, and **16s** can be mediated by endogenous A_2A_ and A_1_ ARs. These results might be interpreted as indirect evidence of selectivity for the A_2B_AR for compounds **16b**, **16j**, **16r**, and **16s**.

### Structure–Activity Relationship Analyses and Molecular Modeling

In this section, we examine the pharmacological data obtained for the newly synthesized series (Tables 2–6) and analyze the effect of halogenation in the structure–affinity (SAR) and structure–selectivity (SSR) relationships. All ligands were evaluated as racemic mixtures and comparative studies used as a reference of the parent series (Table 1). The SAR here outlined is then further assessed by FEP calculations in the context of a refined computational 3D model of hA_2B_AR.

The adenosinergic affinity profiles of the 80 alkyl 7/8-halo-4-heteroaryl-1,4-dihydro-benzo[4,5]imidazo[1,2-*a*]pyrimidine-3-carboxylates are presented in Tables 2–6. Up to 25 ligands combine attractive A_2B_AR affinity (*K*_*i*_ <50 nM) and exquisite subtype selectivity (>1000-fold), from which 14 ligands exhibit outstanding A_2B_AR affinity (*K*_*i*_ <10 nM) [e.g., compounds **16a**, **16b**, **16d**, **16i**, **16j**, **16k**, **16l**, **16r, 16s**, **16t** (Table 4), **17b**, **18b**, **18c**, and **18d** (Table 5)]. The available data reveal interesting and previously unexplored SAR trends, with derivatives bearing a halogen atom at position 8 of the heterotricyclic core collectively emerging as the most appealing A_2B_AR antagonists in these series (series **III** and **IV**, Tables 4 and 5). Compared to the congeners of the parent series (Table 1), 10 of the ligands here identified present improved pharmacodynamic and PK profiles, with ligands **16b**, **16j**, **16r**, and **16s** deserving particular attention, as they exhibit one-digit *K*_*i*_ values (3–5 nM) and excellent subtype selectivity, thus justifying the selection of these compounds for further intrinsic activity assays (see the previous section).

A comparative analysis of the binding data obtained for the parent series (Table 1) and ligands of series **I** (Table 2) evidenced that a simultaneous introduction of halogens at positions 7 and 8 has deleterious impact on A_2B_AR affinity. Moreover, it can be observed that this reduction in affinity is halogen dependent, with better affinity profiles observed for some 7,8-difluoro derivatives (Table 2, Cmpds **14a–d**) and the affinity decay being proportional to the atomic radius of the halogen (Table 2 and Supplementary Figure S3). Ligands bearing a thienyl ring at position 4 of the heterotricyclic scaffold are inactive with the only exception of the low-affinity **14p**. For the case of ligands containing a furyl group at R^4^, the A_2B_AR affinity seems to be highly dependent of the halogens present (Table 2). Thus, all ligands containing a furyl group of the 7,8-difluorinated scaffold (Cmpds **14a–d**) elicit potent A_2B_AR affinity (*K*_*i*_ = 11.9–52.9 nM) irrespective of the substitution pattern at the furan ring (2-furyl or 3-furyl) or the alkoxy residue in the ester moiety (R^3^). Although potent, the difluorinated derivatives **14a–d** show lower affinity (threefold) than their parent series analogues (Table 1). Similarly, in the subsets bearing chlorine atoms (Table 2, Cmpds **14i**–**p**), only the derivatives incorporating an optimal substituent at R^4^ and R^3^ (**14j** and **14k**) show moderate affinity. Following this trend, the only derivative retaining A_2B_AR affinity in the 7,8-dibromo subset is **14r** (*K*_*i*_ = 62.6 nM), containing at R^4^ and R^3^ the same substitution pattern of the lead compound **ISAM-140**, albeit with a 20-fold decrease in affinity.

**Table 1:**
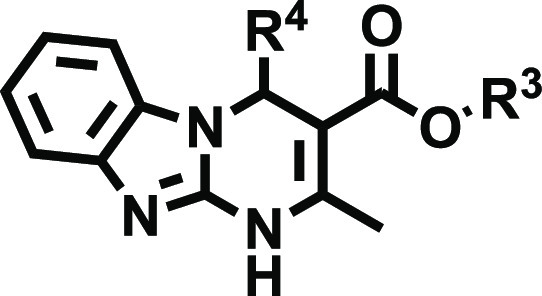
Structure and Adenosine Receptor Affinities of Alkyl 1,4-Dihydrobenzo [4,5]imidazo[1,2-*a*]pyrimidine-3-carboxylates (Prototype Series)^30,31^

				*K*_*i*_ (nM) or % at 1 μM		
compound	R^4^	R^3^	hA_1_^a^	hA_2A_^b^	hA_2B_^c^	hA_3_^d^
**I** (**ISAM-134)**	2-furyl	Et	5%	14%	12.0 ± 0.7	1%
**II** (**ISAM-140)**	2-furyl	*i*-Pr	20%	28%	3.49 ± 0.2	2%
**III** (**ISAM-141)**	3-furyl	Et	7%	11%	20.6 ± 1.1	1%
**IV** (**ISAM-142)**	3-furyl	*i*-Pr	12%	23%	11.4 ± 0.5	2%
**V**	2-thienyl	Et	8%	16%	484 ± 3	1%
**VI**	2-thienyl	*i*-Pr	1%	17%	371 ± 5	3%
**VII**	3-thienyl	Et	3%	10%	29.7 ± 1.2	2%
**VIII**	3-thienyl	*i*-Pr	11%	3%	29.34 ± 1.1	21%
**IX** (**ISAM-C032)**	4-oxazolyl	Et	5%	11%	8.10 ± 0.5	7%
**X**	4-oxazolyl	*i*-Pr	7%	19%	43.4 ± 1.3	16%
**DPCPX**			2.20 ± 0.2	157 ± 3	73.2 ± 1.4	1722 ± 11
**ZM241385**			683 ± 4	1.9 ± 0.1	65.7 ± 1.1	863 ± 4
**NECA**			14.0 ± 1	20.0 ± 3	2400 ± 35	6.20 ± 0.9

a Displacement of specific [^3^H]DPCPX binding in human CHO cells expressed as *K*_i_ in nanomolars (*n* = 3) or percentage displacement of specific binding at a concentration of 1 μM (*n* = 2).

b Displacement of specific [^3^H]4-(2-[7-amino-2-(2-furyl)[1,2,4]triazolo[2,3-*a*][1,3,5]triazin-5-ylamino]ethyl)phenol binding in human HeLa cells expressed as *K*_i_ in nanomolars (*n* = 3) or percentage displacement of specific binding at a concentration of 1 μM (*n* = 2).

c Displacement of specific [^3^H]DPCPX binding in human HEK-293 cells expressed as *K*_i_ in nanomolars (*n* = 3) or percentage displacement of specific binding at a concentration of 1 μM (*n* = 2).

d Displacement of specific [^3^H]NECA binding in human HeLa cells expressed as *K*_i_ in nanomolars (*n* = 3) or percentage displacement of specific binding at a concentration of 1 μM (*n* = 2).

**Table 2:**
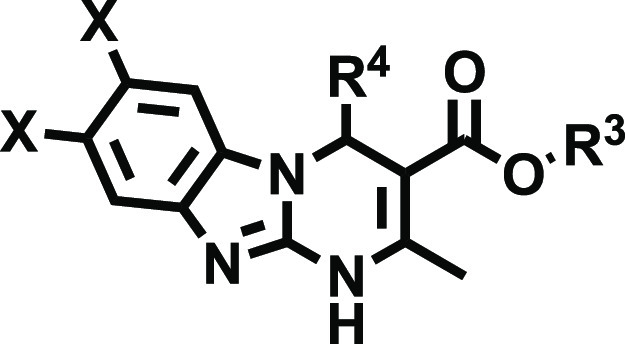
Structure and Adenosine Receptor Affinities of Series **I**: Alkyl 7,8-Dihalo-1,4-dihydrobenzo[4,5]imidazo[1,2-*a*]pyrimidine-3-carboxylates (Cmpds **14a–x**)

				*K*_*i*_ (nM) or % at 1 μM			
compound	R^4^	R^3^	X	hA_1_^a^	hA_2A_^b^	hA_2B_^c^	hA_3_^d^
**14a**	2-furyl	Et	F	1%	2%	44.1 ± 1.7	2%
**14b**	2-furyl	*i*-Pr	F	13%	22%	11.9 ± 1.1	14%
**14c**	3-furyl	Et	F	22%	36%	28.2 ± 0.8	17%
**14d**	3-furyl	*i*-Pr	F	17%	16%	52.9 ± 1.5	20%
**14e**	2-thienyl	Et	F	2%	3%	25%	5%
**14f**	2-thienyl	*i*-Pr	F	1%	1%	1%	10%
**14g**	3-thienyl	Et	F	1%	1%	519 ± 7	5%
**14h**	3-thienyl	*i*-Pr	F	1%	3%	43%	4%
**14i**	2-furyl	Et	Cl	2%	5%	9%	1%
**14j**	2-furyl	*i*-Pr	Cl	43%	12%	117 ± 5	7%
**14k**	3-furyl	Et	Cl	33%	34%	54.7 ± 3.2	6%
**14l**	3-furyl	*i*-Pr	Cl	4%	31%	36%	8%
**14m**	2-thienyl	Et	Cl	2%	1%	9%	11%
**14n**	2-thienyl	*i*-Pr	Cl	1%	4%	1%	9%
**14o**	3-thienyl	Et	Cl	5%	1%	9%	1%
**14p**	3-thienyl	*i*-Pr	Cl	2%	1%	359 ± 4	6%
**14q**	2-furyl	Et	Br	1%	17%	37%	2%
**14r**	2-furyl	*i*-Pr	Br	11%	28%	62.6 ± 1.9	21%
**14s**	3-furyl	Et	Br	2%	2%	7%	10%
**14t**	3-furyl	*i*-Pr	Br	1%	1%	32%	9%
**14u**	2-thienyl	Et	Br	2%	7%	12%	5%
**14v**	2-thienyl	*i*-Pr	Br	1%	1%	9%	3%
**14w**	3-thienyl	Et	Br	2%	7%	47%	4%
**14x**	3-thienyl	*i*-Pr	Br	4%	1%	9%	8%
**DPCPX**				2.20 ± 0.2	157 ± 3	73.2 ± 1.4	1722 ± 11
**ZM241385**				683 ± 4	1.9 ± 0.1	65.7 ± 1.1	863 ± 4
**NECA**				14.0 ± 1	20.0 ± 3	2400 ± 35	6.20 ± 0.9

a Displacement of specific [^3^H]DPCPX binding in human CHO cells expressed as *K*_i_ in nanomolars (*n* = 3) or percentage displacement of specific binding at a concentration of 1 μM (*n* = 2).

b Displacement of specific [^3^H]4-(2-[7-amino-2-(2-furyl)[1,2,4]triazolo[2,3-*a*][1,3,5]triazin-5-ylamino]ethyl)phenol binding in human HeLa cells expressed as *K*_i_ in nanomolars (*n* = 3) or percentage displacement of specific binding at a concentration of 1 μM (*n* = 2).

c Displacement of specific [^3^H]DPCPX binding in human HEK-293 cells expressed as *K*_i_ in nanomolars (*n* = 3) or percentage displacement of specific binding at a concentration of 1 μM (*n* = 2).

d Displacement of specific [^3^H]NECA binding in human HeLa cells expressed as *K*_i_ in nanomolars (*n* = 3) or percentage displacement of specific binding at a concentration of 1 μM (*n* = 2).

The adenosinergic profile obtained for series **II** is presented in Table 3. Here, introduction of a halogen at position 7 reproduced some of the SAR trends detected in series **I** (Table 2). While some attractive ligands can be identified (e.g., **15b** and **15c**, *K*_*i*_ = 12.2 and 25.0 nM, respectively), a comparison with the parent series (Table 1) revealed that halogenation at position 7 is deleterious. As for series **I**, the effect of halogenation relied on the nature of the halogen, with fluor being the best tolerated (Table 3, Cmpds **15a–d**). Similarly, most derivatives bearing thienyl groups remain inactive. For series bearing chloro and bromo atoms at position 7, only optimal pair combinations at R^4^ and R^3^ (e.g., 2-furyl and isopropyl, 3-furyl and ethyl, respectively) produced ligands with moderate (*K*_*i*_ ∼ 45 nM) affinity (Table 3, Cmpds **15j**, **15k**, **15r**, and **15s**). This trend is also observed for 7-fluoro derivatives (Table 3, Cmpds **15a**–**15d**), while in this subset, all furyl-containing derivatives exhibited attractive A_2B_AR affinity (*K*_*i*_ = 12.2–136 nM).

**Table 3:**
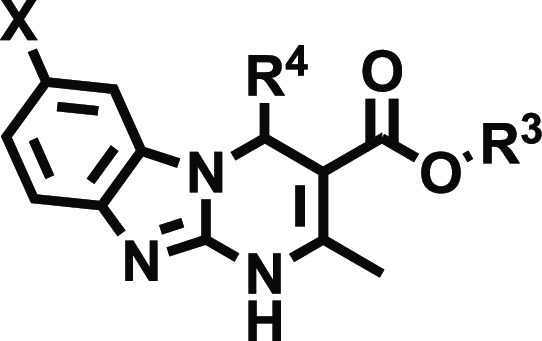
Structure and Adenosine Receptor Affinities of Series **II**: Alkyl 7-Halo-1,4-dihydrobenzo[4,5]imidazo[1,2-*a*]pyrimidine-3-carboxylates (Cmpds **15a–x**)

				*K*_*i*_ (nM) or % at 1 μM			
compound	R^4^	R^3^	X	hA_1_^a^	hA_2A_^b^	hA_2B_^c^	hA_3_^d^
**15a**	2-furyl	Et	F	16%	2%	67.5 ± 3.1	10%
**15b**	2-furyl	*i*-Pr	F	32%	3%	12.2 ± 1.2	5%
**15c**	3-furyl	Et	F	26%	21%	25.0 ± 1.5	5%
**15d**	3-furyl	*i*-Pr	F	25%	11%	136 ± 7	6%
**15e**	2-thienyl	Et	F	25%	1%	37%	11%
**15f**	2-thienyl	*i*-Pr	F	11%	2%	23%	6%
**15g**	3-thienyl	Et	F	7%	1%	1031 ± 95	3%
**15h**	3-thienyl	*i*-Pr	F	1%	1%	764 ± 11	1%
**15i**	2-furyl	Et	Cl	26%	2%	18%	4%
**15j**	2-furyl	*i*-Pr	Cl	667 ± 12	17%	44.3 ± 1.4	5%
**15k**	3-furyl	Et	Cl	22%	30%	47.3 ± 1.9	3%
**15l**	3-furyl	*i*-Pr	Cl	6%	4%	50%	3%
**15m**	2-thienyl	Et	Cl	2%	3%	1%	1%
**15n**	2-thienyl	*i*-Pr	Cl	1%	3%	7%	1%
**15o**	3-thienyl	Et	Cl	4%	2%	50%	3%
**15p**	3-thienyl	*i*-Pr	Cl	7%	9%	48%	3%
**15q**	2-furyl	Et	Br	22%	21%	48%	5%
**15r**	2-furyl	*i*-Pr	Br	46%	1%	273 ± 4	6%
**15s**	3-furyl	Et	Br	10%	1%	318 ± 6	19%
**15t**	3-furyl	*i*-Pr	Br	11%	3%	26%	12%
**15u**	2-thienyl	Et	Br	1%	5%	1%	4%
**15v**	2-thienyl	*i*-Pr	Br	1%	6%	11%	8%
**15w**	3-thienyl	Et	Br	4%	3%	43%	3%
**15x**	3-thienyl	*i*-Pr	Br	1%	1%	10%	1%
**DPCPX**				2.20 ± 0.2	157 ± 3	73.2 ± 1.4	1722 ± 11
**ZM241385**				683 ± 4	1.9 ± 0.1	65.7 ± 1.1	863 ± 4
**NECA**				14.0 ± 1	20.0 ± 3	2400 ± 35	6.20 ± 0.9

a Displacement of specific [^3^H]DPCPX binding in human CHO cells expressed as *K*_i_ in nanomolars (*n* = 3) or percentage displacement of specific binding at a concentration of 1 μM (*n* = 2).

b Displacement of specific [^3^H]4-(2-[7-amino-2-(2-furyl)[1,2,4]triazolo[2,3-*a*][1,3,5]triazin-5-ylamino]ethyl)phenol binding in human HeLa cells expressed as *K*_i_ in nM (*n* = 3) or percentage displacement of specific binding at a concentration of 1 μM (*n* = 2).

c Displacement of specific [^3^H]DPCPX binding in human HEK-293 cells expressed as *K*_i_ in nanomolars (*n* = 3) or percentage displacement of specific binding at a concentration of 1 μM (*n* = 2).

d Displacement of specific [^3^H]NECA binding in human HeLa cells expressed as *K*_i_ in nanomolars (*n* = 3) or percentage displacement of specific binding at a concentration of 1 μM (*n* = 2).

**Figure 5:**
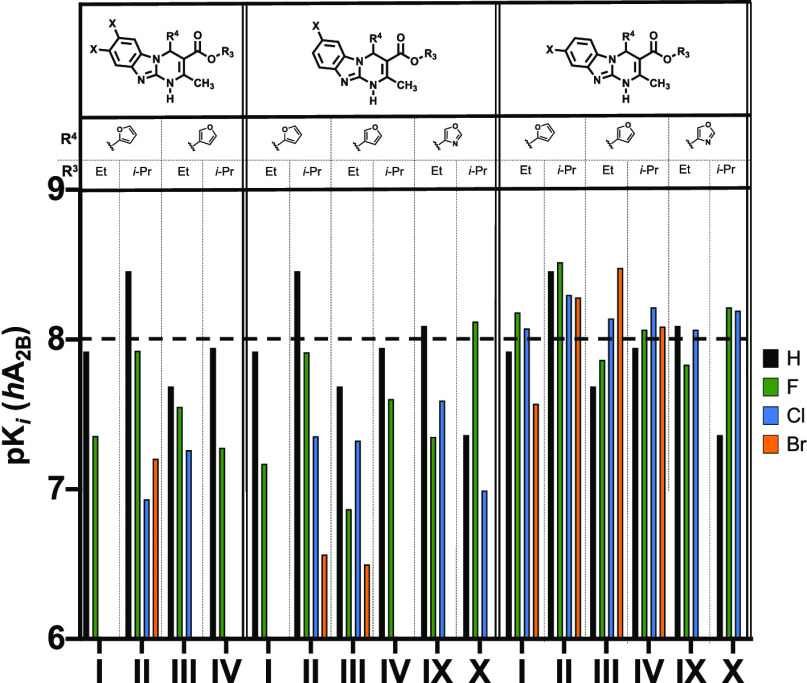
Interleaved bar chart showing A_2B_AR p*K*_*i*_ [−log(*K*_*i*_)] for the halogenation of furyl and oxazolyl scaffolds (represented at the *x*-axis as **I**–**IV** and **IX**–**X**). Black: non-halogenated, green: fluorinated, cyan: chlorinated, and orange: brominated compounds.

An evaluation of series **III** (Table 4) enabled the identification of the most potent (*K*_*i*_ < 10 nM) A_2B_AR antagonists described in this study. It also allowed to envision the significant effect of halogen substitution at position 8 and its rationalization from a molecular modeling perspective. In contrast to previous series, halogenation at position 8 systematically improved the A_2B_AR affinity irrespective of the nature of the halogen atom (Table 4 and Figure 5). Indeed, most 8-halo derivatives have measurable A_2B_AR affinity values (Table 4). All 8-halogenated derivatives bearing a furan ring at position 4 exhibit excellent A_2B_AR affinity (*K*_*i*_ = 3.05–26.9 nM), indeed generally superior by 4–10-fold to their analogues in the parent series (Figure 5). A similar trend, but with attenuated affinity, is observed for thiophene-based analogues (*K*_*i*_ = 61.1–911 nM). It should also be noticed that for thienyl derivatives, the introduction of a halogen at position 8 does not improve A_2B_AR affinities as compared to the non-halogenated parent compounds (Table 1). A direct comparison of the A_2B_AR affinity profile (p*K*_*i*_) of selected derivatives of different series is presented in Figure 5.

**Table 4:**
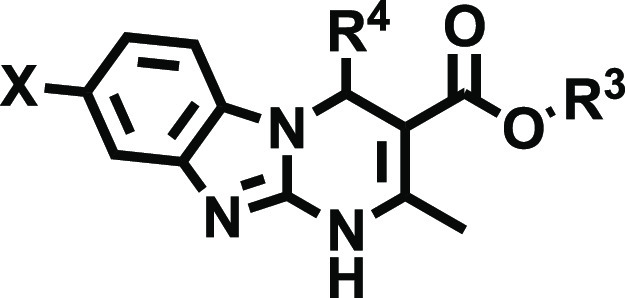
Structure and Adenosine Receptor Affinities of Series **III**: Alkyl 8-Halo-1,4-dihydrobenzo[4,5]imidazo[1,2-*a*]pyrimidine-3-carboxylates (Cmpds **16a–x**)

				*K*_*i*_ (nM) or % at 1 μM			
compound	R^4^	R^3^	X	hA_1_^a^	hA_2A_^b^	hA_2B_^c^	hA_3_^d^
**16a**	2-furyl	Et	F	10%	29%	6.60 ± 1.0	2%
**16b** (**ISAM-163)**	2-furyl	*i*-Pr	F	11%	2%	3.05 ± 0.7	3%
**16c**	3-furyl	Et	F	29%	21%	13.7 ± 0.9	2%
**16d**	3-furyl	*i*-Pr	F	14%	31%	8.60 ± 0.5	4%
**16e**	2-thienyl	Et	F	1%	3%	724 ± 8	3%
**16f**	2-thienyl	*i*-Pr	F	2%	1%	668 ± 6	7%
**16g**	3-thienyl	Et	F	1%	1%	187 ± 2	3%
**16h**	3-thienyl	*i*-Pr	F	2%	1%	75.5 ± 3.2	5%
**16i**	2-furyl	Et	Cl	23%	10%	8.42 ± 1.4	4%
**16j** (**ISAM-161)**	2-furyl	*i*-Pr	Cl	35%	24%	5.03 ± 0.3	2%
**16k**	3-furyl	Et	Cl	31%	43%	7.21 ± 0.4	4%
**16l** (**ISAM-M89A)**	3-furyl	*i*-Pr	Cl	27%	176 ± 4	6.10 ± 0.7	1%
**16m**	2-thienyl	Et	Cl	3%	2%	41%	1%
**16n**	2-thienyl	*i*-Pr	Cl	2%	2%	837 ± 11	1%
**16o**	3-thienyl	Et	Cl	2%	7%	911 ± 14	8%
**16p**	3-thienyl	*i*-Pr	Cl	7%	7%	165 ± 9	4%
**16q**	2-furyl	Et	Br	18%	29%	26.9 ± 0.8	33%
**16r** (**ISAM-157)**	2-furyl	*i*-Pr	Br	25%	2%	5.23 ± 0.4	10%
**16s** (**ISAM-M114A)**	3-furyl	Et	Br	1%	37%	3.32 ± 0.4	25%
**16t**	3-furyl	*i*-Pr	Br	2%	509 ± 6	8.20 ± 1.1	2%
**16u**	2-thienyl	Et	Br	6%	3%	55%	6%
**16v**	2-thienyl	*i*-Pr	Br	1%	1%	115 ± 3	1%
**16w**	3-thienyl	Et	Br	1%	9%	537 ± 8	1%
**16x**	3-thienyl	*i*-Pr	Br	2%	1%	61.1 ± 1.8	4%
**DPCPX**				2.20 ± 0.2	157 ± 3	73.2 ± 1.4	1722 ± 11
**ZM241385**				683 ± 4	1.9 ± 0.1	65.7 ± 1.1	863 ± 4
**NECA**				14.0 ± 1	20.0 ± 3	2400 ± 35	6.20 ± 0.9

a Displacement of specific [^3^H]DPCPX binding in human CHO cells expressed as *K*_i_ in nanomolars (*n* = 3) or percentage displacement of specific binding at a concentration of 1 μM (n = 2).

b Displacement of specific [^3^H]4-(2-[7-amino-2-(2-furyl)[1,2,4]triazolo[2,3-*a*][1,3,5]triazin-5-ylamino]ethyl)phenol binding in human HeLa cells expressed as *K*_i_ in nanomolars (*n* = 3) or percentage displacement of specific binding at a concentration of 1 μM (*n* = 2).

c Displacement of specific [^3^H]DPCPX binding in human HEK-293 cells expressed as *K*_i_ in nanomolars (*n* = 3) or percentage displacement of specific binding at a concentration of 1 μM (*n* = 2).

d Displacement of specific [^3^H]NECA binding in human HeLa cells expressed as *K*_i_ in nanomolars (*n* = 3) or percentage displacement of specific binding at a concentration of 1 μM (*n* = 2).

The pharmacological data obtained for the exploratory derivatives bearing a 4-oxazolyl residue at position 4 of the tricyclic core (series **IV**, Table 5) introduced new SAR perspectives. Although in general this series reproduced the most prominent SAR trends discussed for the previous series, the effect of halogenation seems to be less marked if compared to their parent compounds (Table 1>, Cmpds **IX** and **X**). Collectively, 8-halo derivatives in this series (Table 5, Cmpds **18a**–**18d**) exhibit excellent A_2B_AR affinity (*K*_*i*_ 6.10–14.7 nM). A comparative analysis reveals that the affinity data in this series is lightly superior to the 7-halo analogues (Table 5, Cmpds **17a**–**17d**) or the parent compounds in the prototype series (Table 1, Cmpds **IX** and **X**). These data highlight the interest of the 4-oxazolyl moiety as a privileged non-furane core at A_2B_AR, which, in addition to a superior metabolic stability profile, can capture the two productive interactions predicted for 2-furyl and 3-furyl rings within the A_2B_AR orthosteric binding site. This allows for better accommodation of the compounds at A_2B_AR (Supplementary Figure S4), even allowing a successful fitting of the 7-halo derivatives (Table 5).

**Table 5:**
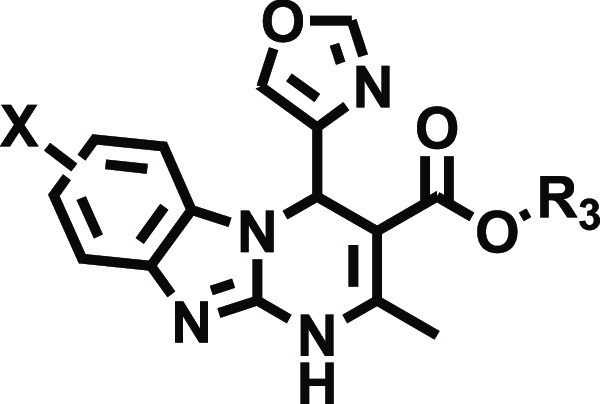
Structure and Adenosine Receptor Affinities of Series **IV**: Alkyl 7/8-Halo 4-(oxazol-4-yl)-1,4-dihydrobenzo[4,5]imidazo[1,2-*a*]pyrimidine-3-carboxylates (Cmpds **17a**–**d** and **18a**–**d**)

				*K*_*i*_ (nM) or % at 1 μM			
compound		R^3^	X	hA_1_^a^	hA_2A_^b^	hA_2B_^c^	hA_3_^d^
**17a**		Et	7-F	43%	4%	44.7 ± 1.4	26%
**17b** (**ISAM-R324B)**		*i*-Pr	7-F	34%	19%	7.60 ± 0.6	22%
**17c**		Et	7-Cl	227 nM	14%	25.6 ± 1.2	11%
**17d**		*i*-Pr	7-Cl	27%	1%	102 ± 7	20%
**18a**		Et	8-F	25%	16%	14.7 ± 1.1	16%
**18b** (**ISAM-R324A)**		*i*-Pr	8-F	29%	20%	6.10 ± 0.3	20%
**18c** (**ISAM-R316A)**		Et	8-Cl	30%	13%	8.60 ± 0.4	15%
**18d** (**ISAM-R319A)**		*i*-Pr	8-Cl	23%	35%	6.40 ± 0.7	23%
**DPCPX**				2.20 ± 0.2	157 ± 3	73.2 ± 1.4	1722 ± 11
**ZM241385**				683 ± 4	1.9 ± 0.1	65.7 ± 1.1	863 ± 4
**NECA**				14.0 ± 1	20.0 ± 3	2400 ± 35	6.20 ± 0.9

a Displacement of specific [^3^H]DPCPX binding in human CHO cells expressed as *K*_i_ in nanomolars (*n* = 3) or percentage displacement of specific binding at a concentration of 1 μM (*n* = 2).

b Displacement of specific [^3^H]4-(2-[7-amino-2-(2-furyl)[1,2,4]triazolo[2,3-*a*][1,3,5]triazin-5-ylamino]ethyl)phenol binding in human HeLa cells expressed as *K*_i_ in nanomolars (*n* = 3) or percentage displacement of specific binding at a concentration of 1 μM (*n* = 2).

c Displacement of specific [^3^H]DPCPX binding in human HEK-293 cells expressed as *K*_i_ in nanomolars (*n* = 3) or percentage displacement of specific binding at a concentration of 1 μM (*n* = 2).

d Displacement of specific [^3^H]NECA binding in human HeLa cells expressed as *K*_i_ in nanomolars (*n* = 3) or percentage displacement of specific binding at a concentration of 1 μM (*n* = 2).

Due to their dual A_2A_/A_2B_AR profile, two ligands (Table 4, Cmpds **16l** and **16t**) caught our interest during the analysis of SAR trends within the series of 8-halo derivatives. Both ligands combine the excellent A_2B_AR affinity of this subset (*K*_*i*_ = 6.10 and 8.20 nM, respectively), with an incipient affinity by A_2A_AR (*K*_*i*_ = 176 and 509 nM, respectively). While sharing a common structural pattern (e.g., a 3-furyl ring at R^4^ and isopropyl group in the alkoxy residue of the ester function at R^3^), they differ in the halogen atom at position 8 (Cl vs Br). Despite being equipotent at A_2B_AR, the important differences in A_2A_AR affinity (threefold) suggest that the chlorine atom is better tolerated for the binding at A_2A_AR. With further studies currently in progress to explore in depth the structural requirements for dual A_2A_AR/A_2B_AR antagonism, substitution at position 8 appears to be a key structural requirement for dual blockade of both receptors. It should be noticed that the dual A_2A_AR/A_2B_AR profile of **16l** is interesting in the context of cancer (immuno)therapy, with two compounds showing similar profiles in clinical trials.^44^ Indeed, this dual profile allowed us to include compound **16l** in a recent study with ex vivo assays to study the potential of the A_2B_AR antagonism in the context of cancer immunotherapy.^36^

### Enantiospecific Binding to A_2B_AR

**Figure 6:**
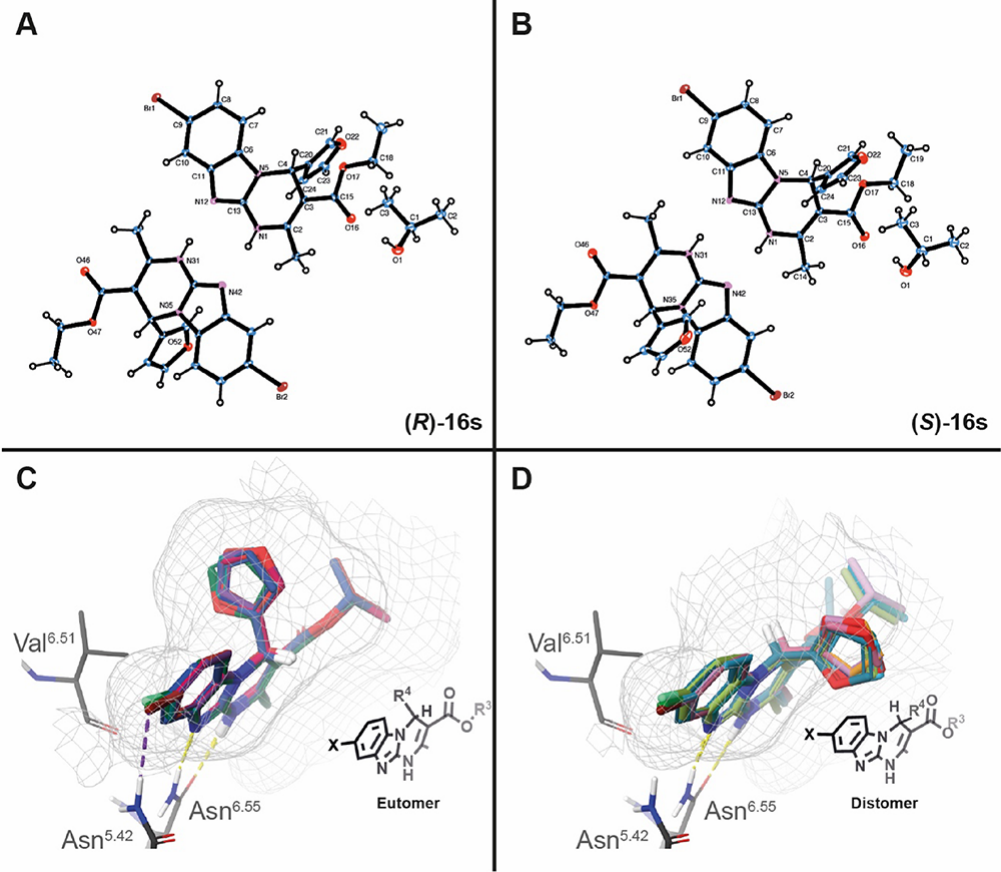
(A) X-ray crystal structure of **(***R***)**-**16s** (CCDC: 2048281). (B) **(***S***)**-**16s** (CCDC: 2047365). (C) Docking superposition for eutomers **(***S***)**-**ISAM-140**, **(***S***)**-**16b**, **(***S***)**-**16j**, **(***R***)**-**16l**, **(***S***)**-**16r**, **(***R***)**-**16s**, and **(***S***)**-**18c** (A_2B_AR homology model from A_2A_AR, PDB: 3EML). Highlight the formation of a halogen bridge with 8-chlorine and 8-bromine compounds (Asn^5.42^). (D) Docking superposition for distomers **(***R***)**-**ISAM-140**, **(***R***)**-**16b**, **(***R***)**-**16j**, **(***S***)**-**1i (***R***)**-**16r**, **(***S***)**-**16s**, and **(***R***)**-**18c** (A_2B_AR homology model from A_2A_AR, PDB: 3EML). Gray mesh represents the A_2B_AR binding site surface.

**Figure 7:**
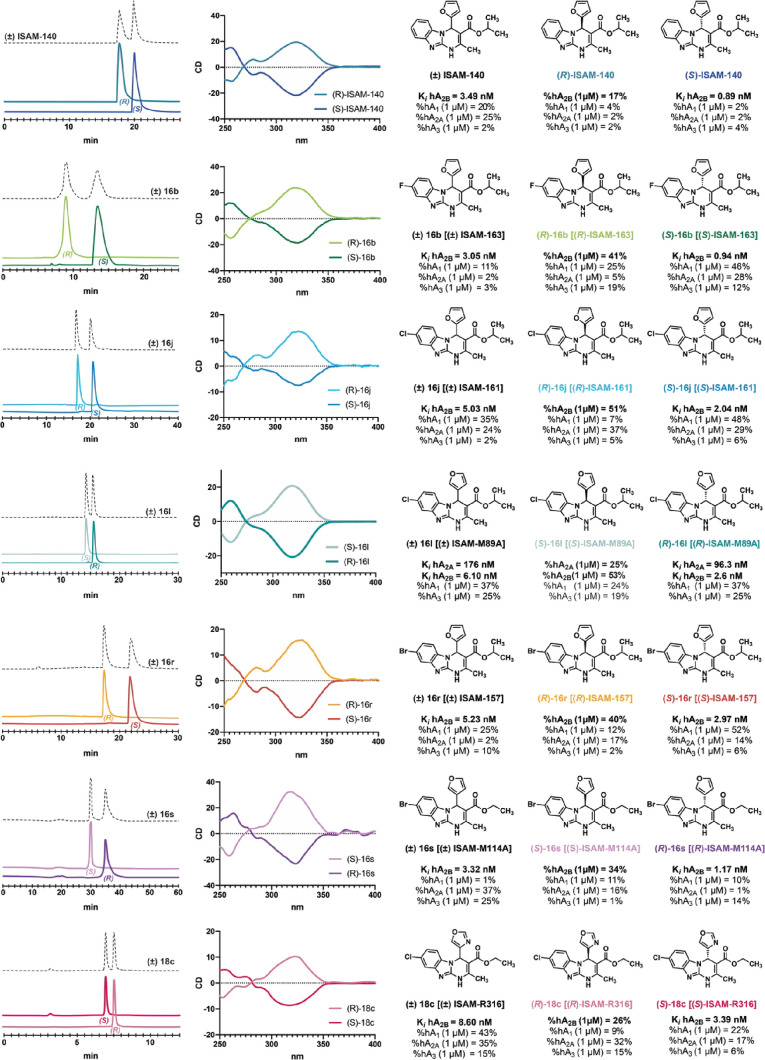
Chiral HPLC traces, circular dichroism spectra, and binding data of selected racemic ligands (**ISAM-140**, **16b**, **16j**, **16l**, **16r**, **16s**, and **18c**) and its enantiomers.

The four series of A_2B_AR antagonists herein documented were initially obtained and tested as racemates (Tables 2–5). The stereocenter within the heterocyclic core is a signature element of our ligands,^25,31,33−35^ which plays an important role for the recognition within the A_2B_AR binding pocket, providing structural singularity with respect to known planar A_2B_AR ligands (e.g., xanthines). Early molecular models and FEP calculations, supported by experimental data, predicted that only one enantiomer should bind to the orthosteric site of A_2B_AR. To further elucidate the molecular basis underlying enantioselective recognition in these series and to expand the repertoire of stereoselective A_2B_AR antagonists, we proceeded with enantiomeric separation, assignment, and biological evaluation (Table 6) of a representative subset of A_2B_AR antagonists. A validated approach using chiral HPLC, circular dichroism (CD) spectroscopy, and X-ray crystallography (Figure 6)^25,31,33−35^ was employed to separate and assign the configuration of six representative A_2B_AR antagonists [(±)**-16b**, (±)**-16j**, (±)**-16l**, (±)**-16r**, (±)**-16s**, and (±)**-18c**] and the prototype ligand of the parent series [(±)**ISAM-140**]. Semipreparative HPLC separation of the selected racemic ligands on a chiral stationary phase (Figure 6 and Experimental section) afforded each enantiomer with excellent stereochemical purity (i.e., >97%). As documented for different Biginelli-based scaffolds,^45^ the sign of the distinctive CD activity of the enamide group (around 300 nm) allows the unequivocal assignment of the absolute configuration of each enantiomer (Figure 7). At that wavelength, enantiomers showing a negative Cotton effect (intense line) contain the pentagonal heterocycle (furan or oxazole) backward while the stereoisomers giving a positive Cotton effect (clear line) contain the heterocyclic core forward. As a complement of these studies, a structural analysis of monocrystals of **(***S***)-16s** and **(***R***)-16s**, through X-ray crystallography (Figure 6A,B), provided additional experimental evidence corroborating the CD-assisted stereochemical configuration (Figure 7).

**Table 6:**
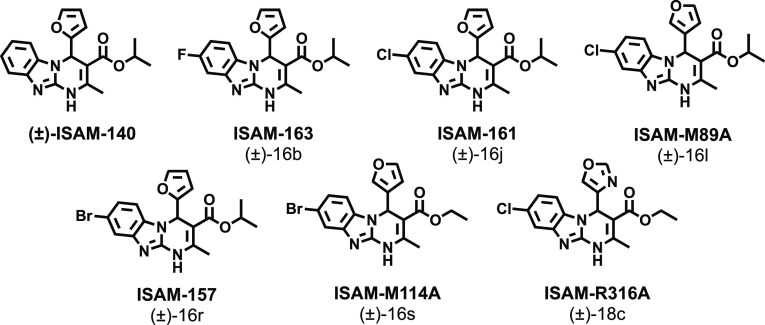
Structure and Adenosine Receptor Affinities of Racemic and Enantiomers of Selected Ligands

				*K*_*i*_ (nM) or % at 1 μM			
compound	R^4^	R^3^	R^8^	hA_1_^a^	hA_2A_^b^	hA_2B_^c^	hA_3_^d^
**(±)-ISAM-140**	2-furyl	*i*-Pr	H	20%	28%	3.49 ± 0.2	2%
**ISAM-140**	2-furyl	*i*-Pr	H	14%	7%	17%	3%
**(***S***)-ISAM-140**	2-furyl	*i*-Pr	H	2%	12%	0.89 ± 0.2	4%
**(±)-16b [(±)-ISAM-163]**	2-furyl	*i*-Pr	F	21%	2%	3.05 ± 0.7	3%
**(***R***)-16b(***R***)-ISAM-163]**	2-furyl	*i*-Pr	F	15%	5%	41%	19%
**(***S***)-16b [(***S***)-ISAM-163]**	2-furyl	*i*-Pr	F	26%	11%	0.94 ± 0.1	12%
**(±)-16j [(±)-ISAM-161]**	2-furyl	*i*-Pr	Cl	35%	24%	5.03 ± 0.3	2%
**(***R***)-16j [(***R***)-ISAM-161]**	2-furyl	*i*-Pr	Cl	7%	37%	51%	5%
**(***S***)-16j [(***S***)-ISAM-161]**	2-furyl	*i*-Pr	Cl	48%	19%	2.04 ± 0.2	6%
**(±)-16l [(±)-ISAM-M89A]**	3-furyl	*i*-Pr	Cl	27%	176 ± 4	6.10 ± 0.7	1%
**(***S***)-16l [(***S***)-ISAM-M89A]**	3-furyl	*i*-Pr	Cl	24%	25%	53%	19%
**(***R***)-16l [(***R***)-ISAM-M89A]**	3-furyl	*i*-Pr	Cl	37%	96.3 ± 6	2.6 ± 0.3	24%
**(±)-16r [(±)-ISAM-157]**	2-furyl	*i*-Pr	Br	25%	2%	5.23 ± 0.4	10%
**(***R***)-16r [(***R***)-ISAM-157]**	2-furyl	*i*-Pr	Br	12%	7%	35%	2%
**(***S***)-16r [(***S***)-ISAM-157]**	2-furyl	*i*-Pr	Br	32%	14%	2.97 ± 0.4	6%
**(±)-16s [(±)-ISAM-M114A]**	3-furyl	Et	Br	1%	37%	3.32 ± 0.4	25%
**(***R***)-16s [(***R***)-ISAM-M114A]**	3-furyl	Et	Br	10%	12%	1.17 ± 0.1	14%
**(***S***)-16s [(***S***)-ISAM-M114A]**	3-furyl	Et	Br	11%	16%	34%	10%
**(±)-18c [(±)-ISAM-R316A]**	4-oxazolyl	Et	Cl	30%	13%	8.60 ± 0.4	15%
**(***R***)-18c [(***R***)-ISAM-R316A]**	4-oxazolyl	Et	Cl	9%	32%	26%	15%
**(***S***)-18c [(***S***)-ISAM-R316A]**	4-oxazolyl	Et	Cl	22%	17%	3.39 ± 0.2	6%
**DPCPX**				2.20 ± 0.2	157 ± 3	73.2 ± 1.4	1722 ± 11
**ZM241385**				683 ± 4	1.9 ± 0.1	65.7 ± 1.1	863 ± 4
**NECA**				14.0 ± 1	20.0 ± 3	2400 ± 35	6.20 ± 9

a Displacement of specific [^3^H]DPCPX binding in human CHO cells expressed as *K*_i_ in nanomolars (*n* = 3) or percentage displacement of specific binding at a concentration of 1 μM (*n* = 2).

b Displacement of specific [^3^H]4-(2-[7-amino-2-(2-furyl)[1,2,4]triazolo[2,3-*a*][1,3,5]triazin-5-ylamino]ethyl)phenol binding in human HeLa cells expressed as *K*_i_ in nanomolars (*n* = 3) or percentage displacement of specific binding at a concentration of 1 μM (*n* = 2).

c Displacement of specific [^3^H]DPCPX binding in human HEK-293 cells expressed as *K*_i_ in nanomolars (*n* = 3) or percentage displacement of specific binding at a concentration of 1 μM (*n* = 2).

d Displacement of specific [^3^H]NECA binding in human HeLa cells expressed as *K*_i_ in nanomolars (*n* = 3) or percentage displacement of specific binding at a concentration of 1 μM (*n* = 2).

The affinity profile of the obtained enantiomers at the four human ARs, together with the corresponding racemic ligands, is shown in Table 6. Inspection of the obtained data confirm that the observed A_2B_AR affinity within the racemic ligands is exclusively due to one of the stereoisomers, in line with the A_2B_AR enantiospecific recognition model documented for structurally analogous series.^25,31,33,34^ In all cases, the eutomers (which contain the pentagonal core backward) are nearly twofold more potent than their corresponding racemate, whereas the other stereoisomer is devoid in all cases of any affinity at the four ARs (Table 6 and Figure 7). Particularly noticeable are the two eutomers [**(***S***)-16b** and **(***S***)-ISAM-140**] with sub-nanomolar A_2B_AR affinities. The eutomer **(***R***)-16l**, containing the pentagonal core backward (Figure 6C), emerges as the first example of a dual A_2A_/A_2B_AR ligand exhibiting enantiospecific recognition at both receptors, since its enantiomer (which contain the pentagonal core forward, Figure 6D) is completely inactive. The enantioselective recognition reported here for **(***R***)-16l** has inspired the design of a new series of non-planar dual antagonists that will be reported on due time.

**Table 7:**
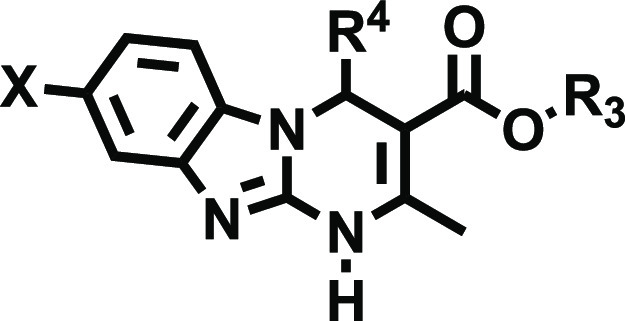
Functional Data of Compounds **16b**, **16j**, **16r**, and **16s**

compound	R^4^	R^3^	X	*K*_*i*_ (hA_2B_)	*K*_B_ (hA_2B_)
**(±)-16b (ISAM-163)**	2-furyl	*i-*Pr	F	3.05 ± 0.7	0.80 ± 0.2
**(±)-16j (ISAM-161)**	2-furyl	*i-*Pr	Cl	5.03 ± 0.3	0.60 ± 0.2
**(±)-16r (ISAM-157)**	2-furyl	*i-*Pr	Br	5.23 ± 0.4	1.70 ± 0.6
**(±)-16s (ISAM-M114A)**	3-furyl	Et	Br	3.32 ± 0.4	1.50 ± 0.5

**Figure 8:**
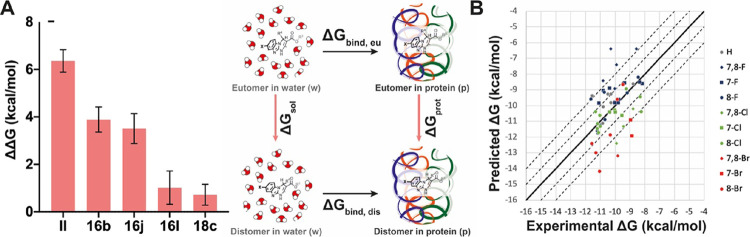
(A) ΔΔG data for eutomer to distomer transition (positive ΔΔG). (B) ΔG FEP correlation between all compounds showing *K*_*i*_ in pharmacology experimental assays, grouped by position and halogen.

To provide a further structural rationale, we calculated the relative binding free energy difference between each pair of enantiomers for compounds **ISAM-140**, **16b**, **16j**, **16l**, and **18c** (Figure 8A). The corresponding FEP simulations were run taking as starting point the binding model proposed earlier for this scaffold,^25^ illustrated in Figure 6C,D. The binding mode of the eutomers (Figure 6C) has been largely explored with FEP simulations on previous series, allowing a rationalization of the observed SAR.^31,33^ In the present computational analysis, we assumed an analogous binding orientation for the distomer (Figure 6D), although there can be other possibilities, which is difficult to assess given the inactive profile of these enantiomers. Nevertheless, the qualitative agreement with the experimental profile is remarkable, as the simulations indicate a clear binding preference for the eutomer in all five cases (Figure 8A). These data confirm our previous computational models for enantiospecific recognition of structurally related Biginelli-based scaffolds,^46^ which was used to assess the growth of these scaffolds.

### Docking and FEP Simulations on the Eutomers

**Figure 9:**
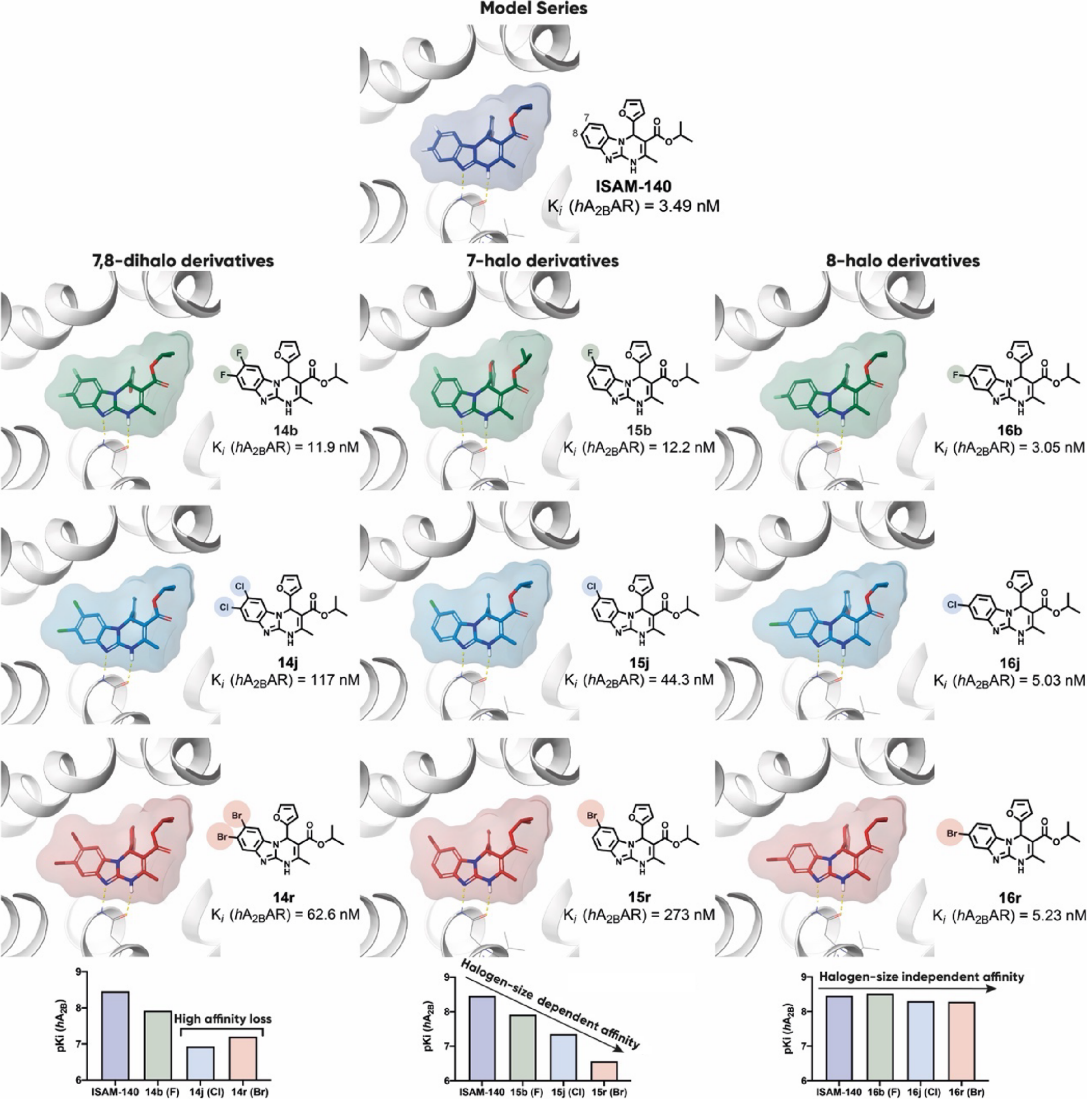
Docking pose for series **I** (7,8-dihalo derivatives, left), series **II** (7-halo derivatives, center), and **III** (8-halo derivatives, right), shown for the subset of derivatives with R^4^ = 2-furyl and R^3^ = *i*-Pr, together with their affinity profile at A_2B_AR (homology model from A_2A_AR, PDB: 3EML).

Using the stereospecific binding mode established in the previous stage, we set up a systematic scheme of FEP simulations aimed to provide structural understanding to the observed SAR. To this end, we selected all 56 compounds with measured experimental affinity for *h*A_2B_AR (*K*_*i*_ (<1000 nM) and ran a series of alchemical transformations to obtain relative binding free energies (RBFE), by FEP transformation of the substituents on positions 7 and 8, while retaining the same substituents on R^3^ and R.^4^ Within each congeneric series (as defined in Table 1 for reference compounds), the compound pairs for the FEP simulations were defined by our mapping algorithm, and the absolute binding free energies (ABFE) were estimated following a cycle closure correction, taking the experimental affinity value of one compound as a reference (see Supplementary Table S3). With this approach, we maintained simplicity within the alchemical transformations, ensuring a good convergence (average SEM = 0.44 kcal/mol). The pulled results obtained for all congeneric series are summarized in Figure 8C and Supplementary Figure S5. The correlation between calculated and experimental binding free energies is very good, according to the low value for the mean unassigned error (MUE = 1.08 kcal/mol). A closer look into the data revealed that most of this unassigned error is caused by the group of brominated compounds (red symbols, Figure 8B), which show a tendency to overprediction. The binding model proposed is thus reinforced by these calculations, which correctly reproduce the global SAR outlined in the previous section. Experimentally, we observed that 7-halogenated substitutions show halogen size-dependent A_2B_AR affinity. The pattern observed for the 7,8-dihalogenated compounds seems to confirm this tendency, while 8-halogenated compounds show a halogen-size-independent A_2B_AR affinity (Figure 9). According to our binding model, the bulkier Cl and Br substituents on the last position can make a halogen-bond interaction with Asn^5.42^, counterbalancing the excess of volume on position 8, something that is not possible when the halogen is on position 7, resulting in a decrease in affinity proportional to the volume of the buried halogen atom (Supplementary Figure S4).

### Preliminary ADMET Exploration

An early understanding of the ADMET (absorption, distribution, metabolism, excretion, and toxicity) profile of prototypical new lead compounds is crucial to reducing attrition and accelerating upstream drug discovery programs.^47,48^ As reported in the bibliography, halogenation, and particularly fluorination, usually enhances the metabolic stability of chemical scaffolds.^49,50^ To preliminarily explore the in vitro ADMET profile of the series documented here, seven representative A_2B_AR antagonists (**14b**, **15j**, **16b**, **16j**, **16l**, **16r**, and **18b**) were selected to evaluate their solubility, liver microsome stability, and inhibitory profile toward a prototypic cytochrome enzyme (CYP3A4). The selected compounds allow not only a preliminary evaluation of the impact of mono- or di-halogenation (positions 7, 8, or 7,8) but also to analyze the effect of different atoms (F, Cl, Br) on the PK parameters evaluated, while the inclusion of ligand **18b** allowed us to assess the effect of fluorination on a representative non-furan A_2B_AR antagonist. Table 8 shows the PK data obtained for the selected compounds; for comparison, the PK data available for the non-halogenated reference compounds (Cmpds **II** and **X**) is included in Table 8, together with the adenosinergic data (*h*A_2B_*K*_*i*_ in nanomolars, and *h*A_2A_*K*_*i*_ for **16l**) in order to facilitate an integrated analysis for these compounds.

**Table 8:**
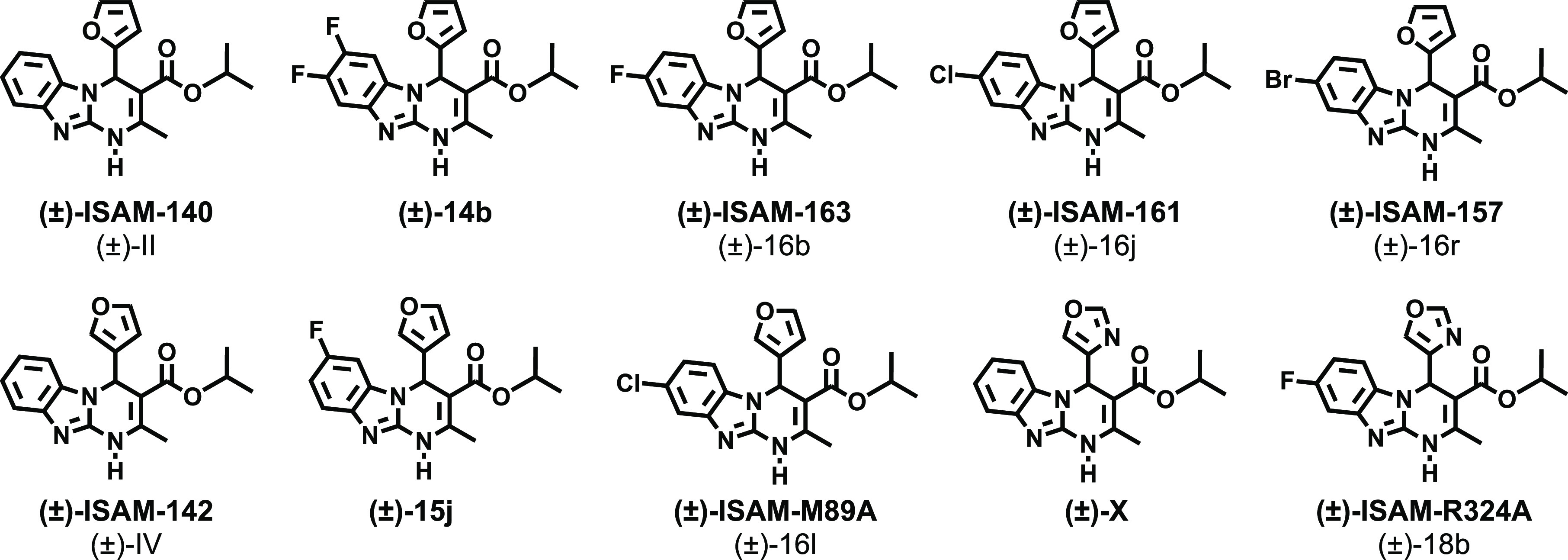
Preliminary ADME and Binding Data of Novel and Previously Reported A_2B_AR Antagonists

		microsomal stability			CYP3A4 IC_50_ (μM)^e^ or % inh. at 10 μM^f^		
compound	solubility (μM)^a^	percent^b^	*t*_1/2_^c^	Cl_int_^d^	DBF	7-BFC	hA_2B_*K*_*i*_ (nM)
**II** (**ISAM-140**)^30^	**25.2**				**2.90 ± 0.37**		**3.49 ± 0.2**
**14b**	62.0	20.97	28.93	21.65	>10 (39%)	>10 (58%)	11.9 ± 1.1
**16b** (**ISAM-163**)	47.5	19.83	27.40	20.07	>10 (40%)	>10 (63%)	3.05 ± 0.7
**16j** (**ISAM-161**)	23.7	22.75	28.14	18.60	3.68 ± 0.47	>10 (54%)	5.03 ± 0.3
**16r** (**ISAM-157**)	70.7	32.13	36.79	14.23	2.26 ± 0.30	>10 (52%)	5.23 ± 0.4
**IV** (**ISAM-142**)^30^	**26.1**				**2.68 ± 0.53**		**11.4 ± 0.5**
**15j**	58.2	16.83	21.13	26.44	>10 (47%)	>10 (11%)	44.3 ± 1.4
**16l** (**ISAM-M89A**)	21.9	41.02	44.80	11.68	6.36 ± 1.02	>10 (44%)	6.10 ± 0.7 (A_2B_) 176 ± 4 (A_2A_)
**X**(31)	**39.7**				**5.92 ± 0.68**		**43.4 ± 1.3**
**18b** (**ISAM-R324A**)	64.6	35.15	40.8	12.83	>10 (31%)	>10 (52%)	6.10 ± 0.3
**PSB-603**(28)	0.2						0.553
**progesterone**	6.7						
**prazosin**	31.3						
**testosterone**		7.11	16.13	32.44			
**ketoconazole**					0.008 μM	0.027 μM	

a Solubility in 1:99 DMSO/PSB buffer.

b Percentage remanent (sampling time 60 min).

c *t*_1/2_ in min.

d Intrinsic clearance in microliters per minute per milligram of protein.

e IC_50_ value obtained by extrapolation with analysis software.

f Due the low activity shown at CYP3A4, the percentage of inhibition at 10 μΜ is reported.

All ligands evaluated exhibit excellent solubility (21–70 μM) in PBS at pH 7, especially if compared to the solubility reported for a potent xanthine-based antagonist (PSB-603, Table 8).^28^ The introduction of fluorine atoms (7,8-difluoro, 7-fluoro, and 8-fluoro) improves up to twofold the solubility measured for the non-halogenated parent compounds. This trend also holds for the compound bearing an oxazole ring at position 4 (**18b**). Chlorination at position 8 does not improve or slightly decreases solubility (see data for **16j** and **16l**), whereas bromination greatly increases solubility [e.g., **16r** (70.7 μM) vs **ISAM-140** (25.2 μM)]. Liver microsome assay data (Table 8) revealed that the selected A_2B_AR antagonists showed moderate stability, with 16–41% of the compound remaining after 60 min of exposure to human microsomes and half-life ranging from 21 to 45 min. These data suggest that microsomal stability directly correlates with halogen size [e.g., **16b** (F), **16j** (Cl), and **16r** (Br) with 19.83, 22.75, and 32.13% remanent at 60 min, respectively]. These findings are currently guiding an ongoing study to improve the microsomal stability of these promising A_2B_AR antagonists.

To identify potential metabolic liabilities within the series here documented, the selected A_2B_AR antagonists were tested in vitro for CYP3A4 inhibitory activity. CYP3A isoforms are ubiquitous in the liver, have a broad substrate specificity,^48^ and are essential for the clearance of xenobiotics in humans (e.g., being involved in the metabolization of more than 50% of prescribed drugs). All experiments were performed in duplicate using fluorescence detection, employing ketoconazole as a reference compound (Table 8). The results of this study reveal IC_50_ values lower than 10 μM for all fluorinated compounds toward CYP3A4 (with inhibitory activity ranging from 31 to 40% at 10 μM), improving in all cases the measured CYP3A4 activity as compared to their non-halogenated congeners. A comparative analysis suggests that for 7-halo derivatives (Table 8, Cmpds **16b**, **16j**, and **16r**), increasing the size of the halogen increases the interaction with CYP3A4 (Table 8). Although the preliminary CYP3A4 data for three ligands seem to be suboptimal, we should keep in mind that the differences between the *h*A_2B_AR *K*_*i*_ of all compounds and the CYP3A4 IC_50_ (more than 200-fold in the worst case) makes CYP3A4 blockade not substantially important at the expected therapeutic doses. A more systematic study (e.g., including other relevant CYP subfamilies) would be required to draw definitive conclusions. In summary, an integrated analysis of pharmacodynamic studies and ADMET data allowed the identification of several new halogenated A_2B_AR antagonists that combine excellent affinity and selectivity and improved the ADMET profile.

### In Vitro Evaluation of the Immunostimulatory Effect

**Figure 10:**
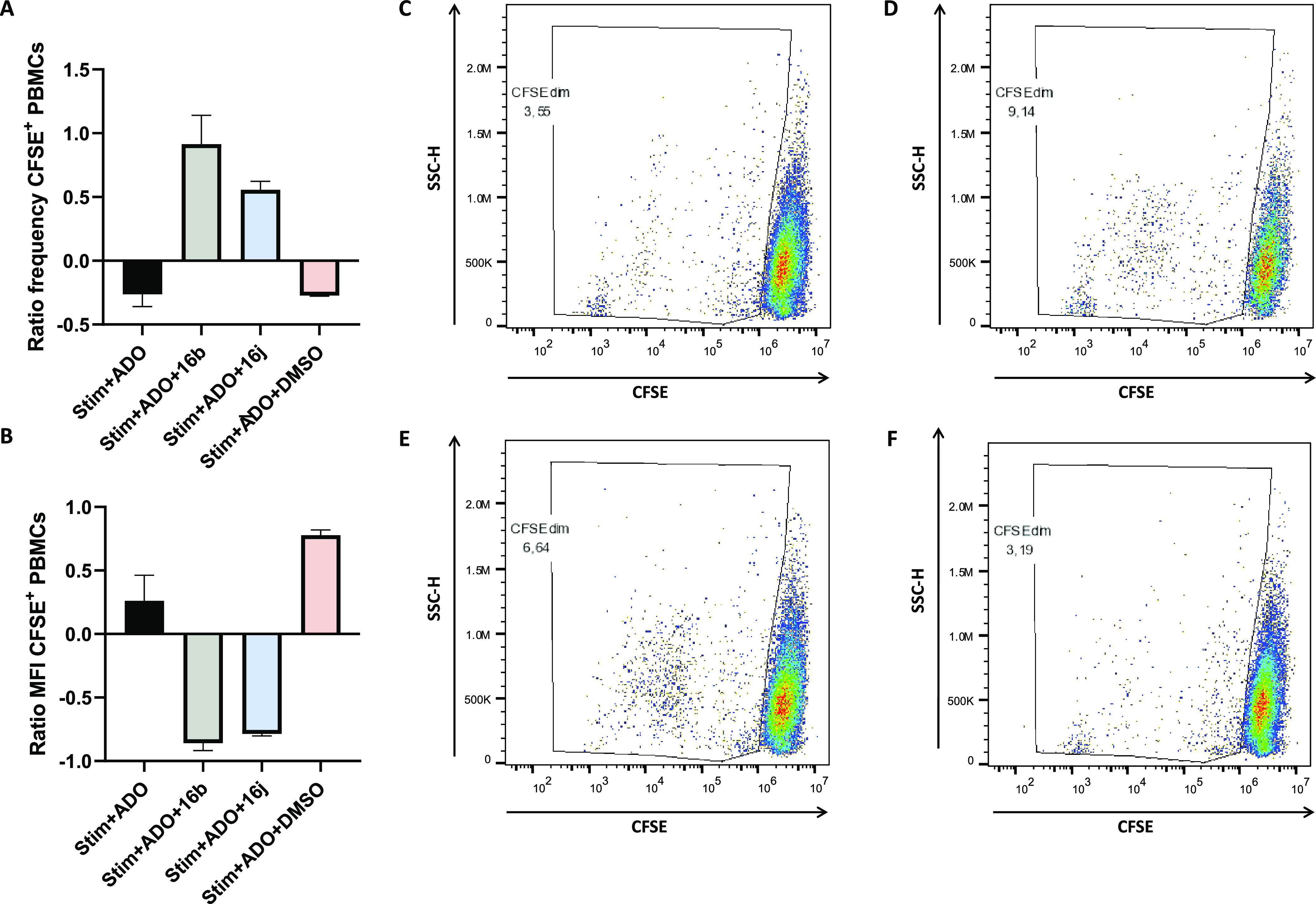
Effect of compounds **16b** and **16j** on the proliferation of PBMCs stimulated with antiCD3/CD28 + IL-2 in the presence of adenosine (ADO). PBMCs were CFSE-labeled, stimulated with anti-CD3/CD28 antibodies and IL-2, and cultured with the compounds of interest in the presence of adenosine for 3 days. The frequency of proliferating cells (CFSE^dim^) as well as their median fluorescence intensities were quantified and ratios calculated against the values of stimulated PBMCs with no further treatment. (A) Frequency of proliferating cells. (B) MFI of proliferating cells. (C–F) Representative dots plots of the proliferation of CFSE-stained PBMCs treated with anti-CD3/CD28 + IL-2 + ADO (C), anti-CD3/CD28 + IL-2 + ADO +16b (D), anti-CD3/CD28 + IL-2 + ADO +16j (E), and anti-CD3/CD28 + IL-2 + ADO + DMSO (F).

Ado has proved to be a potent immunosuppressive metabolite that suppresses the functions of multiple types of immune cells. To gain evidence supporting the role of the here optimized A_2B_AR antagonists in the context of cancer immunotherapy, it was decided to evaluate the effect of two representative compounds (**16b** and **16j**) in the proliferative activity of a prototypical immune cell. The human peripheral blood mononuclear cells (PBMCs) were selected for the study; they are critical components of the immune system and are involved in both humoral and cell-mediated immunity. Ado-mediated blockade of PBMC proliferation is well documented and characterized;^51,52^ we therefore investigated whether our A_2B_AR antagonists (**16b** and **16j**) can reverse the blockade of ADO-mediated proliferation in human PBMCs. PBMCs, isolated from a healthy donor, were labeled with carboxyfluorescein succinimidyl ester (CFSE) and then activated by adding anti-CD3/CD28 antibodies and interleukin 2 (IL-2). CFSE is a cell dye that allows for the determination of cell proliferation, as dividing cells progressively lose fluorescence. We found that, as expected, the addition of Ado caused a reduction in the frequency of PBMCs dividing cells (Figure 10A) and a corresponding increase of CFSE median fluorescence intensity (MFI) (Figure 10B) compared to the stimulated PBMCs. Notably, compounds **16b** and **16j** unequivocally reversed the Ado-induced blocking effect, resulting in an enhancement in the frequency of proliferative cells (Figure 10A,C–F) that correlates with a marked decrease in CFSE MFI (Figure 10B). From a quantitative point of view, the effect measured for compound **16b** (9.14%) is superior to that observed for **16j** (6.54%) (Figure 10D,E). Control experiments verified that the vehicle used to dissolve the compounds (DMSO) has a similar profile to that shown by ADO, thereby demonstrating that it does not favor PBMC proliferation by itself (Figure 10A–C,F).

These initial results confirm the effect of the A_2B_AR antagonists optimized here at the immune system level and their potential in the context of cancer immunotherapy. Further studies are ongoing in our laboratories to elucidate and obtain a comprehensive view of the immunological signature of A_2B_AR antagonists.

## Conclusions

A large collection of pyrimidine-based A_2B_AR antagonists was obtained and evaluated as part of our program aimed to unravel the therapeutic potential of blocking A_2B_AR in the context of cancer (immuno)therapy. Eight new A_2B_AR antagonists, combining notable affinity (*K*_*i*_ < 10 nM) and attractive subtype selectivity, were identified during the assessment of the impact of (di- and mono-)halogenation at positions 7 and 8 on the A_2B_AR affinity in a series of alkyl 7/8-halo-4-heteroaryl-1,4-dihydro-benzo[4,5]-imidazo[1,2-*a*]pyrimidine-3-carboxylates. The study showed that introduction of two halogen atoms (at positions 7 and 8) and the halogenation at position 7 produced an important, halogen-size-dependent, decay in A_2B_AR affinity. In clear contrast, halogenation at position 8 produced potent A_2B_AR ligands irrespective of the nature of the halogen. The SAR trends observed here were substantiated by a structure-based molecular modeling study including FEP simulations. The project also provided additional evidence supporting the importance of the stereodisposition of the pentagonal ring at position 4 on the A_2B_AR affinity, with some eutomers eliciting sub-nanomolar A_2B_AR affinity and retaining the remarkable subtype selectivity. As part of this study was identified the first example of a dual A_2A_/A_2B_AR antagonist exhibiting a stereoselective recognition at A_2A_AR and A_2B_AR. The functional profile of representative ligands as A_2B_AR antagonists was confirmed by cAMP-based assays. Evaluation of different ADME parameters enabled to verify the benefits of the halogenation but also to identify lead compounds combining improved affinity and selectivity and a good PK profile. Finally, two ligands optimized here unequivocally reversed the Ado-mediated antiproliferative effect on human PBMCs, thus highlighting their potential in the context of cancer immunotherapy.

## Experimental Section

### Chemistry

All starting materials, reagents, and solvents were purchased and used without further purification. After extraction from aqueous phases, the organic solvents were dried over anhydrous magnesium sulfate. The reactions were monitored by TLC on 2.5 mm Merck silica gel GF 254 strips, and the purified compounds each showed a single spot. Unless stated otherwise, UV light and/or iodine vapor were used to detect compounds. The Biginelli reactions were performed in coated Kimble vials on a PLS (6 × 4) Organic Synthesizer with orbital stirring or Anton Paar Microwave Synthesis Reactor. The purity and identity of all tested compounds were established by a combination of HPLC, mass spectrometry, and NMR spectroscopy as described in the Supporting Information. Purification of isolated products was carried out by column chromatography (Kieselgel 0.040–0.063 mm, E. Merck) or medium-pressure liquid chromatography (MPLC) on a Combi Flash Companion (Teledyne ISCO) with RediSep pre-packed normal-phase silica gel (35–60 μm) columns followed by recrystallization. Melting points were determined on a Stuart Scientific melting point apparatus and are uncorrected. The NMR spectra were recorded on Bruker AM300 and XM500 spectrometers. Chemical shifts are given as δ values against tetramethylsilane as internal standard, and *J* values are given in Hertz. Mass spectra were obtained on a Varian MAT 711 instrument. For regioisomer differentiation experiments, ^1^H NMR, 2D-ROESY, and 2D-NOESY were recorded on an AV NEO 750 MHz instrument. High-resolution mass spectra were obtained on an AutoSpec Micromass spectrometer. Analytical HPLC was performed on a Water Breeze 2 system (binary pump 1525, detector UV/Visible 2489, 7725i Manual Injector Kit 1500 Series) using a Luna 5 μm Silica (2) 100 Å, LC Column 150 × 4.6 mm column with gradient elution using the mobile phases dichloromethane, isopropanol, and a flow rate of 1 mL/min. The purity of all tested compounds was determined to be >95%.

The chiral resolution was performed using a Water Breeze 2 (binary pump 1525, detector UV/Visible 2489, 7725i Manual Injector Kit 1500 Series). Compound **16b** enantiomers were separated using a 250 mm × 10 mm Lux 5 μm Amylose-2 (Phenomenex) while **ISAM-140**, **16j**, **16l**, **16r**, **16s**, and **18c** enantiomers were separated using a 250 mm × 20 mm CHIRALPAK 5 μm IE-3 (DAICEL). A detailed description of the experimental protocols and relevant parameters (retention times, stereochemical purities) is provided in the Supporting Information. All single stereoisomers were isolated and their stereochemical purity analyzed by chiral HPLC (>97% for each stereoisomer) and then characterized by NMR in CDCl_3_. CD spectra were recorded on a Jasco-815 system equipped with a Peltier-type thermostatic accessory (CDF-426S, Jasco). Measurements were carried out at 20 °C using a 1 mm quartz cell in a volume of 300–350 mL. Compounds (0.1 mg) were dissolved in MeOH (1.0 mL). The instrument settings were bandwidth, 1.0 nm; data pitch, 1.0 nm; speed, 500 nm/min; accumulation, 10; and wavelengths, 400–190 nm. A detailed description of the synthesis and structural and spectroscopic data obtained for all compounds described is provided in the Supporting Information.

#### General Procedure for the Synthesis of Alkyl 7,8-dihalobenzo[4,5]imidazo[1,2-*a*]pyrimidine-3-carboxylates **14a**–**x**

(Method A): A mixture of the corresponding 5,6-dihalo-2-aminobenzimidazole **11a–c** (1.5 equiv), aldehyde **12a–d** (1 equiv), β-ketoester **13a**,**b** (1 equiv), and ZnCl_2_ (0.1 equiv) in THF (2.5 mL) was stirred with orbital stirring at 90 °C for 12 h. After completion of the reaction, as indicated by TLC, the solvent was removed in vacuum and the obtained oily residue was purified by column chromatography on silica gel, obtaining the corresponding alkyl 7,8-dihalobenzo[4,5]imidazo[1,2-*a*]pyrimidine-3-carboxylates (**14a–x**).

#### General Procedure for the Synthesis of Alkyl 7-halobenzo[4,5]imidazo[1,2-*a*]pyrimidine-3-carboxylates **15a**–**x** and 8-Halobenzo[4,5]imidazo[1,2-*a*]pyrimidine-3-carboxylates **16a**–**x**

(Method A): A mixture of 2-amino-5-halobenzimidazole **11d–f** (1.5 equiv), aldehyde **12a–d** (1 equiv), *β*-ketoester **13a**,**b** (1 equiv), and ZnCl_2_ (0.1 equiv) in THF (2.5 mL) was stirred with orbital stirring at 90 °C for 12 h. After completion of the reaction, as indicated by TLC, the solvent was removed in vacuum and the obtained oily residue was purified by column chromatography on silica gel, obtaining the corresponding alkyl 7- and 8-halobenzo[4,5]imidazo[1,2-*a*]pyrimidine-3-carboxylates.

#### General Procedure for the Synthesis of Alkyl 7-Halo-4-(oxazol-4-yl)-benzo[4,5]imidazo[1,2-*a*]pyrimidine-3-carboxylates **17a**–**d** and 8-Halo-4-(oxazol-4-yl)-benzo[4,5]imidazo[1,2-*a*]pyrimidine-3-carboxylates **18a**–**d**

(Method B): A mixture of 2-amino-5-halobenzimidazole **11d**,**e** (1.5 equiv), oxazole-4-carbaldehyde **12e** (1 equiv), *β*-ketoester **13a**,**b** (1 equiv), and AcOH (three drops) in THF (2 mL) and DMF (1 mL) was stirred under microwave irradiation at 80 °C for 90 min. After completion of the reaction, as indicated by TLC, the solvent was removed in vacuum and the obtained oily residue was purified by column chromatography on silica gel, obtaining the corresponding alkyl 7- and 8-halo-4-(oxazol-4-yl)-benzo[4,5]imidazo[1,2-*a*]pyrimidine-3-carboxylates (**17a–d** and **18a–d**).

### Pharmacology Binding Assays

Radioligand binding competition assays were performed in vitro using human ARs expressed in transfected HeLa [*h*A_2A_AR (9 pmol/mg protein) and *h*A_3_AR (3 pmol/mg protein)], HEK-293 [*h*A_2B_AR (1.5 pmol/mg protein)], and CHO [*h*A_1_AR (1.5 pmol/mg protein)] cells as described previously.^25,31,33^ A brief description is given below. A_1_AR competition binding experiments were carried out in membranes from CHO-A_1_ cells labeled with 1 nM [^3^H]DPCPX (*K*_D_ = 0.7 nM). Non-specific binding was determined in the presence of 10 μM R-PIA. The reaction mixture was incubated at 25 °C for 60 min. A_2A_AR competition binding experiments were carried out in membranes from HeLa-A_2A_ cells labeled with 3 nM [^3^H]ZM241385 (*K*_D_ = 2 nM). Non-specific binding was determined in the presence of 50 μM NECA. The reaction mixture was incubated at 25 °C for 30 min. A_2B_AR competition binding experiments were carried out in membranes from HEK-293-A_2B_ cells (Euroscreen, Gosselies, Belgium) labeled with 25 nM [^3^H]DPCPX (*K*_D_ = 21 nM). Non-specific binding was determined in the presence of 400 μM NECA. The reaction mixture was incubated at 25 °C for 30 min. A_3_AR competition binding experiments were carried out in membranes from HeLa-A_3_ cells labeled with 10 nM [^3^H]NECA (*K*_D_ = 8.7 nM). Non-specific binding was determined in the presence of 100 μM R-PIA. The reaction mixture was incubated at 25 °C for 180 min. After the incubation time, membranes were washed and filtered and radioactivity was detected in a MicroBeta TriLux reader (PerkinElmer).

### Pharmacology Functional Experiments

#### Reagents

The following reagents were used: adenosine deaminase (ADA, Roche Diagnostics, Mannheim, Germany), 5′-*N*-ethylcarboxamidoadenosine (NECA, Tocris, Bristol, UK), zardaverine (Calbiochem, San Diego, California, USA), SNAP-Surface Alexa Fluor 488 (New England BioLabs, Ipswich, Massachusetts, USA).

##### Cloning

The cDNA encoding the human A_2B_AR was amplified by polymerase chain reaction using the primers FA2BREcoRV (5′-tcgag**GATATC**CTGCTGGAGACACAGGACGC-3′) and RA2BRHindIII (5′-cgag**AAGCTT**TCATAGGCCCACACCGAGAGC-3′) and subcloned into the *Eco*RV/*Hind*III restriction sites of the pRK5-A_1_R^SNAP^ vector (kindly provided by Dr. J. P. Pin, Université de Montpellier and Institut de Génomique Fonctionnelle, Montpellier, France) by replacing the Ado A_1_ receptor. The resulting construct encoded the human A_2B_AR tagged with SNAP, a 20 kDa mutant of the DNA repair protein O^6^-alkylguanine-DNA alkyltransferase (AGT), at its N-terminus (pRK5-A_2B_AR^SNAP^).

##### Cell Culture and Stable Transfection

Human embryonic kidney (HEK)-293 T cells were grown in Dulbecco’s modified Eagle’s medium (DMEM) (Sigma-Aldrich, St. Louis, Missouri, USA) supplemented with 1 mM sodium pyruvate (Biowest, Nuaillé, France), 2 mM l-glutamine (Biowest), 100 U/mL streptomycin (Biowest), 100 mg/mL penicillin (Biowest), and 5% (v/v) fetal bovine serum (Invitrogen, Carlsbad, California, USA) at 37 °C and in an atmosphere of 5% CO_2_. HEK-293 cells growing in 60 cm^2^ plates were transfected with pRK5-A_2B_AR^SNAP^ using the polyethylenimine (PEI) method.^53^ After 48 h of transfection, cells were stained using SNAP-Surface Alexa Fluor 488.^38^ Fluorescent cells were selected every 2 weeks for 2 months using a Cell sorter (MoFlo Astrios, Beckman Coulter) to enrich the percentage of cells that express the receptor, thus ensuring its permanent expression.^54^

##### cAMP Accumulation Assay

cAMP accumulation was measured using the LANCE Ultra cAMP kit (PerkinElmer, Waltham, Massachusetts, USA) as previously described.^55^ In brief, HEK-293 T cells permanently expressing the A_2B_AR^SNAP^ construct (A_2B_AR^SNAP^ cells) were detached with Accutase (Sigma-Aldrich) and incubated for 1 h at 22 °C in Dulbecco’s modified Eagle’s medium (DMEM) (Sigma-Aldrich) supplemented with 0.1% BSA, ADA (0.5 U/mL), and zardaverine (100 μM). A_2B_AR^SNAP^ cells (1 × 10^4^ cells/200 μL) were incubated with NECA in the presence/absence of increasing concentrations of **16b**, **16j**, **16r**, and **16s**, during 30 min at 22 °C. Eu-cAMP tracer and ULight-anti-cAMP reagents were prepared and added to the sample following the manufacturer’s instructions. The 384-well plate was incubated 1 h at 22 °C in the dark and was then read on a CLARIOstar Microplate Reader (BMG Labtech, Durham, North Carolina, USA). Measurements at 620 and 665 nm were used to detect the TR-FRET signal, and the concomitant cAMP levels were calculated following the manufacturer’s instructions. Data were fitted by non-linear regression using GraphPad Prism 9 (San Diego, California, USA).

Concentration–response curves were carried out by assaying different **16b**, **16j**, **16r**, and **16s** concentrations ranging between 10 nM and 30 μM. Data was expressed as *K*_B_ by following the formula reported by Leff and Dougall (eq 1):^56^
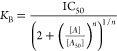
1

Correction offset value for all the ABFE estimates.^56^

where IC_50_ is the concentration of compound that inhibits NECA effect by 50%, [*A*] is the concentration of NECA employed in the assay, [*A*_50_] is the NECA EC_50_ value, and *n* is the Hill slope of the curve.

### Cellular Impedance Label-Free Assay

The xCELLigence RTCA system (Roche) was employed to assess the impact of blocking A_2B_AR-mediated effects in cellular impedance upon receptor activation, as previously described.^57−60^ To this end, A_2B_AR^SNAP^ cells were growth in 16-well E-plates (Roche), using DMEM supplemented with 1 mM sodium pyruvate, 2 mM l-glutamine, 100 U/mL streptomycin, 100 mg/mL penicillin, and 1.5% (v/v) fetal bovine serum in the presence of 0.5 U/mL of ADA. Of note, wells were previously coated with 50 μL fibronectin (10 μg/mL; Sigma-Aldrich) and the background index for each well was determined with supplemented DMEM (90 μL) in the absence of cells. Subsequently, A_2B_AR^SNAP^ cells (90 μL) were plated at a cell density of 10,000 cells/well and grown for 18 h in the RTCA SP device station (Roche) at 37 °C in an atmosphere of 5% CO_2_. Then, before ligand addition, cell index values were normalized to the same time point using the RTCA software, providing the normalized cell index (NCI). After ligand stimulation, NCI was recorded every 15 s for a total time of at least 45 min. The area under the curve (AUC) for every condition was calculated using GraphPad Prism 9.

### Data and Statistical Analysis

Data are represented as mean ± standard error of mean (SEM) with statistical significance set at *P* < 0.05. The number of samples (*n*) in each experimental condition is indicated in the corresponding figure legend. Outliers were assessed by the ROUT method;^61^ thus, any sample was excluded assuming a *Q* value of 1% in GraphPad Prism 9 (San Diego, California, USA). Comparisons among experimental groups were performed by the Student *t* test or one-way analysis of variance (ANOVA) followed by Tukey’s multiple-comparison post hoc test using GraphPad Prism 9, as indicated.

### CYP3A4 Inhibition

The inhibitory activity of selected compounds was evaluated by following a previously described method.^31^ Incubations were conducted in a 200 μL volume in 96-well microtiter plates (Costar 3915). Addition of a cofactor–buffer mixture (KH_2_PO_4_ buffer, 1.3 mM NADP, 3.3 mM MgCl_2_, 3.3 mM glucose-6-phosphate, and 0.4 U/mL glucose-6-phosphate dehydrogenase), supersome control, standard inhibitor (ketoconazole from Sigma-Aldrich) previously diluted, and compounds to plates were carried out by a liquid handling station (Zephyr Caliper). The plate was then pre-incubated at 37 °C for 5 min and the reaction initiated by the addition of a pre-warmed enzyme/substrate (E/S) mix. The E/S mix contained buffer (KH_2_PO_4_), c-DNA-expressed P450 in insect cell microsomes, substrate (DBF: dibenzylfluorescein or 7-BFC: 7-benzyloxy-4-trifluoromethyl-coumarin) and other components to give the final assay concentrations in a reaction volume of 200 μL. Reactions were terminated after various times (a specific time for each cytochrome) by addition of STOP solution (ACN/Tris–HCl 0.5 M 80:20, and NaOH 2 N for CYP3A4). Fluorescence per well was measured using a fluorescence plate reader (Tecan Infinite M1000 PRO), and percentage of inhibition was calculated. In silico predictions were performed using free web-based models (FAME 3, SmartCYP, and Xenosite).^62−64^

### Human Microsomal Stability

The human microsomal stability of selected compounds was evaluated by following a previously described method.^31^ The human microsomes employed were purchased from Tebu-Xenotech. The compound was incubated with the microsomes at 37 °C in a 50 mM phosphate buffer (pH = 7.4) containing 3 mM MgCl_2_, 1 mM NADP, 10 mM glucose-6-phosphate, and 1 U/mL glucose-6-phosphate-dehydrogenase. Samples (75 μL) were taken from each well at 0, 10, 20, 40, and 60 min and transferred to a plate containing 4 °C 75 μL acetonitrile and 30 μL of 0.5% formic acid in water was added for improving the chromatographic conditions. The plate was centrifuged (46,000 g, 30 min), and supernatants were taken and analyzed in a UPLC-MS/MS (Xevo TQD, Waters) by employing a BEH C18 column and an isocratic gradient of 0.1% formic acid in water:0.1% formic acid acetonitrile (60:40). The metabolic stability of the compounds was calculated from the logarithm of the remaining compounds at each of the time point studied.

### Solubility Determinations

The solubility of selected compounds was evaluated by following a previously described method.^31^ A 10 mM stock solution of the compound was serially diluted in 100% DMSO, and 2.5 μL of this solution was added to a 384-well UV-transparent plate (Greiner) containing 47.5 μL of PBS (pH = 7). The plate was incubated at 37 °C for 4 h, and the light scattering was measured in a NEPHELOstar Plus reader (BMG LABTECH). The data was fitted to a segmented linear regression for measuring the compound solubility.

### System Preparation and MD/FEP Simulations

The structural model for the A_2B_AR receptor derives from a homology model previously reported^31,33^ and here processed using the Protein Preparation Wizard pipeline in Maestro (Schrodinger ver. 2021-3). Subsequently, possible 3D tautomers and protomers at pH 7 ± 2 were generated for each compound using the OPLS4 force field^65^ and Epik, the lowest energy conformer was chosen for molecular docking. A receptor grid was generated with the default Van der Waals radius scaling settings, and it was positioned in the center of geometry of the binding site. Thereafter, Glide SP docking was performed leading to the protein–ligand complexes, which were used as the starting point for the MD/FEP simulations carried out with the software package Q.^66^ An FEP network was generated for each of chemical series using ECFP4 as a measure of the chemical similarity between ligand pairs, ensuring smooth alchemical transformations while covering the entire dataset. For each vertex comparing a pair of compounds part of the FEP network, the QligFEP^67^ pipeline was used for generating the MD/FEP input files and its posterior analysis. The MD simulations were carried out under spherical boundary conditions (SBC) with a sphere size of 25 Å, with solvent atoms lying in the outer shell of the sphere (22–25 Å) subject to radial and polarization restrains using the surface-constrained all-atom solvent (SCAAS).^68,69^ All titratable residues outside the sphere were neutralized, and histidine protonation states were set by the Protein Preparation Wizard. Atoms outside the simulation sphere were excluded from the calculation of the nonbonded interactions and tightly constrained (200 kcal/mol·Å^2^) to its position. Long-range electrostatic interactions beyond a 10 Å threshold were evaluated using the local reaction field method,^68^ excluding the atoms undergoing the FEP transformation where no cut-off is applied. The OPLS-AA/M force field^70^ was used for the protein and solvent (TIP3P) parameters, while OPLS2005 ligand parameters were generated using the ffld_server;^71^ solvent bonds and angles were constrained using the SHAKE algorithm.^72^ The MD/FEP simulations are preceded by a heating phase of the sphere from 0.1 to 298 K during a short 31 ps stage where a positional restraint of 10 kcal/mol·Å^2^ is progressively released. This is followed by a 100 ps unbiased and unrestrained equilibration, and subsequently, MD sampling is performed across a predefined sigmoidal λ schedule, consisting of 101 λ windows, each consisting of 10 ps sampling using a 2 fs timestep. To get initial relative binding free energy estimates (RBFE), the thermodynamic cycles are closed for each ligand pair defined in the FEP network by performing the corresponding MD/FEP simulation in a water sphere, and the free energies are calculated with the Bennet acceptance ratio (BAR) method.^66^ The cycle closure correction framework^73^ was utilized for assessing the convergence of the initial RBFE estimates, and additionally, it yielded corrected RBFE values, which eliminate the hysteresis along all the thermodynamic cycles encompassed by the FEP network. To get the ABFE for all the compounds of interest, the corrected RBFE values together with the FEP networks and the experimental binding free energy for a reference compound per network are used. The Bellman–Ford implementation from NetworkX^74^ was used for finding the shortest path connecting a reference with the remaining target compounds, and, for each target compound, eq 1 was used to get the ABFE estimates. Finally, a correction offset value for all the ABFE estimates was calculated as described by Wang et al.^75^ in order to minimize the experimental error introduced to the final estimates (eq 2).
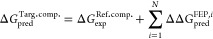
2

Correction offset value for all the ABFE estimates.^75^

### In Vitro Evaluation of the Immunostimulatory Effect

PBMCs were isolated from a healthy donor, stained with CFSE (Abcam) following the manufacturer’s protocol, and cultured in duplicates in U-bottom 96-well plates at a density of 1.5 × 10^5^ cells/well in RPMI 1640 medium (Gibco) supplemented with 10% AB Human Serum (Sigma-Aldrich) and 1% penicillin–streptomycin (Gibco). PBMCs were stimulated with ImmunoCult Human CD3/CD28 T Cell Activator (STEMCELL Technologies) and 50 U/mL Interleukin 2 (Gibco). Ado was then added at a final concentration of 0.1 mM and compounds **16b** and **16j** at 15 μM. DMSO was used as vehicle control. PBMCs were kept in culture with the different treatments for 3 days in an incubator at 37 °C and 5% CO_2_. After the culture period, cells were washed twice with DPBS (Gibco) and CFSE fluorescence measured in a BD Accuri flow cytometer. The data was analyzed using FlowJo software (BD): the percentage of CFSE^+^ cells, and their median fluorescence intensity (MFI) was quantified for each treatment condition, and a ratio against the stimulated condition without Ado was calculated for each treatment. Positive values indicate increases in the variable compared to the stimulated control, while negative values indicate decreases in the measured variable.

## Supporting Information Available

Molecular formula strings (CSV)
Atomic coordinates and experimental data upon article publication (ZIP)
Spectroscopic and analytical data for all compounds described, HPLC enantiomeric separations, X-ray crystallography assays, supplementary figures, supplementary tables, and HPLC traces for lead compounds (PDF)

## Abbreviations

ABFE: absolute binding free energies
ADMET: absorption, distribution, metabolism, excretion, and toxicity
Ado: adenosine
AGT: O^6^-alkylguanine-DNA alkyltransferase
AR: adenosine receptor
AUC: area under curve
cAMP: cyclic adenosine monophosphate
CD: circular dichroism
CFSE: carboxyfluorescein succinimidyl ester
CHO: Chinese hamster ovary
Cmpd: compound
Cmpds: compounds
DMEM: Dulbecco’s modified Eagle’s medium
DMF: dimethylformamide
DPCPX: dipropylcyclopentyl-xanthine
FEP: free energy perturbation
GPCRs: G protein-coupled receptors
MD: molecular dynamics
MFI: median fluorescence intensities
MPLC: medium pressure liquid chromatography
NECA: *N*-ethoxycarbonyl adenosine
NMR: nuclear magnetic resonance
P_1_: class 1 purinergic receptors
PAINS: pan-assay interference compounds
PBMCs: peripheral blood mononuclear cells
R-PIA: *R*-phenylisopropyl-adenosine
RBFE: relative binding free energies
ROESY: rotating frame Overhauser effect spectroscopy
SAR: structure–activity relationships
SBC: spherical boundary conditions
SCAAS: surface-constrained all-atom solvent
SSR: structure–selectivity relationships
THF: tetrahydrofuran
Veh: vehicle.
