# Supplementary material for: Exploring the Effect of Halogenation in a Series of Potent and Selective A2B Adenosine Receptor Antagonists
Source: J Med Chem. 2022 Dec 14;66(1):890–912. doi: 10.1021/acs.jmedchem.2c01768 (PMC9841532; doi:10.1021/acs.jmedchem.2c01768)
Supplement: Supplementary file 3 — jm2c01768_si_003.pdf [file jm2c01768_si_003.pdf]

SUPPORTING INFORMATION

Exploring the Effect of Halogenation in a Series of Potent and Selective A_{2B} Adenosine Receptor Antagonists

Rubén Prieto-Díaz,[1,2,3] Manuel González-Gómez,[1,2] Hugo Fojo-Carballo,[1,2] Jhonny Azuaje,[1,2] Abdelaziz El Maatougui,[1,2] Maria Majellaro,[1,2] María I. Loza,[4,5] José Brea,[4,5*] Víctor Fernández-Dueñas,[7,8] M. Rita Paleo,[1,2] Alejandro Díaz-Holguín,[3] Beatriz Garcia-Pinel,[4,6] Ana Mallo-Abreu,[1,2] Juan C. Estévez,[1,2] Antonio Andújar-Arias,[1,2] Xerardo García-Mera,[2] Iria Gomez-Tourino,[4] Francisco Ciruela,[7,8] Cristian O. Salas,[9] Hugo Gutiérrez-de-Terán[3*] and Eddy Sotelo[1,2*]

[1]Center for Research in Biological Chemistry and Molecular Materials (CIQUS), University of Santiago de Compostela, 15782 Santiago de Compostela, Spain. [2]Department of Organic Chemistry, Faculty of Pharmacy, University of Santiago de Compostela, 15782 Santiago de Compostela, Spain. [3]Department of Cell and Molecular Biology, Uppsala University, Biomedical Center, 75124 Uppsala, Sweden. [4]Center for Research in Metabolic Diseases (CIMUS). University of Santiago de Compostela, 15782 Santiago de Compostela, Spain. [5]Department of Pharmacology, Pharmacy and Pharmaceutical Technology. Faculty of Pharmacy, University of Santiago de Compostela, 15782 Santiago de Compostela, Spain. [6]Pharmacology Unit, Department of Pathology and Experimental Therapeutics, Faculty of Medicine and Health Sciences, Institute of Neuroscience, University of Barcelona, 08907 L'Hospitalet de Llobregat, Spain. [7]Neuropharmacology and Pain Group, Neuroscience Program, Institut d'Investigació Biomèdica de Bellvitge, IDIBELL, 08907 L'Hospitalet de Llobregat, Spain. [8]Departamento de Química Orgánica, Facultad de Química y de Farmacia, Pontificia Universidad Católica de Chile, Vicuña Mackenna 4860, Macul, Santiago 7820436, Chile.

*To whom correspondence should be addressed:
(HGDT): +46 18 471 5056, Fax.: +46 18 536971, e-mail: hugo.gutierrez@icm.uu.se
(ES) +34 881815732, Fax.: +34-881815704, e-mail: e.sotelo@usc.es
(JB) +34 881815459, Fax.: +34-8818115474, e-mail: pepo.brea@usc.es

TABLE OF CONTENTS

Chemistry. General information....S2
Spectroscopic and analytical data for all compounds described....S3
HPLC enantiomeric separation and characterization of **ISAM-163**, **ISAM-161**, **ISAM-M89A**, **ISAM-157**, **ISAM-M114A** and **ISAM-R316A**....S26
X-ray crystallography of compound **ISAM-M114A**....S45
Supplementary Figures and Tables....S47
HPLC traces for lead compounds....S56

Chemistry. General information

Unless otherwise indicated, all starting materials, reagents and solvents were purchased and used without further purification. After extraction from aqueous phases, the organic solvents were dried over anhydrous magnesium sulfate. The reactions were monitored by thin-layer chromatography (TLC) on 2.5 mm Merck silica gel GF 254 strips, and the purified compounds each showed a single spot; unless stated otherwise, UV light and/or iodine vapor were used to detect compounds. The Biginelli reactions were performed in coated Kimble vials on a PLS (6×4) Organic Synthesizer with orbital stirring or Anton Paar Microwave Synthesis Reactor in the indicated reactions. The purity and identity of all tested compounds were established by a combination of HPLC, mass spectrometry and NMR spectroscopy as described in the supporting information. Purification of isolated products was carried out by column chromatography (Kieselgel 0.040–0.063 mm, E. Merck) or medium pressure liquid chromatography (MPLC) on a Combi Flash Companion (Teledyne ISCO) with RediSep pre-packed normal-phase silica gel (35–60 µm) columns followed by recrystallization. Melting points were determined on a Stuart Scientific melting point apparatus and are uncorrected. The NMR spectra were recorded on Bruker AM300 and XM500 spectrometers. Chemical shifts are given as δ values against tetramethylsilane as internal standard and *J* values are given in Hz. Mass spectra were obtained on a Varian MAT-711 instrument. For regioisomer differentiation experiments, HSQC, HMBC, COSY, NOESY and band-2D NOESY were recorded on AV NEO 750 MHz instrument. High-resolution mass spectra were obtained on an Autospec Micromass spectrometer. Analytical HPLC was performed on a Water Breeze™ 2 system (binary pump 1525, detector UV/Visible 2489, 7725i Manual Injector Kit 1500 Series) using a Luna 5 µm Silica (2) 100 Å, LC Column 150 x 4.6 mm column with gradient elution using the mobile phases dichloromethane, isopropanol, and a flow rate of 1 ml/min. The purity of all tested compounds was determined to be >95%. A detailed description of synthetic methodologies as well as analytical and spectroscopic data for all described compounds is described below.

Spectroscopic and analytical data for all compounds described

(±) Ethyl 7,8-difluoro-4-(furan-2-yl)-2-methyl-1,4-dihydrobenzo[4,5]imidazo[1,2-*a*]pyrimidine-3-carboxylate (14a). Purified by column chromatography (*n*-Hexane–Ethyl Acetate 3:1 – 1:2) to give 89 mg, 31%. Mp: 268 – 269 ºC. ^{1}H NMR (300 MHz, DMSO-d_6), δ (ppm): 10.80 (brs, 1H, NH), 7.62 – 7.50 (m, 1H, Ar-H), 7.47 – 7.30 (m, 2H, Ar-H), 6.57 (s, 1H, CH), 6.47 (d, J = 3.1 Hz, 1H, Ar-H), 6.35-6.27 (m, 1H, Ar-H), 4.19 – 3.94 (m, 2H, OCH_2CH$_3$), 2.43 (s, 3H, CH$_3$) 1.15 (t, J = 7.1 Hz, 3H, OCH$_2$CH_3). ^{13}C NMR (75 MHz, DMSO-d_6) δ (ppm): 170.1, 157.3, 152.7 (d, J = 245.1 Hz), 150.3 (d, J = 245.0 Hz), 148.9, 143.1, 132.3, 115.6, 113.3, 109.9, 104.1, 103.8, 99.7, 70.1, 64.6, 54.4, 23.8, 19.3. HRMS (APCI) *m/z* calcd for $C_{18}H_{16}F_2N_3O_3$ [M+H]$^+$: 360.1081; found: 360.1155.

(±) Isopropyl 7,8-difluoro-4-(furan-2-yl)-2-methyl-1,4-dihydrobenzo[4,5]imidazo[1,2-*a*] pyrimidine-3-carboxylate (14b). Purified by column chromatography (*n*-Hexane–Ethyl Acetate 3:1 – 1:2) to give 114 mg, 38% Mp: 261 – 262 ºC. ^{1}H NMR (300 MHz, DMSO-d_6), δ (ppm): 10.90 (brs, 1H, NH), 7.61-7.55 (m, 1H, Ar-H), 7.44-7.37 (m, 2H, Ar-H), 6.54-6.51 (m, 2H, Ar-H), 6.32 (s, 1H, CH), 4.90-4.82 (m, 1H, OCH(CH$_3$)$_2$), 2.42 (s, 3H, CH$_3$), 1.21 (d, J = 6.5 Hz, 3H, OCH(CH_3)$_2$), 1,04 (d, J = 6.4 Hz, 3H, OCH(CH_3)$_2$). ^{13}C NMR (75 MHz, CDCl$_3$), δ (ppm): 165.0, 152.9 (d, J = 245.0), 147.5 (d, J = 245.0), 146.6, 142.3, 136.3, 127.1, 110.5, 108.0, 104.3, 104.1, 98.7, 98.4, 96.0, 67.6, 50.2, 22.1, 21.7, 19.2. HRMS (APCI) *m/z*: calcd for $C_{19}H_{18}F_2N_3O_3$ [M+H]$^+$: 374.1238; found: 374.1273.

(±) Ethyl 7,8-difluoro-4-(furan-3-yl)-2-methyl-1,4-dihydrobenzo[4,5]imidazo[1,2-*a*]pyrimidine-3-carboxylate (14c). Purified by column chromatography (*n*-Hexane–Ethyl Acetate 3:1 – 1:2) to give 77 mg, 27%. Mp: 275 – 277 ºC. ^{1}H NMR (300 MHz, DMSO-d_6), δ (ppm): 11.53 (brs, 1H, NH), 7.74 (s, 1H, Ar-H), 7.65 – 7.46 (m, 1H, Ar-H), 7.45 (s, 1H, Ar-H), 7.44 – 7.35 (m, 1H, Ar-H), 6.45 (s, 1H, CH), 6.19 (s, 1H, Ar-H), 4.14 – 3.98 (m, 2H), 2.40 (s, 3H, CH$_3$), 1.16 (t, J = 7.2, 3H, OCH$_2$CH_3). ^{13}C NMR (75 MHz, CDCl$_3$), δ (ppm): 165.6, 148.0, 146.3, 145.7, 143.7, 139.6, 136.5, 125.2, 108.6, 104.6, 104.3, 98.5, 98.4, 98.1, 60.4, 48.6, 19.4, 14.4. HRMS (APCI) *m/z* calcd for $C_{18}H_{16}F_2N_3O_3$ [M+H]$^+$: 360.1081; found: 360.1156.

(±) Isopropyl 7,8-difluoro-4-(furan-3-yl)-2-methyl-1,4-dihydrobenzo[4,5]imidazo[1,2-*a*] pyrimidine-3-carboxylate (14d). Purified by column chromatography (*n*-Hexane–Ethyl Acetate 3:1 – 1:2) to give 91 mg, 31%. Mp: 237 – 238 ºC. ^{1}H NMR (300 MHz, DMSO-d_6), δ (ppm): 10.75 (brs, 1H, NH), 7.76 (s, 1H, Ar-H), 7.66 – 7.59 (m, 1H, Ar-H), 7.45 (s, 1H, Ar-H), 7.40 – 7.33 (m, 1H, Ar-H), 6.43 (s, 1H, Ar-H), 6.21 (s, 1H, CH), 4.88 (m, 1H, OC*H*(CH_3)$_2$), 2.40 (s, 3H, CH_3), 1.22 (d, *J* = 6.1 Hz, 3H, OCH(CH_3)$_2$), 1,08 (d, *J* = 6.0 Hz, 3H, OCH(CH_3)$_2$). ^{13}C NMR (75 MHz, $CDCl_3$), δ (ppm): 165.2, 148.1, 146.3, 145.5, 143.7, 139.7, 136.6, 126.8, 125.2, 108.7, 104.6, 104.3, 98.7, 98.1, 67.9, 48.7, 22.2, 21.9, 19.4. LRMS (CI) *m/z* calcd for $C_{19}H_{18}F_2N_3O_3$ $[M+H]^+$: 374.4; found: 374.1.

(±) Ethyl 7,8-difluoro-2-methyl-4-(thiophen-2-yl)-1,4-dihydrobenzo[4,5]imidazo[1,2-*a*]pyrimidine-3-carboxylate (14e). Purified by column chromatography (*n*-Hexane–Ethyl Acetate 3:1 – 1:2) to give 86 mg, 29%. Mp: 272 – 273 ºC. ^{1}H NMR (300 MHz, DMSO-d_6), δ (ppm): 10.94 (brs, 1H, NH), 7.71 – 7.63 (m, 1H, Ar-H), 7.41 – 7.30 (m, 2H, Ar-H), 7.16 – 7.14 (m, 1H, Ar-H), 6.88 – 6.85 (m, 1H, Ar-H), 6.82 (s, 1H, CH), 4.12 – 4.00 (m, 2H, OC$H_2$$CH_3$), 2.42 (s, 3H, CH_3), 1.15 (t, *J* = 7.0, 3H, OCH_2CH_3). LRMS (CI) *m/z* calcd for $C_{18}H_{16}F_2N_3O_2S$ $[M+H]^+$: 376.4; found: 376.1.

(±) Isopropyl 7,8-difluoro-2-methyl-4-(thiophen-2-yl)-1,4-dihydrobenzo[4,5]imidazo[1,2-*a*] pyrimidine-3-carboxylate (14f). Purified by column chromatography (*n*-Hexane–Ethyl Acetate 3:1 – 1:2) to give 103 mg, 34%. Mp: 263 – 264 ºC. ^{1}H NMR (300 MHz, DMSO-d_6) δ (ppm): 10.9 (brs, 1H, NH), 7.76 – 7.72 (m, 1H, Ar-H), 7.41 – 7.35 (m, 1H, Ar-H), 7.32 (d, *J* = 5.5 Hz, 1H, Ar-H), 7.19 (d, *J* = 3.5 Hz, 1H, Ar-H), 6.92 – 6.77 (m, 1H, Ar-H), 6.79 (s, 1H, CH), 4.88 (m, 1H, OC*H*(CH_3)$_2$), 2.42 (s, 3H, CH_3), 1.22 (d, *J* = 6.3 Hz, 3H, OCH(CH_3)$_2$), 1.05 (d, *J* = 6.3 Hz, 3H, OCH(CH_3)$_2$). LRMS (CI) *m/z* calcd for $C_{19}H_{18}F_2N_3O_2S$ $[M+H]^+$: 390.4; found: 390.2.

(±) Ethyl 7,8-difluoro-2-methyl-4-(thiophen-3-yl)-1,4-dihydrobenzo[4,5]imidazo[1,2-*a*]pyrimidine-3-carboxylate (14g). Purified by column chromatography (*n*-Hexane–Ethyl Acetate 3:1 – 1:2) to give 88 mg, 29%. Mp: 278 – 280 ºC. ^{1}H NMR (300 MHz, DMSO-d_6), δ (ppm): 10.83 (brs, 1H, NH), 7.61 – 7.53 (m, 2H, Ar-H), 7.40 – 7.33 (m, 2H, Ar-H), 6.90 – 6.88 (m, 1H, Ar-H), 6.55 (s, 1H, CH), 4.10 – 3.98 (m, 2H, OC$H_2$$CH_3$), 2.48 (s, 3H, CH_3), 1.14 (t, *J* = 7.2, 3H, OCH_2CH_3). LRMS (CI) *m/z* calcd for $C_{18}H_{16}F_2N_3O_2S$ $[M+H]^+$: 376.4; found: 376.1.

(±) Isopropyl 7,8-difluoro-2-methyl-4-(thiophen-3-yl)-1,4-dihydrobenzo[4,5]imidazo-[1,2-*a*] pyrimidine-3-carboxylate (14h). Purified by column chromatography (*n*-Hexane–Ethyl Acetate 3:1 – 1:1) to give 85 mg, 28 %. Mp: 264 – 265 ºC. ^{1}H NMR (300 MHz, DMSO-d_6), δ (ppm): 10.82 (brs, 1H, NH), 7.63 – 7.57 (m, 2H, Ar-H), 7.40 – 7.33 (m, 2H, Ar-H), 6.91 – 6.89 (m, 1H, Ar-H), 6.52 (s, 1H, CH), 4.85 – 4.78 (m, 1H, OC*H*(CH_3)$_2$), 2.41 (s, 3H, CH_3), 1.21 (d, J = 6.1 Hz, 3H, OCH(CH_3)$_2$), 1.01 (d, J = 6.4 Hz, 3H, OCH(CH_3)$_2$). LRMS (CI) *m/z* calcd for $C_{19}H_{18}F_2N_3O_2S$ [M+H]$^+$: 390.4; found: 390.1.

(±) Ethyl 7,8-dichloro-4-(furan-2-yl)-2-methyl-1,4-dihydrobenzo[4,5]imidazo[1,2-*a*]pyrimidine-3-carboxylate (14i). Purified by column chromatography (*n*-Hexane–Ethyl Acetate 3:1 – 1:1) to give 123 mg, 48%. Mp: 285 – 286 ºC. ^{1}H NMR (300 MHz, DMSO-d_6) δ (ppm): 10.98 (s, 1H, NH), 7.76 (s, 1H, Ar-H), 7.58 (s, 1H, Ar-H), 7.49 – 7.42 (m, 1H, Ar-H), 6.64 (s, 1H, CH), 6.51 (d, J = 3.2 Hz, 1H, Ar-H), 6.32 (dd, J = 3.3, 1.8 Hz, 1H, Ar-H), 4.17 – 3.93 (m, 2H, OCH_2CH$_3$), 2.43 (s, 3H, CH_3), 1.15 (t, J = 7.1 Hz, 3H, OCH$_2$CH_3). LRMS (CI) *m/z* calcd for $C_{18}H_{16}Cl_2N_3O_3$[M+H]$^+$: 393.2; found: 393.0.

(±) Isopropyl 7,8-dichloro-4-(furan-2-yl)-2-methyl-1,4-dihydrobenzo[4,5]imidazo[1,2-*a*] pyrimidine-3-carboxylate (14j). Purified by column chromatography (*n*-Hexane–Ethyl Acetate 3:1 – 1:1) to give 110 mg, 39%. Mp: 299 – 300 ºC. ^{1}H NMR (300 MHz, DMSO-d_6), δ (ppm): 10.95 (brs, 1H, NH), 7.76 (s, 1H, Ar-H), 7.58 (s, 1H, Ar-H), 7.46 (s, 1H, Ar-H), 6.61 (s, 1H, Ar-H), 6.53 (s, 1H, CH), 6.34 – 6.32 (m, 1H, Ar-H), 4.92 – 4.83 (m, 1H, OC*H*(CH_3)$_2$), 2.43 (s, 3H, CH_3), 1.22 (d, J = 6.1 Hz, 3H, OCH(CH_3)$_2$), 1.05 (d, J = 6.4 Hz, 3H, OCH(CH_3)$_2$). ^{13}C NMR (75 MHz, DMSO-d_6), δ (ppm): 164.8, 152.5, 148.0, 147.5, 143.3, 142.7, 131.8, 124.9, 122.6, 118.2, 111.8, 110.9, 108.8, 95.7, 67.2, 49.8, 22.2, 21.9, 18.9. LRMS (CI) *m/z* calcd for $C_{19}H_{18}Cl_2N_3O_3$ [M+H]$^+$: 407.3; found: 406.1.

(±) Ethyl 7,8-dichloro-4-(furan-3-yl)-2-methyl-1,4-dihydrobenzo[4,5]imidazo[1,2-*a*]pyrimidine-3-carboxylate (14k). Purified by column chromatography (*n*-Hexane–Ethyl Acetate 3:1 – 1:1) to give 120 mg, 46%. Mp: 267 – 269 ºC. ^{1}H NMR (300 MHz, $CDCl_3$), δ (ppm): 10.89 (brs, 1H, NH), 7.78 (s, 1H, Ar-H), 7.76 (s, 1H, Ar-H), 7.76 (s, 1H, Ar-H), 7.57 (s, 1H, Ar-H), 7.46 (s, 1H, Ar-H), 6.51 (s, 1H, CH), 4.12 – 4.04 (m, 2H, OCH_2CH$_3$), 2.40 (s, 3H, CH_3), 1.18 (t, J = 7.0 Hz, 3H, OCH$_2$CH_3). ^{13}C NMR (75 MHz, DMSO-d_6), δ (ppm): 165.4, 155.5, 148.2, 145.5, 143.8, 139.7, 133.74, 130.8, 126.7, 124.9, 117.6, 110.9, 108.5, 99.1, 60.4, 48.6, 25.3, 14.3. LRMS (CI) *m/z* calcd for $C_{18}H_{16}Cl_2N_3O_3$ [M+H]$^+$: 392.1; found: 392.1.

(±) Isopropyl 7,8-dichloro-4-(furan-3-yl)-2-methyl-1,4-dihydrobenzo[4,5]imidazo[1,2-*a*] pyrimidine-3-carboxylate (14l). Purified by column chromatography (*n*-Hexane–Ethyl Acetate 3:1 – 1:1) to give 88 mg, 31%. Mp: 253 – 255 ºC. ^{1}H NMR (300 MHz, $CDCl_3$), δ (ppm): 11.18 (brs, 1H, NH), 7.56 (s, 1H, Ar-H), 7.25 – 7.18 (m, 3H, Ar-H), 6.43 (s, 1H, CH), 6.21 (s, 1H, Ar-H), 5.05 – 4.96 (m, 1H, OC*H*(CH_3)$_2$), 2.58 (s, 3H, CH_3), 1.28 (d, *J* = 6.0 Hz, 3H, OCH(CH_3)$_2$), 1,22 (d, *J* = 5.9 Hz, 3H, OCH(CH_3)$_2$). LRMS (CI) *m/z* calcd for $C_{19}H_{18}Cl_2N_3O_3$ [M+H]$^+$: 407.3; found: 407.3.

(±) Ethyl 7,8-dichloro-2-methyl-4-(thiophen-2-yl)-1,4-dihydrobenzo[4,5]imidazo[1,2-*a*]pyrimidine-3-carboxylate (14m). Purified by column chromatography (*n*-Hexane–Ethyl Acetate 3:1 – 1:1) to give 124 mg, 46%. Mp: 279 – 280 ºC. ^{1}H NMR (300 MHz, $CDCl_3$) δ (ppm): 10.9 (brs, 1H, NH), 7.76 – 7.72 (m, 1H, Ar-H), 7.38 (s, 1H, Ar-H), 7.25 (s, 1H, Ar-H), 6.96 – 6.90 (m, 1H, Ar-H), 6.93 – 6.85 (m, 1H, Ar-H), 6.70 (s, 1H, CH), 4.31 – 4.26 (m, 2H, OC$H_2$$CH_3$), 2.67 (s, 3H, CH_3), 1.29 (t, *J* = 7.1 Hz, 3H, OCH_2CH_3). LRMS (CI) *m/z* calcd for $C_{18}H_{16}Cl_2N_3O_2S$ [M+H]$^+$: 409.3; found: 409.3.

(±) Isopropyl 7,8-dichloro-2-methyl-4-(thiophen-2-yl)-1,4-dihydrobenzo[4,5]imi-dazo[1,2-*a*]pyrimidine-3-carboxylate (14n). Purified by column chromatography (*n*-Hexane–Ethyl Acetate 3:1 – 1:1) to give 78 mg, 28%. Mp: 281 – 283 ºC. ^{1}H NMR (300 MHz, $CDCl_3$) δ (ppm): 11.22 (brs, 1H, NH), 7.38 (s, 1H, Ar-H), 7.29 – 7.06 (m, 2H, Ar-H), 6.98 – 6.91 (m, 1H, Ar-H), 6.45 (s, 1H, CH), 5.12 – 4.98 (m, 1H, OC*H*(CH_3)$_2$), 2.65 (s, 3H, CH_3), 1.28 (d, *J* = 6.0 Hz, 3H, OCH(CH_3)$_2$), 1,21 (d, *J* = 5.9 Hz, 3H, OCH(CH_3)$_2$). LRMS (CI) *m/z* calcd for $C_{19}H_{18}Cl_2N_3O_2S$ [M+H]$^+$: 422.0; found: 422.0.

(±) Ethyl 7,8-dichloro-2-methyl-4-(thiophen-3-yl)-1,4-dihydrobenzo[4,5]imidazo[1,2-*a*]pyrimidine-3-carboxylate (14o). Purified by column chromatography (*n*-Hexane–Ethyl Acetate 3:1 – 1:1) to give 96 mg, 36%. Mp: 279 – 281 ºC. ^{1}H NMR (300 MHz, $CDCl_3$) δ (ppm): 11.19 (brs, 1H, NH), 7.38 (s, 1H, Ar-H), 7.29 (s, 1H, Ar-H), 7.21 – 7.16 (m, 1H, Ar-H), 6.95 – 6.92 (m, 1H, Ar-H), 6.92 – 6.84 (m, 1H, Ar-H), 6.70 (s, 1H, CH), 4.31 – 4.26 (m, 2H, OC$H_2$$CH_3$), 2.65 (s, 3H, CH_3), 1.25 (t, *J* = 7.0 Hz, 3H, OCH_2CH_3). LRMS (CI) *m/z* calcd for $C_{18}H_{16}Cl_2N_3O_2S$ [M+H]$^+$: 409.0; found: 409.0.

(±) Isopropyl 7,8-dichloro-2-methyl-4-(thiophen-3-yl)-1,4-dihydrobenzo[4,5]imidazo-[1,2-*a*] pyrimidine-3-carboxylate (14p). Purified by column chromatography (*n*-Hexane–Ethyl Acetate 3:1 – 1:1) to give 115 mg, 41%. Mp: 285 – 286 ºC. ^{1}H NMR (300 MHz, $CDCl_3$), δ (ppm): 11.05 (brs, 1H, NH), 7.49 (s, 1H, Ar-H), 7.18 – 7.13 (m, 3H, Ar-H), 6.92 – 6.83 (m, 1H, Ar-H), 6.55 (s, 1H, CH), 5.18 – 5.02 (m, 1H, OC*H*(CH_3)$_2$), 2.62 (s, 3H, CH_3), 1.30 (d, J = 6.0 Hz, 3H, OCH(C*H*$_3$)$_2$), 1.21 (d, J = 6.1 Hz, 3H, OCH(C*H*$_3$)$_2$). ^{13}C NMR (75 MHz, $CDCl_3$), δ (ppm): 165.0, 145.0, 141.1, 126.7, 125.9, 124.7, 122.9, 117.6, 110.9, 99.9, 67.9, 52.2, 22.2, 21.8, 19.4. LRMS (CI) *m/z* calcd for $C_{19}H_{18}Cl_2N_3O_2S$ [M+H]$^+$: 422.0; found: 422.0.

(±) Ethyl 7,8-dibromo-4-(furan-2-yl)-2-methyl-1,4-dihydrobenzo[4,5]imidazo[1,2-*a*]pyrimidine-3-carboxylate (14q). Purified by column chromatography (*n*-Hexane–Ethyl Acetate 2:1 – 1:2) to give 84 mg, 38%. Mp: ≥ 300 ºC dec. ^{1}H NMR (300 MHz, DMSO-d_6) δ (ppm): 10.98 (s, 1H, NH), 7.89 (s, 1H, Ar-H), 7.71 (d, J = 1.2 Hz, 1H, Ar-H), 7.45 (d, J = 1.6 Hz, 1H, Ar-H), 6.65 (s, 1H, CH), 6.50 (d, J = 3.3 Hz, 1H, Ar-H), 6.33 (dt, J = 3.2, 1.5 Hz, 1H, Ar-H), 4.14 – 3.94 (m, 2H, OC*H*$_2$CH$_3$), 2.42 (s, 3H, CH_3), 1.15 (t, J = 7.1, 3H, OCH$_2$C*H*$_3$). LRMS (CI) *m/z* calcd for $C_{18}H_{16}Br_2N_3O_3$ [M+H]$^+$: 479.9; found: 479.9.

(±) Isopropyl 7,8-dibromo-4-(furan-2-yl)-2-methyl-1,4-dihydrobenzo[4,5]imidazo[1,2-*a*] pyrimidine-3-carboxylate (14r). Purified by column chromatography (*n*-Hexane–Ethyl Acetate 2:1 – 1:2) to give 113 mg, 50 %. Mp: 273 – 275 ºC. ^{1}H NMR (300 MHz, DMSO-d_6) δ (ppm): 10.95 (s, 1H, NH), 7.89 (s, 1H, Ar-H), 7.71 (s, 1H, Ar-H), 7.50 – 7.42 (m, 1H, Ar-H), 6.62 (s, 1H, CH), 6.50 (t, J = 3.4 Hz, 1H, Ar-H), 6.33 (t, J = 2.5 Hz, 1H, Ar-H), 4.87 (p, J = 6.3 Hz, 1H, OC*H*(CH_3)$_2$), 2.42 (s, 3H, CH_3), 1.22 (d, J = 6.1 Hz, 3H, OCH(C*H*$_3$)$_2$), 1.05 (d, J = 6.0 Hz, 3H, OCH(C*H*$_3$)$_2$). ^{13}C NMR (75 MHz, $CDCl_3$), δ (ppm): 164.8, 152.4, 147.6, 146.4, 142.5, 141.4, 132.9, 120.6, 118.0, 115.9, 114.3, 110.5, 108.2, 96.7, 67.8, 50.2, 22.1, 21.8, 19.3. LRMS (CI) *m/z* calcd for $C_{19}H_{18}Br_2N_3O_3$ [M+H]$^+$: 493.9; found: 493.9.

(±) Ethyl 7,8-dibromo-4-(furan-3-yl)-2-methyl-1,4-dihydrobenzo[4,5]imidazo[1,2-*a*]pyrimidine-3-carboxylate (14s). Purified by column chromatography (*n*-Hexane–Ethyl Acetate 2:1 – 1:2) to give 103 mg, 47 %. Mp: 289 – 290 ºC. ^{1}H NMR (300 MHz, $CDCl_3$), δ (ppm): 11.17 (brs, 1H, NH), 7.68 (s, 1H, Ar-H), 7.42 (s, 1H, Ar-H), 7.37 (s, 1H, Ar-H), 7.28 – 7.22 (m, 1H, Ar-H), 6.38 (s, 1H, CH), 6.08 (s, 1H, Ar-H), 4.24 – 4.16 (m, 2H, OC*H*$_2$CH$_3$), 2.69 (s, 3H, CH_3), 1.28 – 1.18 (m, 3H, OCH$_2$C*H*$_3$). HRMS (APCI) *m/z* calcd for $C_{18}H_{16}Br_2N_3O_3$ [M+H]$^+$: 479.9553; found: 479.9552.

(±) Isopropyl 7,8-dibromo-4-(furan-3-yl)-2-methyl-1,4-dihydrobenzo[4,5]imidazo[1,2-*a*]pyrimidine-3-carboxylate (14t). Purified by column chromatography (*n*-Hexane–Ethyl Acetate 2:1 – 1:2) to give 112 mg, 49 %. Mp: 258 – 260 ºC. ^{1}H NMR (300 MHz, $CDCl_3$), δ (ppm): 11.38 (brs, 1H, NH), 7.69 (s, 1H, Ar-H), 7.44 – 7.39 (m, 1H, Ar-H), 7.33 (s, 1H, Ar-H), 7.31 – 7.26 (m, 1H, Ar-H), 6.41 (s, 1H, CH), 6.18 (s, 1H, Ar-H), 5.18 – 5.05 (m, 1H, OC*H*(CH_3)$_2$), 2.58 (s, 3H, CH_3), 1.37 (d, *J* = 6.1 Hz, 3H, OCH(C*H$_3$*)$_2$), 1.25 (d, *J* = 6.1 Hz, 3H, OCH(C*H$_3$*)$_2$). LRMS (CI) *m/z* calcd for $C_{19}H_{18}Br_2N_3O_3$ $[M+H]^+$: 494.0; found: 493.9.

(±) Ethyl 7,8-dibromo-2-methyl-4-(thiophen-2-yl)-1,4-dihydrobenzo[4,5]imidazo[1,2-*a*]pyrimidine-3-carboxylate (14u). Purified by column chromatography (*n*-Hexane–Ethyl Acetate 2:1 – 1:2) to give 109 mg, 48%. Mp: ≥ 300 ºC dec. ^{1}H NMR (300 MHz, $CDCl_3$), δ (ppm): 10.56 (brs, 1H, NH), 7.68 (s, 1H, Ar-H), 7.56 (s, 1H, Ar-H), 7.20 – 7.15 (m, 1H, Ar-H), 6.92 – 6.75 (m, 1H, Ar-H), 6.88 – 6.80 (m, 1H, Ar-H), 6.67 (s, 1H, CH), 4.24 – 4.00 (m, 2H, OC*H$_2$*CH_3), 2.64 (s, 3H, CH_3), 1.18 – 1.09 (m, 3H, OCH_2C*H$_3$*). LRMS (CI) *m/z* calcd for $C_{18}H_{16}Br_2N_3O_2S$ $[M+H]^+$: 495.9; found: 496.0.

(±) Isopropyl 7,8-dibromo-2-methyl-4-(thiophen-2-yl)-1,4-dihydrobenzo[4,5]imidazo-[1,2-*a*] pyrimidine-3-carboxylate (14v). Purified by column chromatography (*n*-Hexane–Ethyl Acetate 2:1 – 1:2) to give 97 mg, 41%. Mp: 280 – 282 ºC. ^{1}H NMR (300 MHz, $CDCl_3$), δ (ppm): 10.89 (brs, 1H, NH), 7.69 (s, 1H, Ar-H), 7.56 (s, 1H, Ar-H), 7.19 – 7.17 (m, 1H, Ar-H), 6,96 (s, 1H, Ar-H), 6.89 – 6.86 (m, 1H, Ar-H), 6.68 (s, 1H, CH), 5.14 – 5.05 (m, 1H, OC*H*(CH_3)$_2$), 2.65 (s, 3H, CH_3), 1.31 (d, *J* = 6.1 Hz, 3H, OCH(C*H$_3$*)$_2$), 1.20 (d, *J* = 6.2 Hz, 3H, OCH(C*H$_3$*)$_2$). LRMS (CI) *m/z* calcd for $C_{19}H_{18}Br_2N_3O_2S$ $[M+H]^+$: 509.9; found: 509.9.

(±) Ethyl 7,8-dibromo-2-methyl-4-(thiophen-3-yl)-1,4-dihydrobenzo[4,5]imidazo[1,2-*a*]pyrimidine-3-carboxylate (14w). Purified by column chromatography (*n*-Hexane–Ethyl Acetate 2:1 – 1:2) to give 78 mg, 34%. Mp: 288 – 290 ºC. ^{1}H NMR (300 MHz, DMSO-d_6) δ (ppm): 10.92 (brs, 1H, NH), 7.86 (s, 1H, Ar-H), 7.70 (s, 1H, Ar-H), 7.53 (dd, *J* = 3.0, 1.4 Hz, 1H, Ar-H), 7.37 (dd, *J* = 5.0, 3.0 Hz, 1H, Ar-H), 6.88 (d, *J* = 5.0 Hz, 1H, Ar-H), 6.62 (s, 1H, CH), 4.18 – 3.90 (m, 2H, OC*H$_2$*CH_3), 2.41 (s, 3H, CH_3), 1.16 (t, *J* = 7.0 Hz, 3H, OCH_2C*H$_3$*). LRMS (CI) *m/z* calcd for $C_{18}H_{16}Br_2N_3O_2S$ $[M+H]^+$: 495.9; found: 495.9.

(±) Isopropyl 7,8-dibromo-2-methyl-4-(thiophen-3-yl)-1,4-dihydrobenzo[4,5]imidazo-[1,2-*a*] pyrimidine-3-carboxylate (14x). Purified by column chromatography (*n*-Hexane–Ethyl Acetate 2:1 – 1:2) to give 120 mg, 51%. Mp: ≥300 dec. ^{1}H NMR (300 MHz, DMSO-d_6), δ (ppm): 10.65 (brs, 1H, NH), 7.78 (s, 1H, Ar-H), 7.40 (s, 1H, Ar-H), 7.32 – 7.15 (m, 2H, Ar-H), 6.92 – 6.85 (m, 1H, Ar-H), 6.49 (s, 1H, CH), 5.18 – 5.06 (m, 1H, OC*H*(CH$_3$)$_2$), 2.58 (s, 3H, CH$_3$), 1.32 (d, J = 6.0 Hz, 3H, OCH(C*H$_3$*)$_2$), 1.21 (d, J = 6.4 Hz, 3H, OCH(C*H$_3$*)$_2$). LRMS (CI) *m/z* calcd for $C_{19}H_{18}Br_2N_3O_2S$ [M+H]$^+$: 509.9; found: 509.9.

(±) Ethyl 7-fluoro-4-(furan-2-yl)-2-methyl-1,4-dihydrobenzo[4,5]imidazo[1,2-*a*]pyrimidine-3-carboxylate (15a). Purified by column chromatography (*n*-Hexane–Ethyl Acetate 3:1 – 1:2) to give 57 mg, 19%. Mp: 273 – 274 ºC. ^{1}H NMR (300 MHz, CDCl$_3$), δ (ppm): 7.45 – 7.35 (m, 1H, Ar-H), 7.32 – 7.28 (s, 2H, Ar-H), 7.27 – 7.10 (m, 1H, Ar-H), 6.51 (s, 1H, CH), 6.32 – 6.23 (m, 2H, Ar-H), 4.29 – 4.08 (m, 2H, OC*H$_2$*CH$_3$), 2.72 (s, 3H, CH$_3$), 1.28 (t, J = 7.1 Hz, 3H, OCH$_2$C*H$_3$*). LRMS (CI) *m/z* calcd for $C_{18}H_{17}FN_3O_3$ [M+H]$^+$: 342.1, found, 342.1.

(±) Isopropyl 7-fluoro-4-(furan-2-yl)-2-methyl-1,4-dihydrobenzo[4,5]imidazo[1,2-*a*]pyrimidine-3-carboxylate (15b). Purified by column chromatography (*n*-Hexane–Ethyl Acetate 3:1 – 1:2) to give 61 mg, 19%. Mp: 236 – 237 ºC. ^{1}H NMR (300 MHz, DMSO-d_6), δ (ppm): 10.84 (brs, 1H, NH), 7.43 – 7.38 (m, 2H, Ar-H), 7.16 – 7.12 (m, 1H, Ar-H), 6.89 – 6.82 (m, 1H, Ar-H), 6.54 (s, 1H, Ar-H), 6.49 (s, 1H, CH), 6.45 (s, 1H, Ar-H), 4.91 – 4.83 (m, 1H, OC*H*(CH$_3$)$_2$), 2.44 (s, 3H, CH$_3$), 1.21 (d, J = 6.1 Hz, 3H, OCH(C*H$_3$*)$_2$), 1.04 (d, J = 6.1 Hz, 3H, OCH(C*H$_3$*)$_2$). ^{13}C NMR (75 MHz, CDCl$_3$), δ (ppm): 165.2, 158.2, 153.2, 147.6, 146.8, 142.2, 141.8, 128.1, 110.0, 108.7, 107.8, 103.1, 102.7, 96.0, 67.5, 50.1, 22.1, 21.8, 19.3. HRMS (APCI) *m/z* calcd for $C_{19}H_{19}FN_3O_3$ [M+H]$^+$: 356.1332; found: 356.1369.

(±) Ethyl 7-fluoro-4-(furan-3-yl)-2-methyl-1,4-dihydrobenzo[4,5]imidazo[1,2-a]pyrimidine-3-carboxylate (15c). Purified by column chromatography (*n*-Hexane–Ethyl Acetate 3:1 – 1:2) to give 56 mg, 19%. Mp: 260 – 262 ºC. ^{1}H NMR (300 MHz, CDCl$_3$), δ (ppm): 7.45 – 7.17 (m, 3H, Ar-H), 6.91 – 6.80 (m, 1H, Ar-H), 6.51 (s, 1H, CH), 6.26 – 6.18 (m, 2H, Ar-H), 4.25 – 4.10 (m, 2H, OC*H$_2$*CH$_3$), 2.67 (s, 3H, CH$_3$), 1.32 (t, J = 7.1 Hz, 3H, OCH$_2$C*H$_3$*). LRMS (CI) *m/z* calcd for $C_{18}H_{17}FN_3O_3$ [M+H]$^+$: 342.1, found, 342.1.

(±) Isopropyl 7-fluoro-4-(furan-3-yl)-2-methyl-1,4-dihydrobenzo[4,5]imidazo[1,2-*a*]pyrimidine-3-carboxylate (15d). Purified by column chromatography (*n*-Hexane–Ethyl Acetate 3:1 – 1:2) to give 46 mg, 15%. Mp: 239 – 241 ºC. ^{1}H NMR (300 MHz, $CDCl_3$), δ (ppm): 7.41 – 7.35 (m, 1H, Ar-H), 7.25 – 7.05 (m, 2H, Ar-H), 6.98 – 6.68 (m, 2H, Ar-H), 6.48 (s, 1H, CH), 6.25 – 6.19 (m, 1H, Ar-H), 5.10 – 4.99 (m, 1H, $OCH(CH_3)_2$), 2.73 (s, 3H, CH_3), 1.32 (d, J = 6.1 Hz, 3H, $OCH(CH_3)_2$), 1.16 (d, J = 6.2 Hz, 3H, $OCH(CH_3)_2$). ^{13}C NMR (75 MHz, $CDCl_3$), δ (ppm): 165.1, 160.0, 156.9, 153.0, 146.9, 142.3, 137.1, 116.6, 116.5, 107.8, 97.6, 97.2, 95.7, 67.5, 50.1, 22.1, 21.8, 19.3. HRMS (APCI) *m/z*: calcd for $C_{19}H_{19}FN_3O_3$ $[M+H]^+$, 356.1332; found: 256.1362.

(±) Ethyl 7-fluoro-2-methyl-4-(thiophen-2-yl)-1,4-dihydrobenzo[4,5]imidazo[1,2-*a*]pyrimidine-3-carboxylate (15e). Purified by column chromatography (*n*-Hexane–Ethyl Acetate 3:1 – 1:2) to give 51 mg, 16%. Mp: 257 – 258 ºC. ^{1}H NMR (300 MHz, $CDCl_3$), δ (ppm): 7.41 – 7.36 (m, 1H, Ar-H), 7.18 – 7.12 (m, 1H, Ar-H), 7.02 – 6.84 (m, 4H, Ar-H), 6.72 (s, 1H, CH), 4.25 – 4.12 (m, 2H, OCH_2CH_3), 2.70 (s, 3H, CH_3), 1.28 (t, J = 6.9 Hz, 3H, OCH_2CH_3). LRMS (CI) *m/*z calcd for $C_{18}H_{17}FN_3O_2S$ $[M+H]^+$: 358.1; found: 358.1.

(±) Isopropyl 7-fluoro-2-methyl-4-(thiophen-2-yl)-1,4-dihydrobenzo[4,5]imidazo[1,2-*a*]pyrimidine-3-carboxylate (15f). Purified by column chromatography (*n*-Hexane–Ethyl Acetate 3:1 – 1:2) to give 44 mg, 13%. Mp: 257 – 258 ºC. ^{1}H NMR (300 MHz, $CDCl_3$), δ (ppm): 7.45 – 7.35 (m, 1H, Ar-H), 7.25 (d, J = 6.9 Hz, 1H, Ar-H), 7.14 – 7.09 (m, 1H, Ar-H), 7.02 – 6.95 (m, 3H, Ar-H), 6.68 (s, 1H, CH), 5.12 – 5.09 (m, 1H, $OCH(CH_3)_2$), 2.69 (s, 3H, CH_3), 1.33 (d, J = 6.2 Hz, 3H, $OCH(CH_3)_2$), 1.21 (d, J = 6.2 Hz, 3H, $OCH(CH_3)_2$). LRMS (CI) *m/z* calcd for $C_{19}H_{19}FN_3O_2S$ $[M+H]^+$: 372.1; found: 372.1.

(±) Ethyl 7-fluoro-2-methyl-4-(thiophen-3-yl)-1,4-dihydrobenzo[4,5]imidazo[1,2-*a*]pyrimidine-3-carboxylate (15g). Purified by column chromatography (*n*-Hexane–Ethyl Acetate 3:1 – 1:2) to give 55 mg, 17%. Mp: 271 – 273 ºC. ^{1}H NMR (300 MHz, $CDCl_3$), δ (ppm): 7.42 – 7.36 (m, 1H, Ar-H), 7.36 (s, 2H, Ar-H) 7.28 – 7.15 (m, 1H, Ar-H), 6.99 – 6.88 (m, 2H, Ar-H), 6.55 (s, 1H, CH), 4.26-4.05 (m, 2H, OCH_2CH_3), 2.45 (s, 3H, CH_3), 1.25 (t, J = 7.2 Hz, 3H, OCH_2CH_3). LRMS (CI) *m/*z calcd for $C_{18}H_{17}FN_3O_2S$ $[M+H]^+$: 358.1; found: 358.1.

(±) Isopropyl 7-fluoro-2-methyl-4-(thiophen-3-yl)-1,4-dihydrobenzo[4,5]imidazo[1,2-*a*]pyrimidine-3-carboxylate (15h). Purified by column chromatography (*n*-Hexane–Ethyl Acetate 3:1 – 1:2) to give 46 mg, 14%. Mp: 251 – 253 ºC. ^{1}H NMR (300 MHz, $CDCl_3$), δ (ppm): 11.85 (brs, 1H, NH), 7.39 – 7.05 (m, 4H, Ar-H), 6.85 – 6.72 (m, 2H, Ar-H), 6.59 – 6.49 (m, 1H, CH), 5.18 – 4.95 (m, 1H, $OC\mathit{H}(CH_3)_2$), 2.67 (s, 3H, CH_3), 1.30 (d, J = 5.8 Hz, 3H, $OCH(C\mathit{H_3})_2$), 1.20 (d, J = 6.0 Hz, 3H, $OCH(C\mathit{H_3})_2$). LRMS (CI) *m/z* calcd for $C_{19}H_{19}FN_3O_2S$ [M+H]$^+$: 372.1; found: 372.1.

(±) Ethyl 7-chloro-4-(furan-2-yl)-2-methyl-1,4-dihydrobenzo[4,5]imidazo[1,2-*a*]pyrimidine-3-carboxylate (15i). Purified by column chromatography (*n*-Hexane–Ethyl Acetate 3:1 – 1:1) to give 60 mg, 21%. Mp: 256 – 257 ºC. ^{1}H NMR (300 MHz, DMSO-d_6), δ (ppm): 7.41 – 7.36 (m, 1H, Ar-H), 7.29 – 7.26 (m, 2H, Ar-H), 7.19 – 7.11 (m, 1H, Ar-H), 6.53 – 6.47 (m, 1H, CH), 6.25 – 6.18 (m, 2H, Ar-H), 4.22 – 4.05 (m, 2H, $OC\mathit{H_2}CH_3$), 2.68 (s, 3H, CH_3), 1.22 (t, J = 7.0 Hz, 3H, $OCH_2C\mathit{H_3}$). ^{13}C NMR (75 MHz, DMSO-d_6), δ (ppm): 165.4, 152.8, 147.9, 147.3, 143.8, 143.2, 130.9, 126.8, 120.5, 116.8, 111.4, 110.9, 95.2, 59.9, 49.7, 40.6, 19.0, 14.6. LRMS (EI) (70eV) m/z calcd for $C_{18}H_{16}ClN_3O_3$ [M]$^+$: 357.1; found: 357.1.

(±) Isopropyl 7-chloro-4-(furan-2-yl)-2-methyl-1,4-dihydrobenzo[4,5]imidazo[1,2-*a*]pyrimidine-3-carboxylate (15j). Purified by column chromatography (*n*-Hexane–Ethyl Acetate 3:1 – 1:1) to give 62 mg, 21%. Mp: 232 – 234 ºC. ^{1}H NMR (300 MHz, $CDCl_3$), δ (ppm): 7.38 (s, 2H, Ar-H), 7.28 – 7.06 (m, 2H, Ar-H), 6.42 (s, 1H, CH), 6.13 (s, 2H, Ar-H), 5.09 – 4.98 (m, 1H, $OC\mathit{H}(CH_3)_2$), 2.65 (s, 3H, CH_3), 1.37 (d, J = 6.2 Hz, 3H, $OCH(C\mathit{H_3})_2$), 1.26 (d, J = 6.2 Hz, 3H, $OCH(C\mathit{H_3})_2$). ^{13}C NMR (75 MHz, $CDCl_3$), δ (ppm): 165.1, 153.0, 147.3, 146.8, 142.3, 141.8, 130.3, 128.3, 121.4, 116.2, 110.5, 110.4, 107.9, 96.3, 67.6, 50.1, 22.1, 21.8, 19.4. LRMS (EI) (70eV) *m/z* calcd for $C_{19}H_{18}ClN_3O_3$ [M]$^+$: 371.1; found: 371.1.

(±) Ethyl 7-chloro-4-(furan-3-yl)-2-methyl-1,4-dihydrobenzo[4,5]imidazo[1,2-*a*]pyrimidine-3-carboxylate (15k). Purified by column chromatography (*n*-Hexane–Ethyl Acetate 3:1 – 1:1) to give 53 mg, 19%. Mp: 236 – 237 ºC. ^{1}H NMR (300 MHz, DMSO-d_6) δ (ppm): 10.80 (s, 1H, NH), 7.70 (s, 1H, Ar-H), 7.48 – 7.34 (m, 2H, Ar-H), 7.01 (dd, J = 8.5, 2.0 Hz, 1H, Ar-H), 6.47 (s, 1H, CH), 6.22 – 6.14 (m, 1H, Ar-H), 4.19 – 3.96 (m, 2H, $OC\mathit{H_2}CH_3$), 2.41 (s, 3H, CH_3), 1.17 (t, J = 7.1 Hz, 3H, $OCH_2C\mathit{H_3}$).^{13}C NMR (75 MHz, $CDCl_3$), δ (ppm): 165.7, 147.9, 146.1, 143.5, 142.2, 139.6, 130.1, 128.3, 125.4, 121.3, 116.4, 110.2, 108.7, 98.6, 60.3, 48.5, 19.5, 14.4. LRMS (EI) (70eV) m/z calcd for $C_{18}H_{16}ClN_3O_3$ [M]$^+$: 357.1; found: 357.1.

(±) Isopropyl 7-chloro-4-(furan-3-yl)-2-methyl-1,4-dihydrobenzo[4,5]imidazo[1,2-*a*]pyrimidine-3-carboxylate (15l). Purified by column chromatography (*n*-Hexane–Ethyl Acetate 3:1 – 1:1) to give 50 mg, 17%. Mp: 241 – 243 ºC. ^{1}H NMR (300 MHz, $CDCl_3$), δ (ppm): 7.38 – 7.29 (m, 2H, Ar-H), 7.28 – 7.06 (m, 3H, Ar-H), 6.42 (s, 1H, CH), 6.15 – 6.09 (m, 1H, Ar-H), 5.14 – 4.99 (m, 1H, OC*H*(CH_3)$_2$), 2.65 (s, 3H, CH_3), 1.37 (d, *J* = 6.2 Hz, 3H, OCH(C*H*$_3$)$_2$), 1.26 (d, *J* = 6.2 Hz, 3H, OCH(C*H*$_3$)$_2$). ^{13}C NMR (75 MHz, $CDCl_3$), δ (ppm): 165.1, 147.6, 146.0, 143.6, 139.8, 139.7, 132.1, 126.6, 125.3, 123.1, 117.0, 109.7, 108.7, 98.8, 67.8, 48.4, 22.2, 21.9, 19.3. HRMS (APCI) *m/z* calcd. for $C_{19}H_{19}ClN_3O_3$ [M+H]$^+$: 372.1109; found: 372.1111.

(±) Ethyl 7-chloro-2-methyl-4-(thiophen-2-yl)-1,4-dihydrobenzo[4,5]imidazo[1,2-*a*]pyrimidine-3-carboxylate (15m). Purified by column chromatography (*n*-Hexane–Ethyl Acetate 3:1 – 1:1) to give 64 mg, 22%. Mp: 259 – 260 ºC. ^{1}H NMR (300 MHz, $CDCl_3$), *δ* (ppm): 7.44 – 7.38 (m, 1H, Ar-H), 7.30 – 7.22 (m, 1H, Ar-H), 7.21 – 7.16 (m, 1H, Ar-H), 6.96 – 6.91 (m, 1H, Ar-H), 6.99 – 6.86 (m, 1H, Ar-H), 6.85 – 6.82 (m, 1H, Ar-H), 6.72 (s, 1H, CH), 4.22 – 4.10 (m, 2H, OC*H*$_2$$CH_3$), 2.68 (s, 3H, CH_3), 1.37 (t, *J* = 7.1 Hz, 3H, OCH_2C*H*$_3$). LRMS (EI) (70eV) *m/z* calcd for $C_{18}H_{16}ClN_3O_2S$ [M]$^+$: 373.1; found: 372.9.

(±) Isopropyl 7-chloro-2-methyl-4-(thiophen-2-yl)-1,4-dihydrobenzo[4,5]imidazo[1,2-*a*]pyrimidine-3-carboxylate (15n). Purified by column chromatography (*n*-Hexane–Ethyl Acetate 3:1 – 1:1) to give 52 mg, 17%. Mp: 251 – 252 ºC. ^{1}H NMR (300 MHz, $CDCl_3$), *δ* (ppm): 11.79 (brs, 1H, NH), 7.38 – 7.08 (m, 3H, Ar-H), 6.99 – 6.93 (m, 1H, Ar-H), 6.90 – 6.84 (m, 1H, Ar-H), 6.75 (s, 1H, CH), 5.20 – 5.06 (m, 1H, OC*H*(CH_3)$_2$), 2.69 (s, 3H, CH_3), 1.36 (d, *J* = 6.0 Hz, 3H, OCH(C*H*$_3$)$_2$), 1.22 (d, *J* = 6.0 Hz, 3H, OCH(C*H*$_3$)$_2$). LRMS (EI) (70eV) *m/z* calcd for $C_{19}H_{18}ClN_3O_2S$ [M]$^+$: 387.1; found: 386.9.

(±) Ethyl 7-chloro-2-methyl-4-(thiophen-3-yl)-1,4-dihydrobenzo[4,5]imidazo[1,2-*a*]pyrimidine-3-carboxylate (15o). Purified by column chromatography (*n*-Hexane–Ethyl Acetate 3:1 – 1:1) to give 44 mg, 15%. Mp: 267 – 268 ºC. ^{1}H NMR (300 MHz, $CDCl_3$), *δ* (ppm): 11,78 (brs, 1H, NH), 7.42 – 7.04 (m, 5H, Ar-H), 6.96 – 6.89 (m, 1H, Ar-H), 6.57 (s, 1H, CH), 4.22 – 4.04 (m, 2H, OC*H*$_2$$CH_3$), 2.68 (s, 3H, CH_3), 1.31 – 1.29 (m, 3H, OCH_2C*H*$_3$). LRMS (CI) *m/z* calcd for $C_{18}H_{17}ClN_3O_2S$ [M+H]$^+$: 374.1; found: 374.1.

(±) Isopropyl 7-chloro-2-methyl-4-(thiophen-3-yl)-1,4-dihydrobenzo[4,5]imidazo[1,2-*a*]pyrimidine-3-carboxylate (15p). Purified by column chromatography (*n*-Hexane–Ethyl Acetate 3:1 – 1:1) to give 50 mg, 16%. Mp: 279 – 281 ºC. ^{1}H NMR (300 MHz, $CDCl_3$), δ (ppm): 11.68 (brs, 1H, NH), 7.38 – 7.35 (m, 1H, Ar-H), 7.21 – 6.99 (m, 4H, Ar-H), 6.82 (s, 1H, Ar-H), 6.51 (s, 1H, CH), 5.18 – 5.02 (m, 1H, $OCH(CH_3)_2$), 2.68 (s, 3H, CH_3), 1.30 (d, J = 6.0 Hz, 3H, $OCH(CH_3)_2$), 1.20 (d, J = 6.0 Hz, 3H, $OCH(CH_3)_2$). LRMS (CI) *m/z* calcd for $C_{19}H_{19}ClN_3O_2S$ $[M+H]^+$: 388.1; found: 388.1.

(±) Ethyl 7-bromo-4-(furan-2-yl)-2-methyl-1,4-dihydrobenzo[4,5]imidazo[1,2-*a*]pyrimidine-3-carboxylate (15q). Purified by column chromatography (*n*-Hexane–Ethyl Acetate 2:1 – 1:2) to give 45 mg, 18%. Mp: 262 – 264 ºC. ^{1}H NMR (300 MHz, $CDCl_3$), δ (ppm): 7.45 (s, 1H, Ar-H), 7.30 – 7.25 (m, 3H, Ar-H), 6.54 (s, 1H, CH), 6.29 – 6.25 (m, 2H, Ar-H), 4.29 – 4.06 (m, 2H, OCH_2CH_3), 2.69 (s, 3H, CH_3), 1.29 – 1.12 (m, 3H, OCH_2CH_3). LRMS (CI) *m/z* calcd for $C_{18}H_{17}BrN_3O_3$ $[M+H]^+$: 402.0; found: 402.0.

(±) Isopropyl 7-bromo-4-(furan-2-yl)-2-methyl-1,4-dihydrobenzo[4,5]imidazo[1,2-*a*]pyrimidine-3-carboxylate (15r). Purified by column chromatography (*n*-Hexane–Ethyl Acetate 2:1 – 1:2) to give 58 mg, 22%. Mp: 237 – 238 ºC. ^{1}H NMR (300 MHz, $CDCl_3$), δ (ppm): 11.25 (brs, 1H, NH), 7.43 (s, 1H, Ar-H), 7.34 – 7.12 (m, 3H, Ar-H), 6.48 (s, 1H, CH), 6.11 – 6.08 (m, 2H, Ar-H), 5.10 – 4.98 (m, 1H, $OCH(CH_3)_2$), 2.70 (s, 3H, CH_3), 1.26 (d, J = 6.0 Hz, 3H, $OCH(CH_3)_2$), 1.18 (d, J = 6.1 Hz, 3H, $OCH(CH_3)_2$). ^{13}C NMR (75 MHz, $CDCl_3$), δ (ppm): 165.0, 153.0, 147.2, 146.8, 142.2, 130.7, 124.0, 119.1, 115.6, 111.0, 110.4, 107.9, 96.2, 67.6, 50.1, 22.1, 21.8, 19.4. LRMS (EI) (70eV) *m/z* calcd for $C_{19}H_{18}BrN_3O_3$ $[M]^+$: 415.0; found: 414.9.

(±) Ethyl 7-bromo-4-(furan-3-yl)-2-methyl-1,4-dihydrobenzo[4,5]imidazo[1,2-*a*]pyrimidine-3-carboxylate (15s). Purified by column chromatography (*n*-Hexane–Ethyl Acetate 2:1 – 1:2) to give 54 mg, 21%. Mp: 261 – 263 ºC. ^{1}H NMR (750 MHz, DMSO-d_6), δ (ppm): 10.8 (s, 1H, NH), 7.76 (dd, J = 1.6, 0.9 Hz, 1H), 7.69 (d, J = 1.9 Hz, 1H, H-6), 7.47 (t, J = 1.8 Hz, 1H), 7.30 (d, J = 8.4 Hz, 1H, H-9), 7.20 (dd, J = 8.4, 1.9 Hz, 1H, H-8), 6.52 (s, 1H, CH), 6.19 (dd, J = 1.9, 0.9 Hz, 1H), 4.08 (m, 2H, OCH_2CH_3), 2.42 (s, 3H, CH_3), 1.21 (t, J = 7.1 Hz, 3H, OCH_2CH_3). ^{13}C NMR (126 MHz, $CDCl_3$), δ (ppm): 165.7, 147.2, 145.9, 143.8, 139.8, 132.7, 126.1, 125.4, 118.0, 114.0, 112.7, 108.8, 99.0, 60.5, 48.6, 19.7, 14.5. LRMS (CI) *m/z* calcd for $C_{18}H_{17}BrN_3O_3$ $[M+H]^+$: 402.0; found: 402.0.

(±) Isopropyl 7-bromo-4-(furan-3-yl)-2-methyl-1,4-dihydrobenzo[4,5]imidazo[1,2-*a*]pyrimidine-3-carboxylate (15t). Purified by column chromatography (*n*-Hexane–Ethyl Acetate 2:1 – 1:2) to give 57 mg, 22%. Mp: 235 – 236 ºC. ^{1}H NMR (300 MHz, $CDCl_3$), δ (ppm): 11.36 (brs, 1H, NH), 7.47 – 7.30 (m, 5H, Ar-H), 6.47 (s, 1H, CH), 6.20 (s, 1H, Ar-H), 5.29 – 5.05 (m, 1H, $OCH(CH_3)_2$), 2.64 (s, 3H, CH_3), 1.32 (d, J = 6.0 Hz, 3H, $OCH(CH_3)_2$), 1.26 (d, J = 6.0 Hz, 3H, $OCH(CH_3)_2$). ^{13}C NMR (75 MHz, $CDCl_3$), δ (ppm): 166.5, 147.4, 145.3, 144.1, 141.7, 141.0, 134.5, 128.2, 126.8, 118.9, 118.0, 116.1, 109.7, 101.8, 68.2, 53.5, 22.0, 19.2. LRMS (CI) *m/z* calcd for $C_{19}H_{19}BrN_3O_3$ $[M+H]^+$: 416.0; found: 416.0.

(±) Ethyl 7-bromo-2-methyl-4-(thiophen-2-yl)-1,4-dihydrobenzo[4,5]imidazo[1,2-*a*]pyrimidine-3-carboxylate (15u). Purified by column chromatography (*n*-Hexane–Ethyl Acetate 2:1 – 1:2) to give 61 mg, 23%. Mp: 256 – 258 ºC. ^{1}H NMR (300 MHz, $CDCl_3$), δ (ppm): 11.68 (brs, 1H, NH), 7.58 (s, 1H, Ar-H), 7.28 – 7.15 (m, 3H, Ar-H), 6.98 – 6.75 (m, 3H, Ar-H + CH), 4.31 – 4.15 (m, 2H, OCH_2CH_3), 2.63 (s, 3H, CH_3), 1.30 (t, J = 7.1 Hz, 3H, OCH_2CH_3). LRMS (CI) *m/z* calcd for $C_{18}H_{17}BrN_3O_2S$ $[M+H]^+$: 418.0; found: 418.0.

(±) Isopropyl 7-bromo-2-methyl-4-(thiophen-2-yl)-1,4-dihydrobenzo[4,5]imidazo[1,2-*a*]pyrimidine-3-carboxylate (15v). Purified by column chromatography (*n*-Hexane–Ethyl Acetate 2:1 – 1:2) to give 48 mg, 18%. Mp: 250 – 252 ºC. ^{1}H NMR (300 MHz, $CDCl_3$), δ (ppm): 11.68 (brs, 1H, NH), 7.45 (s, 1H, Ar-H), 7.38 – 7.09 (m, 3H, Ar-H), 6.91 (s, 1H, Ar-H), 6.79 – 6.86 (m, 1H, Ar-H), 6.72 (s, 1H, CH), 5.98 – 5.14 (m, 1H, $OCH(CH_3)_2$), 2.84 (s, 3H, CH_3), 1.29 (d, J = 6.0 Hz, 3H, $OCH(CH_3)_2$), 1.19 (d, J = 6.0 Hz, 3H, $OCH(CH_3)_2$). LRMS (CI) *m/z* calcd for $C_{19}H_{19}BrN_3O_2S$ $[M+H]^+$: 432.0; found: 432.0.

(±) Ethyl 7-bromo-2-methyl-4-(thiophen-3-yl)-1,4-dihydrobenzo[4,5]imidazo[1,2-*a*]pyrimidine-3-carboxylate (15w). Purified by column chromatography (*n*-Hexane–Ethyl Acetate 2:1 – 1:2) to give 47 mg, 18%. Mp: 268 – 270 ºC. ^{1}H NMR (300 MHz, $CDCl_3$), δ (ppm): 11.53 (brs, 1H, NH), 7.32 – 7.20 (m, 5H, Ar-H), 6.94 (s, 1H, Ar-H), 6.56 (s, 1H, CH), 4.23 – 4.02 (m, 2H, OCH_2CH_3), 2.65 (s, 3H, CH_3), 1.28 (t, J = 7.1 Hz, 3H, OCH_2CH_3). LRMS (CI) *m/z* calcd for $C_{18}H_{17}BrN_3O_2S$ $[M+H]^+$: 418.0; found: 418.0.

(±) Isopropyl 7-bromo-2-methyl-4-(thiophen-3-yl)-1,4-dihydrobenzo[4,5]imidazo[1,2-*a*]pyrimidine-3-carboxylate (15x). Purified by column chromatography (*n*-Hexane–Ethyl Acetate 2:1 – 1:2) to give 61 mg, 22%. Mp: 264 – 265 ºC. ^{1}H NMR (750 MHz, MeOD), δ (ppm): 7.44 (d, J = 1.9 Hz, 1H, H-6), 7.42 (dd, J = 2.9, 1.4 Hz, 1H), 7.28 (dd, J = 5.1, 2.9 Hz, 1H), 7.27 (d, J = 8.4 Hz, 1H, H-9), 7.23 (dd, J = 8.4, 1.9 Hz, 1H, H-8), 6.92 (dd, J = 5.1, 1.3 Hz, 1H, Ar-H), 6.57 (s, 1H, CH), 4.99 (hept, J = 6.2 Hz, 1H, OC*H*(CH$_3$)$_2$), 2.50 (d, J = 0.7 Hz, 3H, CH$_3$), 1.30 (d, J = 6.2 Hz, 3H, OCH(C*H$_3$*)$_2$), 1.15 (d, J = 6.2 Hz, 3H, OCH(C*H$_3$*)$_2$). LRMS (CI) *m/z* calcd for $C_{19}H_{19}BrN_3O_2S$ [M+H]$^+$: 432.0; found: 432.0.

(±) Ethyl 8-fluoro-4-(furan-2-yl)-2-methyl-1,4-dihydrobenzo[4,5]imidazo[1,2-*a*]pyrimidine-3-carboxylate (16a). Purified by column chromatography (*n*-Hexane–Ethyl Acetate 3:1 – 1:2) to give 57 mg, 19%. Mp: 236 – 238 ºC. ^{1}H NMR (300 MHz, CDCl$_3$), δ (ppm): 9.29 (brs, 1H, NH), 7.29 – 7.09 (m, 4H, Ar-H), 6.47 (s, 1H, Ar-H), 6.25 (d, J = 7.0 Hz, 1H, Ar-H), 6.23 – 6.18 (m, 1H, CH), 4.09 – 4.29 (m, 2H, OC*H$_2$*CH$_3$), 2.69 (s, 3H, CH$_3$), 1.28 (t, J = 7.1 Hz, 3H, OCH$_2$C*H$_3$*). ^{13}C NMR (75 MHz, CDCl$_3$), δ (ppm): 166.9, 158.6, 153.2, 147.9, 147.7, 142.7, 142.3, 132.1, 113.9, 113.2, 110.2, 109.5, 102.7, 97.6, 60.3, 54.7, 18.9, 14.3. HRMS (APCI) *m/z*: calcd for $C_{18}H_{17}FN_3O_3$ [M+H]$^+$, 342.1176; found: 342.1201.

(±) Isopropyl 8-fluoro-4-(furan-2-yl)-2-methyl-1,4-dihydrobenzo[4,5]imidazo[1,2-*a*]pyrimidine-3-carboxylate (16b). Purified by column chromatography (*n*-Hexane–Ethyl Acetate 3:1 – 1:2) to give 61 mg, 19%. Mp: 242 – 243 ºC. ^{1}H NMR (300 MHz, DMSO-*d$_6$*), δ (ppm): 10.77 (brs, 1H, NH), 7.44 (s, 1H, Ar-H), 7.34 – 7.28 (m, 2H, Ar-H), 6.93 – 6.86 (m, 1H, Ar-H), 6.53 – 6.49 (m, 1H, Ar-H), 6.35 – 6.32 (m, 1H, Ar-H), 6.20 (s, 1H, CH), 4.88 – 4.84 (m, 1H, OC*H*(CH$_3$)$_2$), 2.42 (s, 3H, CH$_3$), 1.20 (d, J = 6.0 Hz, 3H, OCH(C*H$_3$*)$_2$), 1.04 (d, J = 6.0 Hz, 3H, OCH(C*H$_3$*)$_2$). ^{13}C NMR (75 MHz, DMSO-*d*$_6$), δ (ppm): 166.6, 160.7, 159.0, 153.1, 148.7, 142.8, 142.6, 132.2, 114.2, 113.1, 110.2, 109.5, 102.7, 97.1, 68.3, 54.8, 21.9, 19.2. HRMS (APCI) *m/z*: calcd for $C_{19}H_{19}FN_3O_3$ [M+H]$^+$, 356.1332; found: 356.1343.

Isopropyl (*R*)-8-fluoro-4-(furan-2-yl)-2-methyl-1,4-dihydrobenzo[4,5]imidazo[1,2-a]pyrimidine-3-carboxylate [(*R*)-16b]. Chiral separation with a 250 mm x 10 mm Lux® 5 µm Amylose-2 (Phenomenex) (isocratic mobile phase *n*-Hexane-*i-Propanol* 9:1) to give an enantiomeric excess of >99%. ^{1}H NMR (300 MHz, CDCl$_3$), δ (ppm): 7.39 (dd, J = 8.8, 4.5 Hz, 1H, Ar-H), 7.35 – 7.26 (m, 2H, Ar-H), 7.19 (dd, J = 8.7, 2.2 Hz, 1H, Ar-H), 7.04 – 6.92 (m, 1H, Ar-H), 6.50 (s, 1H, CH), 6.35 – 6.20 (m, 2H, Ar-H), 5.06 (p, J = 6.3 Hz, 1H, OC*H*(CH$_3$)$_2$), 2.69 (s, 3H, CH$_3$), 1.29 (d, J = 6.3 Hz, 3H, OCH(C*H$_3$*)$_2$), 1.13 (d, J = 6.3 Hz, 3H, OCH(C*H$_3$*)$_2$).

Isopropyl (*S*)-8-fluoro-4-(furan-2-yl)-2-methyl-1,4-dihydrobenzo[4,5]imidazo[1,2-*a*]pyrimidine-3-carboxylate [(*S*)-16b]. Chiral separation with a 250 mm x 10 mm Lux® 5 µm Amylose-2 (Phenomenex) (isocratic mobile phase *n*-Hexane-*i-Propanol* 9:1) to give an enantiomeric excess of 97%. ^{1}H NMR (300 MHz, $CDCl_3$), *δ* (ppm): 7.44 – 7.35 (m, 1H, Ar-H), 7.30 – 7.23 (m, 1H, Ar-H), 7.19 (d, *J* = 8.4 Hz, 1H, Ar-H), 7.04 – 6.92 (m, 1H, Ar-H), 6.50 (s, 1H, CH), 6.29 (m, 2H, Ar-H), 5.10 – 5.01 (m, 1H, $OC\mathit{H}(CH_3)_2$), 2.70 (s, 3H, CH_3), 1.19 – 1.09 (m, 3H, CH_3, $OCH(C\mathit{H_3})_2$), 1.11 (d, *J* = 1.7 Hz, 3H, $OCH(C\mathit{H_3})_2$).

(±) Ethyl 8-fluoro-4-(furan-3-yl)-2-methyl-1,4-dihydrobenzo[4,5]imidazo[1,2-*a*]pyrimidine-3-carboxylate (16c). Purified by column chromatography (*n*-Hexane–Ethyl Acetate 3:1 – 1:2) to give 56 mg, 19%. Mp: 262 – 263 ºC. ^{1}H NMR (300 MHz, $CDCl_3$), *δ* (ppm): 7.39 – 7.14 (m, 3H, Ar-H), 6.94 – 6.82 (m, 1H, Ar-H), 6.49 (s, 1H, CH), 6.29 – 6.19 (m, 2H, Ar-H), 4.22 – 4.12 (m, 2H, $OC\mathit{H_2}CH_3$), 2.71 (s, 3H, CH_3), 1.35 (t, *J* = 7.2 Hz, 3H, $OCH_2C\mathit{H_3}$). HRMS (APCI) *m/z*: calcd for $C_{18}H_{17}FN_3O_3$ $[M+H]^+$, 342.1248; found: 342.1250.

(±) Isopropyl 8-fluoro-4-(furan-3-yl)-2-methyl-1,4-dihydrobenzo[4,5]imidazo[1,2-*a*]pyrimidine-3-carboxylate (16d). Purified by column chromatography (*n*-Hexane–Ethyl Acetate 3:1 – 1:2) to give 46 mg, 15%. Mp: 240 – 242 ºC. ^{1}H NMR (300 MHz, $CDCl_3$), *δ* (ppm): 7.28 – 7.02 (m, 3H, Ar-H), 6.84 – 6.78 (m, 1H, Ar-H), 6.53 (s, 1H, CH), 6.28 – 6.20 (m, 2H, Ar-H), 5.11 – 4.98 (m, 1H, $OC\mathit{H}(CH_3)_2$), 2.69 (s, 3H, CH_3), 1.30 (d, *J* = 6.0 Hz, 3H, $OCH(C\mathit{H_3})_2$), 1.18 (d, *J* = 6.0 Hz, 3H, $OCH(C\mathit{H_3})_2$). ^{13}C NMR (75 MHz, $CDCl_3$), *δ* (ppm): 165.2, 161.3, 158.1, 153.2, 146.8, 142.2, 141.7, 128.1, 110.4, 108.7, 107.8, 102.8, 96.1, 92.3, 76.0, 67.5, 50.1, 22.1, 19.3. HRMS (APCI) *m/z*: calcd for $C_{19}H_{19}FN_3O_3$ $[M+H]^+$, 356.1332; found: 356.1361.

(±) Ethyl 8-fluoro-2-methyl-4-(thiophen-2-yl)-1,4-dihydrobenzo[4,5]imidazo[1,2-*a*]pyrimidine-3-carboxylate (16e). Purified by column chromatography (*n*-Hexane–Ethyl Acetate 3:1 – 1:2) to give 51 mg, 16%. Mp: 253 – 255 ºC. ^{1}H NMR (300 MHz, $CDCl_3$), *δ* (ppm): 11.95 (brs, 1H, NH), 7.25 – 7.10 (m, 2H, Ar-H), 6.95 (s, 2H, Ar-H), 6.93 – 6.82 (m, 2H, Ar-H), 6.89 (s, 1H, CH), 4.25 – 4.09 (m, 2H, $OC\mathit{H_2}CH_3$), 2.70 (s, 3H, CH_3), 1.29 (t, *J* = 7.1 Hz, 3H, $OCH_2C\mathit{H_3}$). LRMS (CI) *m/*z calcd for $C_{18}H_{17}FN_3O_2S$ $[M+H]^+$: 358.1; found: 358.1.

(±) Isopropyl 8-fluoro-2-methyl-4-(thiophen-2-yl)-1,4-dihydrobenzo[4,5]imidazo[1,2-*a*]pyrimidine-3-carboxylate (16f). Purified by column chromatography (*n*-Hexane–Ethyl Acetate 3:1 – 1:2) to give 44 mg, 13%. Mp: 249 – 251 ºC. ^{1}H NMR (300 MHz, $CDCl_3$), δ (ppm): 12.02 (brs, 1H, NH), 7.45 – 7.15 (m, 3H, Ar-H), 6.95 (s, 1H, Ar-H), 6.83 – 6.78 (m, 2H, Ar-H), 6.71 (s, 1H, CH), 5.11 – 4.97 (m, 1H, $OCH(CH_3)_2$), 2.72 (s, 3H, CH_3), 1.31 (d, J = 6.1 Hz, 3H, $OCH(CH_3)_2$), 1.23 (d, J = 6.0 Hz, 3H, $OCH(CH_3)_2$). LRMS (CI) *m/z* calcd for $C_{19}H_{19}FN_3O_2S$ [M+H]$^+$: 372.1; found: 372.1.

(±) Ethyl 8-fluoro-2-methyl-4-(thiophen-3-yl)-1,4-dihydrobenzo[4,5]imidazo[1,2-*a*]pyrimidine-3-carboxylate (16g). Purified by column chromatography (*n*-Hexane–Ethyl Acetate 3:1 – 1:2) to give 55 mg, 17%. Mp: 260 – 261 ºC. ^{1}H NMR (300 MHz, $CDCl_3$), δ (ppm): 7.22 – 6.82 (m, 6H, Ar-H), 6.45 (s, 1H, CH), 4.22 – 4.02 (m, 2H, OCH_2CH_3), 2.65 (s, 3H, CH_3), 1.30 (t, J = 7.0 Hz, 3H, OCH_2CH_3). LRMS (CI) *m/*z calcd for $C_{18}H_{17}FN_3O_2S$ [M+H]$^+$: 358.1; found: 358.1.

(±) Isopropyl 8-fluoro-2-methyl-4-(thiophen-3-yl)-1,4-dihydrobenzo[4,5]imidazo[1,2-*a*]pyrimidine-3-carboxylate (16h). Purified by column chromatography (*n*-Hexane–Ethyl Acetate 3:1 – 1:1) to give 46 mg, 14%. Mp: 253 – 255 ºC. ^{1}H NMR (300 MHz, $CDCl_3$), δ (ppm): 11.95 (brs, 1H, NH), 7.42 – 7.16 (m, 4H, Ar-H), 6.98 – 6.89 (m, 2H, Ar-H), 6.62 – 6.54 (m, 1H, CH), 5.18 – 4.96 (m, 1H, $OCH(CH_3)_2$), 2.69 (s, 3H, CH_3), 1.32 (d, J = 6.4 Hz, 3H, $OCH(CH_3)_2$), 1.22 (d, J = 6.2 Hz, 3H, $OCH(CH_3)_2$). ^{13}C NMR (75 MHz, $CDCl_3$), δ (ppm): 166.5, 160.9, 158.9, 149.2, 147.3, 142.6, 140.8, 132.0, 126.9, 126.6, 123.2, 114.1, 113.5, 102,5, 101.6, 68.6, 56.8, 22.1, 19.3. LRMS (CI) *m/z* calcd for $C_{19}H_{19}FN_3O_2S$ [M+H]$^+$: 372.1; found: 372.1.

(±) Ethyl 8-chloro-4-(furan-2-yl)-2-methyl-1,4-dihydrobenzo[4,5]imidazo[1,2-*a*]pyrimidine-3-carboxylate (16i). Purified by column chromatography (*n*-Hexane–Ethyl Acetate 3:1 – 1:1) to give 60 mg, 21%. Mp: 232 – 234 ºC. ^{1}H NMR (300 MHz, DMSO-d_6), δ (ppm): 10.95 (brs, 1H, NH), 7.49 – 7.38 (m, 3H, Ar-H), 7.10 – 7.01 (m, 1H, Ar-H), 6.62 – 6.49 (m, 1H, Ar-H), 6.44 – 6.33 (m, 1H, Ar-H), 6.25 (s, 1H, CH), 4.18 – 3.96 (m, 2H, OCH_2CH_3), 2.46 (s, 3H, CH_3), 1.26 (t, J = 7.0 Hz, 3H, OCH_2CH_3). ^{13}C NMR (75 MHz, DMSO-d_6), δ (ppm): 165.4, 152.7, 147.9, 147.3, 143.8, 143.2, 130.9, 126.8, 120.5, 116.8, 111.4, 110.8, 108.4, 95.1, 59.9, 49.6, 19.1, 14.6. HRMS (APCI) *m/z* calcd for $C_{18}H_{17}ClN_3O_3$ [M+H]$^+$: 358.0880; found: 358.0911.

(±) Isopropyl 8-chloro-4-(furan-2-yl)-2-methyl-1,4-dihydrobenzo[4,5]imidazo[1,2-*a*]pyrimidine-3-carboxylate (16j). Purified by column chromatography (*n*-Hexane–Ethyl Acetate 3:1 – 1:1) to give 62 mg, 21%. Mp: 252 – 253 °C. ^{1}H NMR (300 MHz, DMSO-d_6), δ (ppm): 10.90 (brs, 1H, NH), 7.45 – 7.32 (m, 2H, Ar-H), 7.06 – 7.02 (m, 1H, Ar-H), 6.55 (s, 1H, Ar-H), 6.45 (s, 1H, Ar-H), 6.33 (s, 1H, Ar-H), 5.74 (s, 1H, CH), 4.92 – 4.87 (m, 1H, OC*H*(CH_3)$_2$), 2.44 (s, 3H, CH_3), 1.21 (d, J = 6.3 Hz, 3H, OCH(CH_3)$_2$), 1.05 (d, J = 6.0 Hz, 3H, OCH(CH_3)$_2$). ^{13}C NMR (75 MHz, $CDCl_3$) δ (ppm): 165.0, 152.9, 147.0, 146.9, 142.3, 139.7, 132.3, 126.6, 123.1, 116.9, 110.4, 110.1, 107.9, 96.1, 67.5, 50.1, 22.1, 21.7, 19.2. HRMS (APCI) *m/z* calcd for $C_{19}H_{19}ClN_3O_3$ [M+H]$^+$: 372.1037; found: 372.1042.

Isopropyl (*R)*-8-chloro-4-(furan-2-yl)-2-methyl-1,4-dihydrobenzo[4,5]imidazo[1,2-*a*]pyrimidine-3-carboxylate [(*R*)-16j]. Chiral separation with a 250 mm x 20 mm Chiralpak® 5µm IE-3 (DAICEL) (isocratic mobile phase *n*-Hexane-*i-Propanol* 9:1) to give an enantiomeric excess of 97%. ^{1}H NMR (300 MHz, DMSO-d_6), δ (ppm): 7.47 – 7.33 (m, 2H, Ar-H), 7.31 – 7.23 (m, 1H, Ar-H), 7.21 – 7.14 (m, 1H, Ar-H), 6.49 (s, 1H, CH), 6.33 – 6.23 (m, 2H, Ar-H), 5.06 (qd, J = 6.5, 5.7 Hz, 1H, OC*H*(CH_3)$_2$), 2.70 (s, 3H, CH_3), 1.32 – 1.26 (m, 3H OCH(CH_3)$_2$), 1.13 (d, J = 6.3, 3H, OCH(CH_3)$_2$).

Isopropyl (*S*)-*8*-chloro-4-(furan-2-yl)-2-methyl-1,4-dihydrobenzo[4,5]imidazo[1,2-*a*]pyrimidine-3-carboxylate [(*S*)-16j]. Chiral separation with a 250 mm x 20 mm Chiralpak® 5µm IE-3 (DAICEL) (isocratic mobile phase *n*-Hexane-*i-Propanol* 9:1) to give an enantiomeric excess of 98%. ^{1}H NMR (300 MHz, $CDCl_3$), δ (ppm): 7.57 – 7.44 (m, 2H, Ar-H), 7.42 – 7.32 (m, 2H, Ar-H), 6.55 (s, 1H, Ar-H), 6.39 (s, 1H, Ar-H), 6.33 (s, 1H, Ar-H), 5.12 – 5.02 (m, 1H, OC*H*(CH_3)$_2$), 2.66 (s, 3H, CH_3), 1.33 – 1.20 (m, 3H, OCH(CH_3)$_2$), 1.15 (d, J = 6.7 Hz, 3H, OCH(CH_3)$_2$).

(±) Ethyl 8-chloro-4-(furan-3-yl)-2-methyl-1,4-dihydrobenzo[4,5]imidazo[1,2-*a*]pyrimidine-3-carboxylate (16k). Purified by column chromatography (*n*-Hexane–Ethyl Acetate 3:1 – 1:1) to give 53 mg, 19%. Mp: 241 – 243 °C. ^{1}H NMR (300 MHz, $CDCl_3$), δ (ppm): 7.38 (s, 1H, Ar-H), 7.28 – 7.18 (m, 2H, Ar-H), 7.10 – 7.04 (m, 1H, Ar-H), 6.48 (s, 1H, CH), 6.11 – 6.08 (m, 2H, Ar-H), 4.44 – 4.02 (m, 2H, OCH_2CH$_3$), 2.70 (s, 3H, CH_3), 1.18 (t, J = 7.1 Hz, 3H, OCH$_2$CH_3). ^{13}C NMR (75 MHz, $CDCl_3$), δ (ppm): 168.0, 149.0, 146.6, 143.2, 141.0, 140.6, 132.5, 130.9, 128.3, 122.9, 118.1, 113.5, 109.7, 101.3, 60.1, 53.5, 18.9, 14.4. HRMS (APCI) *m/z* calcd for $C_{18}H_{17}ClN_3O_3$ [M+H]$^+$: 358.0940; found: 358.0950.

(±) Isopropyl 8-chloro-4-(furan-3-yl)-2-methyl-1,4-dihydrobenzo[4,5]imidazo[1,2-*a*]pyrimidine-3-carboxylate (16l). Purified by column chromatography (*n*-Hexane–Ethyl Acetate 3:1 – 1:1) to give 50 mg, 17%. Mp: 256 – 258 ºC. ^{1}H NMR (300 MHz, $CDCl_3$), δ (ppm): 11.95 (brs, 1H, NH), 7.48 (s, 1H, Ar-H), 7.38 (s, 1H, Ar-H), 7.25 (s, 1H, Ar-H), 7.21 – 7.06 (m, 2H, Ar-H), 6.52 (s, 1H, CH), 6.11 (s, 1H, Ar-H), 5.11 – 5.05 (m, 1H, $OC\mathit{H}(CH_3)_2$), 2.70 (s, 3H, CH_3), 1.35 (d, J = 6.1 Hz, 3H, $OCH(C\mathit{H_3})_2$), 1.31 (d, J = 6.0 Hz, 3H, $OCH(C\mathit{H_3})_2$). ^{13}C NMR (75 MHz, $CDCl_3$), δ (ppm): 165.3, 147.8, 145.6, 142.3, 141.7, 130.3, 128.2, 126.4, 126.1, 122.7, 121.3, 116.3, 110.2, 99.4, 67.8, 52.1, 21.9, 19.5. HRMS (APCI) *m/z* calcd. for $C_{19}H_{19}ClN_3O_3$ $[M+H]^+$: 372.1109; found: 372.1111.

(*S*) Isopropyl 8-chloro-4-(furan-3-yl)-2-methyl-1,4-dihydrobenzo[4,5]imidazo[1,2-*a*]pyrimidine-3-carboxylate [(*S*)-16l]. Chiral separation with a 250 mm x 20 mm Chiralpak® 5µm IE-3 (DAICEL) (isocratic mobile phase *n*-Hexane-*i-Propanol* 9:1) to give an enantiomeric excess of 98%. ^{1}H NMR (300 MHz, $CDCl_3$), δ (ppm): 11.90 (brs, 1H, NH), 7.49 (s, 1H, Ar-H), 7.39 (s, 1H, Ar-H), 7.31 (s, 1H, Ar-H), 7.24 – 7.02 (m, 2H, Ar-H), 6.59 (s, 1H, CH), 6.15 (s, 1H, Ar-H), 5.11 – 5.05 (m, 1H, $OC\mathit{H}(CH_3)_2$), 2.70 (s, 3H, CH_3), 1.35 (d, J = 6.1 Hz, 3H, $OCH(C\mathit{H_3})_2$), 1.31 (d, J = 6.0 Hz, 3H, $OCH(C\mathit{H_3})_2$).

(*R*) Isopropyl 8-chloro-4-(furan-3-yl)-2-methyl-1,4-dihydrobenzo[4,5]imidazo[1,2-a]pyrimidine-3-carboxylate [(*R*)-16l]. Chiral separation with a 250 mm x 20 mm Chiralpak® 5µm IE-3 (DAICEL) (isocratic mobile phase *n*-Hexane-*i-Propanol* 9:1) to give an enantiomeric excess of 97%. ^{1}H NMR (300 MHz, $CDCl_3$), δ (ppm): 11.91 (brs, 1H, NH), 7.48 (s, 1H, Ar-H), 7.33 (s, 1H, Ar-H), 7.22 (s, 1H, Ar-H), 7.21 – 7.06 (m, 2H, Ar-H), 6.52 (s, 1H, CH), 6.09 (s, 1H, Ar-H), 5.11 – 5.04 (m, 1H, $OC\mathit{H}(CH_3)_2$), 2.69 (s, 3H, CH_3), 1.35 (d, J = 6.1 Hz, 3H, $OCH(C\mathit{H_3})_2$), 1.31 (d, J = 6.0 Hz, 3H, $OCH(C\mathit{H_3})_2$).

(±) Ethyl 8-chloro-2-methyl-4-(thiophen-2-yl)-1,4-dihydrobenzo[4,5]imidazo[1,2-*a*]pyrimidine-3-carboxylate (16m). Purified by column chromatography (*n*-Hexane–Ethyl Acetate 3:1 – 1:1) to give 64 mg, 22%. Mp: 247 – 249 ºC. ^{1}H NMR (300 MHz, $CDCl_3$), δ (ppm): 7.52 – 7.46 (m, 1H, Ar-H), 7.28 – 7.05 (m, 3H, Ar-H), 6.96 – 6.9 (m, 1H, Ar-H), 6.83 – 6.81 (m, 1H, Ar-H), 6.69 (s, 1H, CH), 4.25 – 4.10 (m, 2H, $OC\mathit{H_2}CH_3$), 2.72 (s, 3H, CH_3), 1.33 (t, J = 7.0 Hz, 3H, $OCH_2C\mathit{H_3}$). LRMS (EI) (70eV) *m/z* calcd for $C_{18}H_{16}ClN_3O_2S$ $[M]^+$: 373.1; found: 372.9.

(±) Isopropyl 8-chloro-2-methyl-4-(thiophen-2-yl)-1,4-dihydrobenzo[4,5]imidazo[1,2-*a*]pyrimidine-3-carboxylate (16n). Purified by column chromatography (*n*-Hexane–Ethyl Acetate 3:1 – 1:1) to give 52 mg, 17%. Mp: 248 – 249 ºC. ^{1}H NMR (300 MHz, $CDCl_3$), δ (ppm): 7.42 (s, 2H, Ar-H), 7.26 – 7.08 (m, 3H, Ar-H), 6.90 – 6.84 (m, 1H, Ar-H), 6.78 (s, 1H, CH), 5.18 – 5.05 (m, 1H, $OCH(CH_3)_2$), 2.72 (s, 3H, CH_3), 1.34 (d, J = 6.2 Hz, 3H, $OCH(CH_3)_2$), 1.18 (d, J = 6.2 Hz, 3H, $OCH(CH_3)_2$). LRMS (EI) (70eV) *m/z* calcd for $C_{19}H_{18}ClN_3O_2S$ $[M]^+$: 387.1; found: 386.9.

(±) Ethyl 8-chloro-2-methyl-4-(thiophen-3-yl)-1,4-dihydrobenzo[4,5]imidazo[1,2-*a*]pyrimidine-3-carboxylate (16o). Purified by column chromatography (*n*-Hexane–Ethyl Acetate 3:1 – 1:1) to give 44 mg, 15%. Mp: 255 – 256 ºC. ^{1}H NMR (300 MHz, $CDCl_3$), δ (ppm): 11,65 (brs, 1H, NH), 7,42 (s, 1H, Ar-H), 7.19 – 6.94 (m, 5H, Ar-H), 6.60 (s, 1H, CH), 4.25 – 4.02 (m, 2H, OCH_2CH_3), 2.68 (s, 3H, CH_3), 1.31 (t, J = 7.1 Hz, 3H, OCH_2CH_3). LRMS (CI) *m/z* calcd for $C_{18}H_{17}ClN_3O_2S$ $[M+H]^+$: 374.1; found: 374.1.

(±) Isopropyl 8-chloro-2-methyl-4-(thiophen-3-yl)-1,4-dihydrobenzo[4,5]imidazo[1,2-*a*]pyrimidine-3-carboxylate (16p). Purified by column chromatography (*n*-Hexane–Ethyl Acetate 3:1 – 1:1) to give 50 mg, 16%. Mp: 248 – 250 ºC. ^{1}H NMR (300 MHz, $CDCl_3$), δ (ppm): 12.02 (brs, 1H, NH), 7.39 (s, 1H, Ar-H), 7.22 – 6.98 (m, 5H, Ar-H), 6.76 (s, 1H, CH), 5.19 – 5.05 (m, 1H, $OCH(CH_3)_2$), 2.69 (s, 3H, CH_3), 1.31 (d, J = 6.1 Hz, 3H, $OCH(CH_3)_2$), 1.22 (d, J = 6.0 Hz, 3H, $OCH(CH_3)_2$). ^{13}C NMR (75 MHz, $CDCl_3$), δ (ppm): 165.3, 147.8, 145.6, 142.3, 141.7, 130.3, 128.2, 126.4 126.1, 122.7, 121.2, 116.3, 110.2, 99.5, 67.8, 52.1, 22.2, 21.9, 19.5. LRMS (CI) *m/z* calcd for $C_{19}H_{19}ClN_3O_2S$ $[M+H]^+$: 388.1; found: 388.1.

(±) Ethyl 8-bromo-4-(furan-2-yl)-2-methyl-1,4-dihydrobenzo[4,5]imidazo[1,2-*a*]pyrimidine-3-carboxylate (16q). Purified by column chromatography (*n*-Hexane–Ethyl Acetate 2:1 – 1:2) to give 45 mg, 18%. Mp: 242 – 243 ºC. ^{1}H NMR (300 MHz, $CDCl_3$), δ (ppm): 11,45 (brs, 1H, NH), 7.61 (s, 1H, Ar-H), 7.30 – 7.25 (m, 3H, Ar-H), 6.53 (s, 1H, CH), 6.28 – 6.24 (m, 2H, Ar-H), 4.26 – 4.12 (m, 2H, OCH_2CH_3), 2.71 (s, 3H, CH_3), 1.26 (t, J = 7.1 Hz, 3H, OCH_2CH_3). ^{13}C NMR (75 MHz, $CDCl_3$), δ (ppm): 167.1, 152.9, 146.7, 142.5, 141.8, 132.7, 124.3, 119.2, 118.5, 114.3, 111.0, 110.4, 97.3, 108.0, 60.3, 50.0, 19.5, 14.3. LRMS (CI) *m/z* calcd for $C_{18}H_{17}BrN_3O_3$ $[M+H]^+$: 402.0; found: 402.0.

(±) Isopropyl 8-bromo-4-(furan-2-yl)-2-methyl-1,4-dihydrobenzo[4,5]imidazo[1,2-*a*]pyrimidine-3-carboxylate (16r). Purified by column chromatography (*n*-Hexane–Ethyl Acetate 2:1 – 1:2) to give 58 mg, 22%. Mp: 245 – 247 ºC. ^{1}H NMR (300 MHz, $CDCl_3$), δ (ppm): 11.35 (brs, 1H, NH), 7.42 (s, 1H, Ar-H), 7.32 – 7.10 (m, 3H, Ar-H), 6.49 (s, 1H, CH), 6.13 – 6.08 (m, 2H, Ar-H), 5.11 – 4.98 (m, 1H, OC*H*$(CH_3)_2$), 2.69 (s, 3H, CH_3), 1.25 (d, J = 6.0 Hz, 3H, OCH(CH_3)$_2$), 1.12 (d, J = 6.1 Hz, 3H, OCH(CH_3)$_2$). ^{13}C NMR (75 MHz, $CDCl_3$), δ (ppm): 165.0, 152.8, 147.2, 146.9, 142.3, 140.0, 132.7, 125.9, 117.3, 113.8, 112.9, 110.4, 107.9, 96.1, 67.5, 50.1, 22.1, 21.7, 19.2. HRMS (APCI) *m/z* calcd for $C_{19}H_{19}BrN_3O_3$ [M+H]$^+$: 416,0618; found: 416.0605.

Isopropyl (*R*)-8-bromo-4-(furan-2-yl)-2-methyl-1,4-dihydrobenzo[4,5]imidazo[1,2-*a*]pyrimidine-3-carboxylate [(*R*)-16r]. Chiral separation with a 250 mm x 20 mm Chiralpak® 5µm IE-3 (DAICEL) (isocratic mobile phase *n*-Hexane-*i-Propanol* 9:1) to give an enantiomeric excess of >99%. ^{1}H NMR (300 MHz, $CDCl_3$), δ (ppm): 11.35 (brs, 1H, NH), 7.41 (s, 1H, Ar-H), 7.33 – 7.11 (m, 3H, Ar-H), 6.50 (s, 1H, CH), 6.13 – 6.08 (m, 2H, Ar-H), 5.11 – 4.98 (m, 1H, OC*H*$(CH_3)_2$), 2.69 (s, 3H, CH_3), 1.25 (d, J = 6.2 Hz, 3H, OCH(CH_3)$_2$), 1.12 (d, J = 6.2 Hz, 3H, OCH(CH_3)$_2$).

Isopropyl (*S*)-*8*-bromo-4-(furan-2-yl)-2-methyl-1,4-dihydrobenzo[4,5]imidazo[1,2-*a*]pyrimidine-3-carboxylate [(*S*)-16r]. Chiral separation with a 250 mm x 20 mm Chiralpak® 5µm IE-3 (DAICEL) (isocratic mobile phase *n*-Hexane-*i-Propanol* 9:1) to give an enantiomeric excess of >99%. ^{1}H NMR (300 MHz, $CDCl_3$), δ (ppm): 11.35 (brs, 1H, NH), 7.42 (s, 1H, Ar-H), 7.32 – 7.10 (m, 3H, Ar-H), 6.50 (s, 1H, CH), 6.13 – 6.08 (m, 2H, Ar-H), 5.11 – 4.98 (m, 1H, OC*H*$(CH_3)_2$), 2.69 (s, 3H, CH_3), 1.25 (d, J = 6.2 Hz, 3H, OCH(CH_3)$_2$), 1.12 (d, J = 6.0 Hz, 3H, OCH(CH_3)$_2$).

(±) Ethyl 8-bromo-4-(furan-3-yl)-2-methyl-1,4-dihydrobenzo[4,5]imidazo[1,2-*a*]pyrimidine-3-carboxylate (16s). Purified by column chromatography (*n*-Hexane–Ethyl Acetate 2:1 – 1:2) to give 54 mg, 21%. Mp: 228 – 230 ºC. ^{1}H NMR (750 MHz, DMSO-d_6), δ (ppm): 10.9 (s, 1H, NH), 7.53 (d, J = 1.9 Hz, 1H, H-9), 7.45 (dd, J = 1.8, 0.9 Hz, 1H), 7.41 (d, J = 8.4 Hz, 1H, H-6), 7.17 (dd, J = 8.4, 1.9 Hz, 1H, H-7), 6.60 (d, J = 0.8 Hz, 1H, CH), 6.46 (dd, J = 3.3, 0.9 Hz, 1H, Ar-H), 6.33 (dd, J = 3.3, 1.8 Hz, 1H, Ar-H), 4.10 (dq, J = 10.9, 7.1 Hz, 1H, OCH_2CH$_3$), 4.03 (dq, J = 10.9, 7.1 Hz, 1H, OCH_2CH$_3$), 2.45 (d, J = 0.7 Hz, 3H, CH_3), 1.16 (t, J = 7.1 Hz, 3H, OCH$_2$CH_3). ^{13}C NMR (126 MHz, $CDCl_3$), δ (ppm): 165.7, 147.4, 145.7, 143.8, 139.8, 131.7, 130.5, 125.4, 124.4, 119.6, 115.9, 110.8, 108.8, 99.2, 60.6, 48.6, 19.8, 14.5. HRMS (APCI) *m/z* calcd for $C_{18}H_{17}BrN_3O_3$ [M+H]$^+$: 402.0375; found: 402.0382.

Ethyl (*S*)-8-bromo-4-(furan-3-yl)-2-methyl-1,4-dihydrobenzo[4,5]imidazo[1,2-*a*]pyrimidine-3-carboxylate [(*S*)-16s]. Chiral separation with a 250 mm x 20 mm Chiralpak® 5µm IE-3 (DAICEL) (isocratic mobile phase *n*-Hexane-*i-Propanol* 4:1) to give an enantiomeric excess of >99%. ^{1}H NMR (300 MHz, $CDCl_3$), δ (ppm): 11.60 (brs, 1H, NH), 7.62 (s, 1H, Ar-H), 7.36 (s, 1H, Ar-H), 7.29 – 7.09 (m, 2H, Ar-H), 7.06 – 6.98 (m, 1H, Ar-H), 6.50 (s, 1H, CH), 6.15 (s, 1H, Ar-H), 4.26 – 4.08 (m, 2H, $OC\mathit{H_2}CH_3$), 2.62 (s, 3H, CH_3), 1.31 (t, J = 7.1 Hz, 3H, $OCH_2C\mathit{H_3}$).

Ethyl (*R*)-8-bromo-4-(furan-3-yl)-2-methyl-1,4-dihydrobenzo[4,5]imidazo[1,2-*a*]pyrimidine-3-carboxylate [(*R*)-16s]. Chiral separation with a 250 mm x 20 mm Chiralpak® 5µm IE-3 (DAICEL) (isocratic mobile phase *n*-Hexane-*i-Propanol* 4:1) to give an enantiomeric excess of 98%. ^{1}H NMR (300 MHz, $CDCl_3$), δ (ppm): 11.61 (brs, 1H, NH), 7.60 (s, 1H, Ar-H), 7.38 (s, 1H, Ar-H), 7.30 – 7.10 (m, 2H, Ar-H), 7.06 – 7.00 (m, 1H, Ar-H), 6.50 (s, 1H, CH), 6.15 (s, 1H, Ar-H), 4.31 – 4.04 (m, 2H, $OC\mathit{H_2}CH_3$), 2.62 (s, 3H, CH_3), 1.31 (t, J = 7.2 Hz, 3H, $OCH_2C\mathit{H_3}$). HRMS (APCI) *m/z* calcd for $C_{18}H_{17}BrN_3O_3$ $[M+H]^+$: 402.0375; found: 402.0402.

(±) Isopropyl 8-bromo-4-(furan-3-yl)-2-methyl-1,4-dihydrobenzo[4,5]imidazo[1,2-*a*]pyrimidine-3-carboxylate (16t). Purified by column chromatography (*n*-Hexane–Ethyl Acetate 2:1) to give 57 mg, 22%. Mp: 248 – 249 ºC. ^{1}H NMR (300 MHz, $CDCl_3$), δ (ppm): 11.36 (brs, 1H, NH), 7.61 (s, 1H, Ar-H), 7.37 (s, 1H, Ar-H), 7.31 – 7.12 (m, 3H, Ar-H), 6.48 (s, 1H, CH), 6.19 (s, 1H, Ar-H), 5.19 – 5.08 (m, 1H, $OC\mathit{H}(CH_3)_2$), 2.66 (s, 3H, CH_3), 1.32 (d, J = 6.0 Hz, 3H, $OCH(C\mathit{H_3})_2$), 1.27 (d, J = 6.0 Hz, 3H, $OCH(C\mathit{H_3})_2$). ^{13}C NMR (75 MHz, $CDCl_3$), δ (ppm): 166.7, 148.7, 146.9, 143.2, 141.1, 140.73, 133.4, 128.3, 126.1, 118.9, 118.3, 114.1, 109.8, 101.8, 68.1, 53.5, 21.9, 20.9, 19.0. HRMS (APCI) *m/z* calcd for $C_{19}H_{17}BrN_3O_3$ $[M-1]^-$: 414.0459; found: 414.0457.

(±) Ethyl 8-bromo-2-methyl-4-(thiophen-2-yl)-1,4-dihydrobenzo[4,5]imidazo[1,2-*a*]pyrimidine-3-carboxylate (16u). Purified by column chromatography (*n*-Hexane–Ethyl Acetate 2:1 – 1:2) to give 61 mg, 23%. Mp: 236 – 238 ºC. ^{1}H NMR (300 MHz, $CDCl_3$), δ (ppm): 11.86 (brs, 1H, NH), 7.58 (s, 1H, Ar-H), 7.25 – 7.03 (m, 3H, Ar-H), 6.99 – 6.65 (m, 3H, Ar-H + CH), 4.28 – 4.16 (m, 2H, $OC\mathit{H_2}CH_3$), 2.65 (s, 3H, CH_3), 1.28 (t, J = 7.0 Hz, 3H, $OCH_2C\mathit{H_3}$). LRMS (CI) *m/z* calcd for $C_{18}H_{17}BrN_3O_2S$ $[M+H]^+$: 418.0; found: 418.0.

(±) Isopropyl 8-bromo-2-methyl-4-(thiophen-2-yl)-1,4-dihydrobenzo[4,5]imidazo[1,2-*a*] pyrimidine-3-carboxylate (16v). Purified by column chromatography (*n*-Hexane–Ethyl Acetate 2:1 – 1:2) to give 48 mg, 18%. Mp: 224 – 226 ºC. ^{1}H NMR (300 MHz, $CDCl_3$), δ (ppm): 11.89 (brs, 1H, NH), 7.60 (s, 1H, Ar-H), 7.30 – 7.05 (m, 3H, Ar-H), 6.89 (s, 1H, Ar-H), 6.85 – 6.75 (m, 1H, Ar-H), 6.70 (s, 1H, CH), 5.96 – 5.12 (m, 1H, $OC\mathit{H}(CH_3)_2$), 2.81 (s, 3H, CH_3), 1.32 (d, J = 6.1 Hz, 3H, $OCH(C\mathit{H_3})_2$), 1.21 (d, J = 6.1 Hz, 3H, $OCH(C\mathit{H_3})_2$). ^{13}C NMR (75 MHz, $CDCl_3$), δ (ppm): 165.1, 158.5, 147.4, 145.9, 144.0, 142.7, 126.5, 125.6, 125.4, 124.0, 119.4, 115.7, 110.9, 99.6, 67.9, 51.7, 22.2, 21.8, 19.5. LRMS (CI) *m/z* calcd for $C_{19}H_{19}BrN_3O_2S$ $[M+H]^+$: 432.0; found: 432.0.

(±) Ethyl 8-bromo-2-methyl-4-(thiophen-3-yl)-1,4-dihydrobenzo[4,5]imidazo[1,2-*a*]pyrimidine-3-carboxylate (16w). Purified by column chromatography (*n*-Hexane–Ethyl Acetate 2:1 – 1:2) to give 47 mg, 18%. Mp: 252 – 254 ºC. ^{1}H NMR (300 MHz, $CDCl_3$), δ (ppm): 11.53 (brs, 1H, NH), 7.31 (s, 2H, Ar-H), 7.18 – 7.15 (m, 3H, Ar-H), 6.96 (s, 1H, Ar-H), 6.58 (s, 1H, CH), 4.18 – 4.02 (m, 2H, $OC\mathit{H_2}CH_3$), 2.68 (s, 3H, CH_3), 1.27 (t, J = 7.0 Hz, 3H, $OCH_2C\mathit{H_3}$). LRMS (CI) *m/z* calcd for $C_{18}H_{17}BrN_3O_2S$ $[M+H]^+$: 418.0; found: 417.9.

(±) Isopropyl 8-bromo-2-methyl-4-(thiophen-3-yl)-1,4-dihydrobenzo[4,5]imidazo[1,2-*a*]pyrimidine-3-carboxylate (16x). Purified by column chromatography (*n*-Hexane–Ethyl Acetate 2:1 – 1:2) to give 61 mg, 22%. Mp: 248 – 249 ºC. ^{1}H NMR (750 MHz, MeOD), δ (ppm): 7.50 (d, J = 1.8 Hz, 1H, H-9), 7.40 (dd, J = 3.0, 1.3 Hz, 1H), 7.27 (dd, J = 5.1, 3.0 Hz, 1H), 7.21 (d, J = 8.5 Hz, 1H, H-6), 7.15 (dd, J = 8.5, 1.8 Hz, 1H, H-7), 6.92 (dd, J = 5.1, 1.3 Hz, 1H, Ar-H), 6.58 (s, 1H, CH), 5.00 (hept, J = 6.3 Hz, 1H, $OC\mathit{H}(CH_3)_2$), 2.50 (d, J = 0.7 Hz, 3H, CH_3), 1.30 (d, J = 6.3 Hz, 3H, $OCH(C\mathit{H_3})_2$), 1.14 (d, J = 6.3 Hz, 3H, $OCH(C\mathit{H_3})_2$). ^{13}C NMR (126 MHz, MeOD), δ (ppm): 166.8, 148.1, 147.0, 144.5, 143.1, 132.1, 127.5, 127.0, 124.8, 124.3, 120.3, 116.3, 112.3, 100.7, 69.0, 53.2, 22.3, 22.0, 18.9.LRMS (CI) *m/z* calcd for $C_{19}H_{19}BrN_3O_2S$ $[M+H]^+$: 432.0; found: 432.0.

(±) Ethyl 7-fluoro-2-methyl-4-(oxazol-4-yl)-1,4-dihydrobenzo[4,5]imidazo[1,2-*a*]pyrimidine-3-carboxylate (17a). Purified by column chromatography (*n*-Hexane–Ethyl Acetate 1:1) to give 33 mg, 12%. Mp: 253 – 254 ºC. ^{1}H NMR (300 MHz, $CDCl_3$), δ (ppm): 7.79 – 7.66 (m, 1H, Ar-H), 7.55 – 7.32 (m, 1H, Ar-H), 7.32 – 7.13 (m, 1H, Ar-H), 7.13 – 7.02 (m, 1H, Ar-H), 7.02 – 6.80 (m, 1H, Ar-H), 6.53 (s, 1H, CH), 4.36 – 4.04 (m, 2H, $OC\mathit{H_2}CH_3$), 2.73 (s, 3H, CH_3), 1.32 (t, J = 7.1 Hz, 3H, $OCH_2C\mathit{H_3}$). LRMS (CI) *m/z* calcd for $C_{17}H_{16}FN_4O_3$ $[M+H]^+$: 343.1; found: 343.2.

(±) Isopropyl 7-fluoro-2-methyl-4-(oxazol-4-yl)-1,4-dihydrobenzo[4,5]imidazo[1,2-*a*]pyrimidine-3-carboxylate (17b). Purified by column chromatography (*n*-Hexane–Ethyl Acetate 1:1) to give 45 mg, 25%. Mp: 240 – 241 ºC. ^{1}H NMR (300 MHz, $CDCl_3$), δ (ppm): 11.97 (s, 1H, NH), 7.77 – 7.68 (m, 1H, Ar-H), 7.41 (dd, J = 8.7, 4.5 Hz, 1H, Ar-H), 7.28 (s, 1H, Ar-H), 7.19 (dd, J = 9.5, 2.4 Hz, 1H, Ar-H), 7.02 – 6.83 (m, 1H, Ar-H), 6.57 – 6.49 (m, 1H, CH), 5.22 – 5.01 (m, 1H, $OC\mathit{H}(CH_3)_2$), 2.73 (s, 3H, CH_3), 1.33 (d, J = 6.3 Hz, 3H, $OCH(C\mathit{H_3})_2$), 1.23 (d, J = 6.2 Hz, 3H, $OCH(C\mathit{H_3})_2$). LRMS (CI) *m/z* calcd for $C_{18}H_{18}FN_4O_3$ $[M+H]^+$: 357.1; found: 357.4.

(±) Ethyl 7-chloro-2-methyl-4-(oxazol-4-yl)-1,4-dihydrobenzo[4,5]imidazo[1,2-*a*]pyrimidine-3-carboxylate (17c). Purified by column chromatography (*n*-Hexane–Ethyl Acetate 1:1) to give 33 mg, 12%. Mp: 256 – 258 ºC. ^{1}H NMR (300 MHz, $CDCl_3$), δ (ppm): 7.79 – 7.63 (m, 1H, Ar-H), 7.59 – 7.21 (m, 3H, Ar-H), 7.21 – 7.00 (m, 1H, Ar-H), 6.55 (s, 1H, CH), 4.23 (q, J = 7.2 Hz, 2H, $OC\mathit{H_2}CH_3$), 2.73 (s, 3H, CH_3), 1.32 (t, J = 7.2 Hz, 3H, $OCH_2C\mathit{H_3}$). LRMS (CI) *m/z* calcd for $C_{17}H_{16}ClN_4O_3$ $[M+H]^+$: 359.1; found: 359.1.

(±) Isopropyl 7-chloro-2-methyl-4-(oxazol-4-yl)-1,4-dihydrobenzo[4,5]imidazo[1,2-*a*]pyrimidine-3-carboxylate (17d). Purified by column chromatography (*n*-Hexane–Ethyl Acetate 1:1) to give 30 mg, 13%. Mp: 247 – 248 ºC. ^{1}H NMR (300 MHz, $CDCl_3$), δ (ppm): 8.00 – 7.60 (m, 2H, Ar-H), 7.47 – 7.23 (m, 2H, Ar-H), 7.19 – 7.05 (m, 1H, Ar-H), 6.55 (s, 1H, CH), 5.20 – 5.00 (m, 1H, $OC\mathit{H}(CH_3)_2$), 2.69 (s, 3H, CH_3), 1.34 (d, J = 6.1 Hz, 3H, $OCH(C\mathit{H_3})_2$), 1.22 (d, J = 6.1 Hz, 3H, $OCH(C\mathit{H_3})_2$). LRMS (CI) *m/z* calcd for $C_{18}H_{18}ClN_4O_3$ $[M+H]^+$: 373.1; found: 373.1.

(±) Ethyl 8-fluoro-2-methyl-4-(oxazol-4-yl)-1,4-dihydrobenzo[4,5]imidazo[1,2-*a*]pyrimidine-3-carboxylate (18a). Purified by column chromatography (*n*-Hexane–Ethyl Acetate 1:1) to give 26 mg, 12%. Mp: 247 – 249 ºC. ^{1}H NMR (300 MHz, $CDCl_3$), δ (ppm): 7.77 – 7.67 (m, 2H, Ar-H), 7.33 – 7.23 (m, 1H, Ar-H), 7.22 – 7.16 (m, 1H, Ar-H), 6.92 – 6.78 (m, 1H, Ar-H), 6.58 (s, 1H, CH), 4.24 (q, J = 7.1, 2H, $OC\mathit{H_2}CH_3$), 2.72 (s, 3H, CH_3), 1.31 (t, J = 7.1 Hz, 3H, $OCH_2C\mathit{H_3}$). LRMS (CI) *m/z* calcd for $C_{17}H_{16}FN_4O_3$ $[M+H]^+$: 343.1; found: 343.2.

(±) Isopropyl 8-fluoro-2-methyl-4-(oxazol-4-yl)-1,4-dihydrobenzo[4,5]imidazo[1,2-*a*]pyrimidine-3-carboxylate (18b). Purified by column chromatography (*n*-Hexane–Ethyl Acetate 1:1) to give 27 mg, 12%. Mp: 255 – 256 ºC. ^{1}H NMR (300 MHz, $CDCl_3$), δ (ppm): 7.86 – 7.65 (m, 2H, Ar-H), 7.49 – 7.05 (m, 2H, Ar-H), 7.05 – 6.77 (m, 1H, Ar-H), 6.54 (d, J = 12.3 Hz, 1H, CH), 5.11 (p, J = 6.3 Hz, 1H, OC*H*(CH_3)$_2$), 2.73 (s, 3H, CH_3), 1.33 (d, J = 6.3 Hz, 3H, OCH(C*H$_3$*)$_2$), 1.23 (d, J = 6.2 Hz, 3H, OCH(C*H$_3$*)$_2$). LRMS (CI) *m/z* calcd for $C_{18}H_{18}FN_4O_3$ [M+H]$^+$: 357.1; found: 357.4

(±) Ethyl 8-chloro-2-methyl-4-(oxazol-4-yl)-1,4-dihydrobenzo[4,5]imidazo[1,2-*a*]pyrimidine-3-carboxylate (18c). Purified by column chromatography (*n*-Hexane–Ethyl Acetate 1:1) to give 20 mg, 11%. Mp: 250 – 252 ºC. ^{1}H NMR (300 MHz, $CDCl_3$), δ (ppm): 7.79 – 7.68 (m, 2H, Ar-H), 7.49 (d, J = 8.1 Hz, 1H, Ar-H), 7.44 – 7.23 (m, 1H, Ar-H), 7.12 (d, J = 8.1 Hz, 1H, Ar-H), 6.57 (s, 1H, CH), 4.24 (q, J = 7.1 Hz, 2H, OC*H$_2$*CH_3), 2.72 (s, 3H, CH_3), 1.33 (t, J = 7.1 Hz, 3H, OCH_2C*H$_3$*). HRMS (APCI) *m/z*: calcd for $C_{17}H_{15}ClN_4O_3$ [M+H]$^+$: 359.0833; found: 359.0833.

(*R*) Ethyl 8-chloro-2-methyl-4-(oxazol-4-yl)-1,4-dihydrobenzo[4,5]imidazo[1,2-*a*]pyrimidine-3-carboxylate [(*R*)-18c]. Chiral separation with a 250 mm x 20 mm Chiralpak® 5µm IE-3 (DAICEL) (isocratic mobile phase Dichloromethane-*i-Propanol* 7:3) to give an enantiomeric excess of >99%. ^{1}H NMR (300 MHz, $CDCl_3$), δ (ppm): 7.80 – 7.68 (m, 2H, Ar-H), 7.48 (d, J = 8.1 Hz, 1H, Ar-H), 7.40 – 7.23 (m, 1H, Ar-H), 7.12 (d, J = 8.1 Hz, 1H, Ar-H), 6.56 (s, 1H, CH), 4.23 (q, J = 7.1 Hz, 2H, OC*H$_2$*CH_3), 2.73 (s, 3H, CH_3), 1.32 (t, J = 7.1 Hz, 3H, OCH_2C*H$_3$*).

(*S*) Ethyl 8-chloro-2-methyl-4-(oxazol-4-yl)-1,4-dihydrobenzo[4,5]imidazo[1,2-*a*]pyrimidine-3-carboxylate [(*S*)-18c]. Chiral separation with a 250 mm x 20 mm Chiralpak® 5µm IE-3 (DAICEL) (isocratic mobile phase Dichloromethane-*i-Propanol* 7:3) to give an enantiomeric excess of >99%. ^{1}H NMR (300 MHz, $CDCl_3$), δ (ppm): 7.80 – 7.71 (m, 2H, Ar-H), 7.48 (d, J = 8.0 Hz, 1H, Ar-H), 7.42 – 7.21 (m, 1H, Ar-H), 7.12 (d, J = 8.0 Hz, 1H, Ar-H), 6.51 (s, 1H, CH), 4.24 (q, J = 7.2 Hz, 2H, OC*H$_2$*CH_3), 2.73 (s, 3H, CH_3), 1.35 (t, J = 7.2 Hz, 3H, OCH_2C*H$_3$*).

(±) Isopropyl 8-chloro-2-methyl-4-(oxazol-4-yl)-1,4-dihydrobenzo[4,5]imidazo[1,2-*a*]pyrimidine-3-carboxylate (18d). Purified by column chromatography (*n*-Hexane–Ethyl Acetate 1:1) to give 39 mg, 20%. Mp: 244 – 246 ºC. ^{1}H NMR (300 MHz, $CDCl_3$), δ (ppm): 8.00 – 7.60 (m, 2H, Ar-H), 7.47 (d, J = 1.8 Hz, 1H, Ar-H), 7.37 – 7.23 (m, 1H, Ar-H), 7.12 (dd, J = 8.5, 1.8 Hz, 1H, Ar-H), 6.55 (s, 1H, CH), 5.12 (p, J = 6.2 Hz, 1H, OC*H*(CH_3)$_2$), 2.70 (s, 3H, CH_3), 1.33 (d, J = 6.2 Hz, 3H, OCH(C*H$_3$*)$_2$), 1.24 (d, J = 6.2 Hz, 3H, OCH(C*H$_3$*)$_2$). LRMS (CI) *m/z* calcd for $C_{18}H_{18}ClN_4O_3$ [M+H]$^+$: 373.1; found: 373.1

HPLC enantiomeric separation and characterization of ISAM-163 (16b), ISAM-161 (16j), ISAM-M89A (16l), ISAM-157 (16r), ISAM-M114A (16s) and ISAM-R316A (18c).

The chiral resolution was performed using a Water Breeze™ 2 (binary pump 1525, detector UV/Visible 2489, 7725i Manual Injector Kit 1500 Series). Compound **16b** enantiomers were separated using a 250 mm x 10 mm Lux® 5 µm Amylose-2 (Phenomenex) while **16j, 16l, 16r**, **16s** and **18c** enantiomers were separated using a 250 mm x 20 mm Chiralpak® 5µm IE-3 (DAICEL). All the separations were performed at 25 ºC with *n*-hexane (or dichloromethane)/*i*-propanol mixture in different percentages as mobile phase (*see spectroscopic and analytical data*). The enantiomers [**(*R*)-16b** (0.5 mg, t_R = 9.08 min), **(*S*)-16b** (0.5 mg, t_R = 13.64 min), **(*R*)-16j** (0.5 mg, t_R = 16.96 min), **(*S*)-16j** (0.5 mg, t_R = 20.32 min), **(*S*)-16l** (0.5 mg, t_R = 14.43 min), **(*R*)-16l** (0.5 mg, t_R = 15.67 min), **(*R*)-16r** (0.5 mg, t_R = 17.44 min), **(*S*)-16r** (0.5 mg, t_R = 20.04 min), **(*S*)-16s** (0.5 mg, t_R = 31.02 min), **(*R*)-16s** (0.5 mg, t_R = 36.61 min), **(*S*)-18c** (0.5 mg, t_R = 6.99 min) and **(*R*)-18c** (0.5 mg, t_R = 7.53 min)] were isolated, their stereochemical purity analyzed by chiral HPLC (ee: 97-99% for each enantiomer, reports showed below, chromatogram at 254 nM) and then characterized by ^{1}H NMR and Circular dichroism (CD).

CD spectra were recorded on a Jasco-815 system equipped with a Peltier-type thermostatic accessory (CDF-426S, Jasco). Measurements were carried out at 20 °C using a 1 mm quartz cell in a volume of 600 μL. Compounds (0.5 mg) were dissolved in MeOH (1.0 mL) and then diluted 10-fld in MeOH. The instrument settings were bandwidth, 1.0 nm; data pitch, 1.0 nm; speed, 500 nm/min; accumulation, 10; wavelengths, 400−190 nm.

SAMPLE INFORMATION

Sample Name:	ISAM-163	Acquired By:	Breeze
Sample Type:	Racemate	Sample Set Name:	Halogenated
Vial:	1	Acq. Method:	10%HexiPAISOF25 set
Injection #:	1	Date Acquired:	05/11/2019 11:09:20 CET
Run Time:	25,00 Minutes	Injection Volume:	200,00 ul

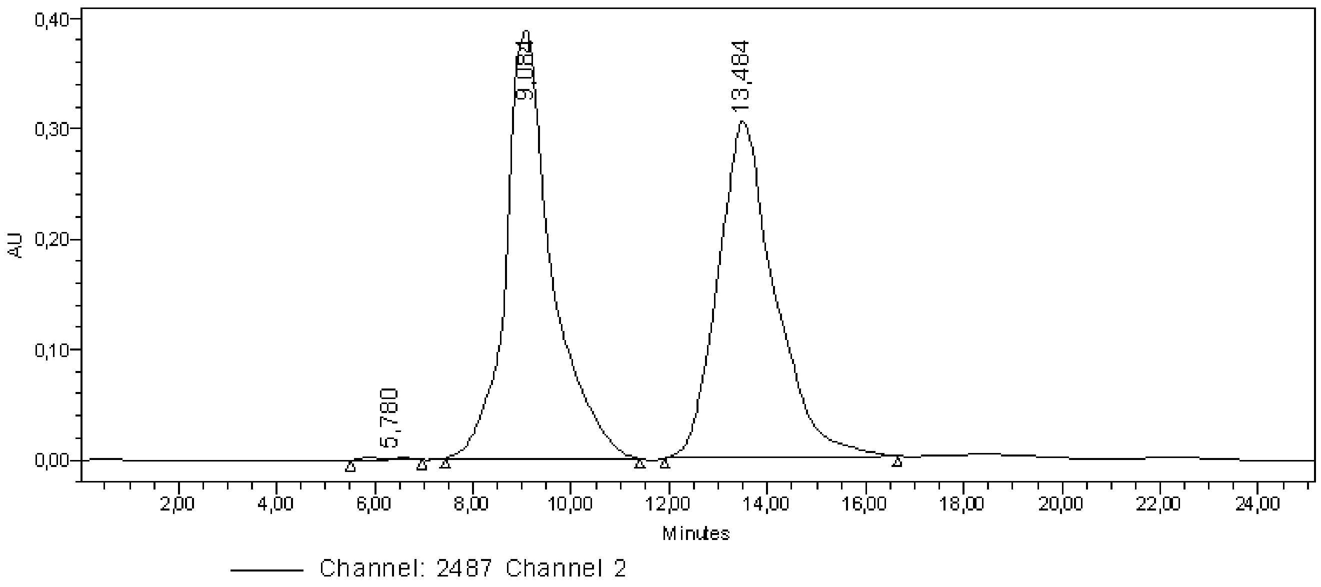

	RT (min)	Area (µV*sec)	% Area	Height (µV)
1	5,780	130009	0,22	2408
2	9,084	29905180	51,44	381108
3	13,484	28103184	48,34	287862

SAMPLE INFORMATION

Sample Name:	ISAM-163A	Acquired By:	Breeze
Sample Type:	Enantiomer A	Sample Set Name:	Halogenated
Vial:	1	Acq. Method:	10%HexiPAISOF25 set
Injection #:	2	Date Acquired:	05/11/2019 11:35:31 CET
Run Time:	25,00 Minutes	Injection Volume:	200,00 ul

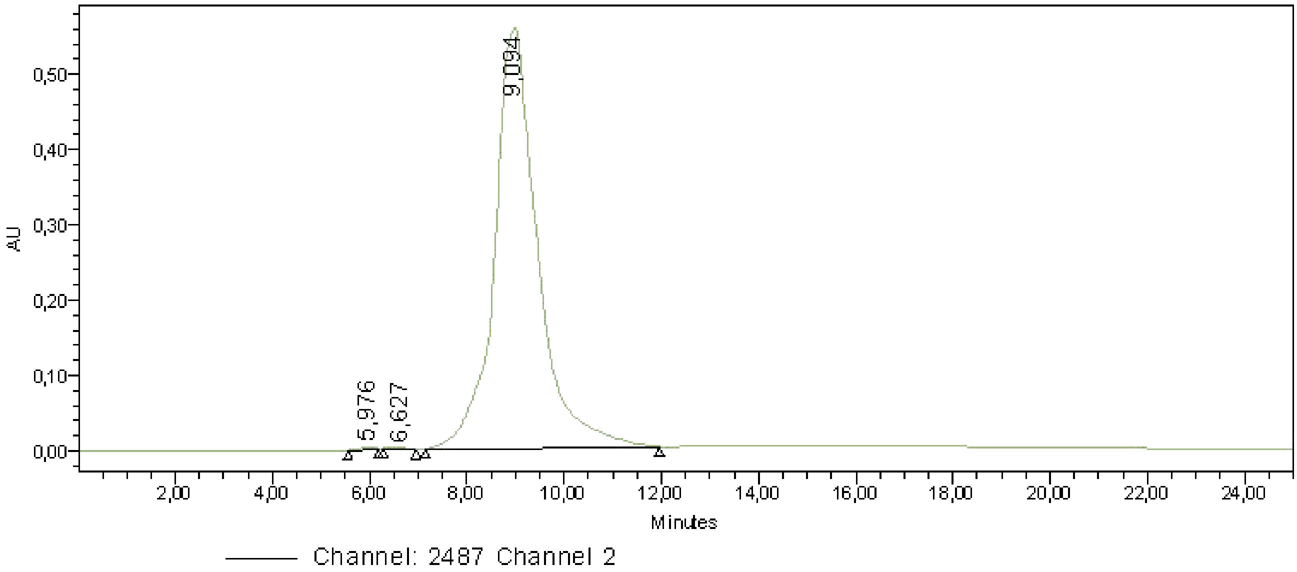

	RT (min)	Area (µV*sec)	% Area	Height (µV)
1	5,976	57365	0,13	2367
2	6,627	27869	0,06	1366
3	9,094	44924077	99,81	560062

SAMPLE INFORMATION

Sample Name:	ISAM-163B	Acquired By:	Breeze
Sample Type:	Enantiomer B	Sample Set Name:	Halogenated
Vial:	1	Acq. Method:	10%HexiPAISOF25 set
Injection #:	3	Date Acquired:	05/11/2019 12:01:34 CET
Run Time:	25,00 Minutes	Injection Volume:	200,00 ul

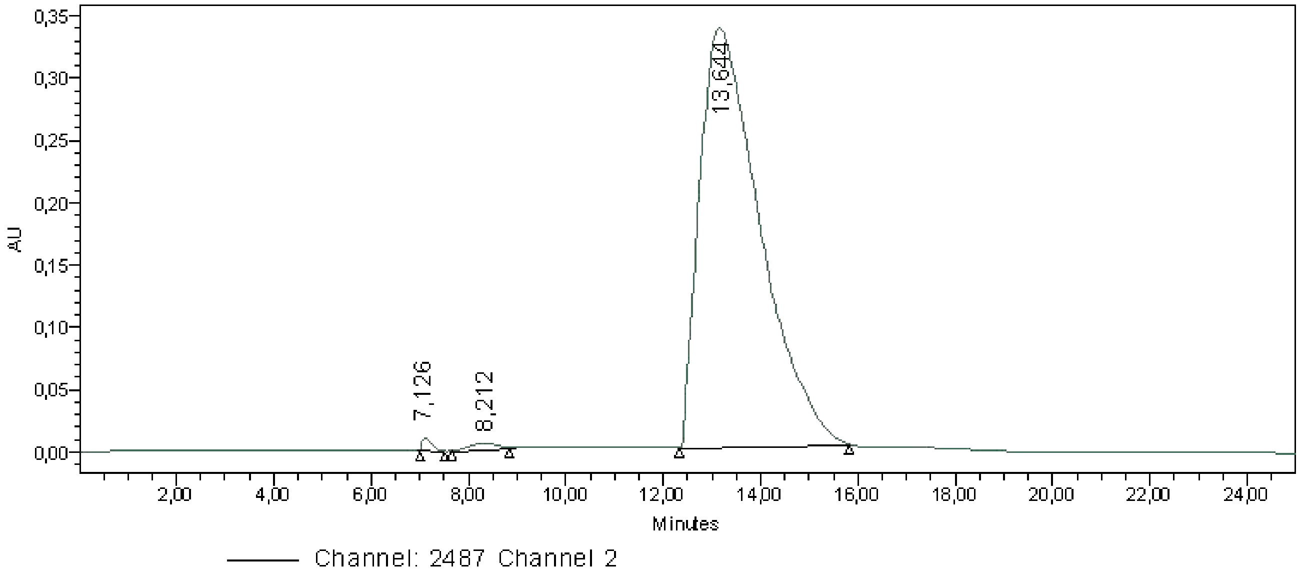

	RT (min)	Area (µV*sec)	% Area	Height (µV)
1	7,126	250627	0,77	8141
2	8,212	550257	1,31	11111
3	13,644	31887052	97,92	335299

SAMPLE INFORMATION

Sample Name:	ISAM-161	Acquired By:	Breeze
Sample Type:	Racemate	Sample Set Name:	Halogenated
Vial:	1	Acq. Method:	10%HexiPAISOF10 set
Injection #:	1	Date Acquired:	12/11/2019 10:15:13 CET
Run Time:	40,00 Minutes	Injection Volume:	200,00 ul

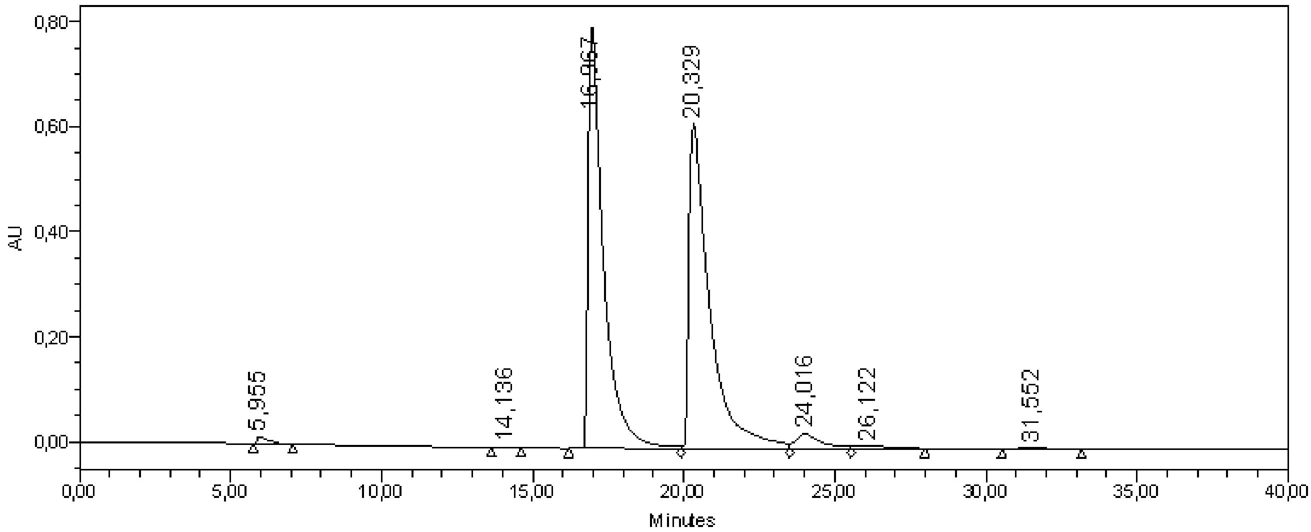

Channel: 2487 Channel 2

	RT (min)	Area (µV*sec)	% Area	Height (µV)
1	5,955	346793	0,57	13474
2	14,136	43149	0,07	1704
3	16,967	28522694	47,20	801999
4	20,329	29445809	48,73	617995
5	24,016	1563809	2,59	27390
6	26,122	363781	0,60	4388
7	31,552	143889	0,24	1784

SAMPLE INFORMATION

Sample Name:	ISAM-161	Acquired By:	Breeze
Sample Type:	Enantiomer A	Sample Set Name:	Halogenated
Vial:	1	Acq. Method:	10%HexiPAISOF10 set
Injection #:	2	Date Acquired:	12/11/2019 11:08:15 CET
Run Time:	40,00 Minutes	Injection Volume:	200,00 ul

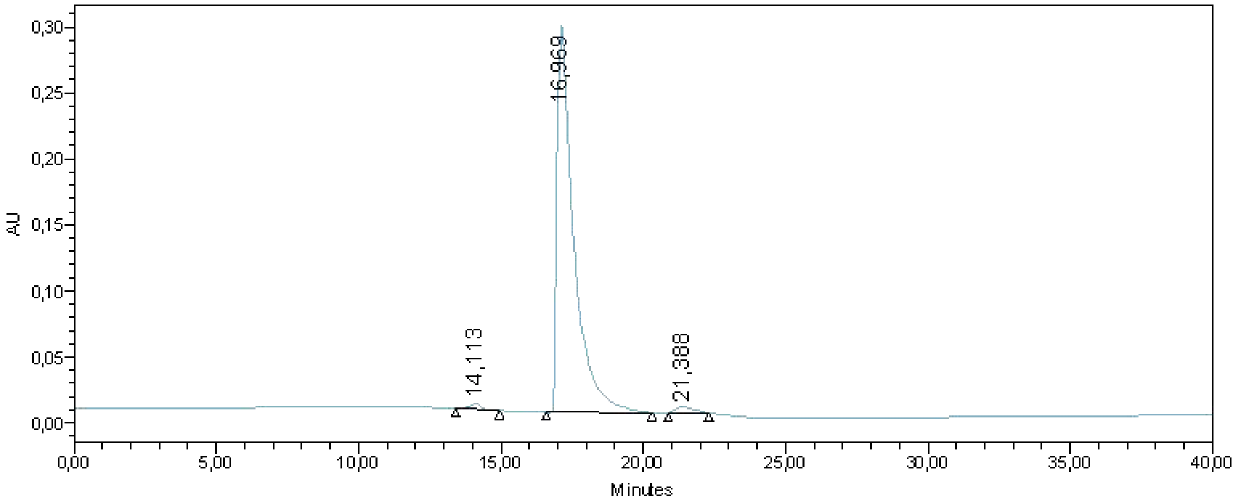

Channel: 2487 Channel 2

	RT (min)	Area (µV*sec)	% Area	Height (µV)
1	14,113	122523	1,00	4616
2	16,969	11913486	97,24	293326
3	21,388	215723	1,76	4720

SAMPLE INFORMATION

Sample Name:	ISAM-161	Acquired By:	Breeze
Sample Type:	Enantiomer B	Sample Set Name:	Halogenated
Vial:	1	Acq. Method:	10%HexiPAISOF10 set
Injection #:	3	Date Acquired:	12/11/2019 12:37:53 CET
Run Time:	40,00 Minutes	Injection Volume:	200,00 ul

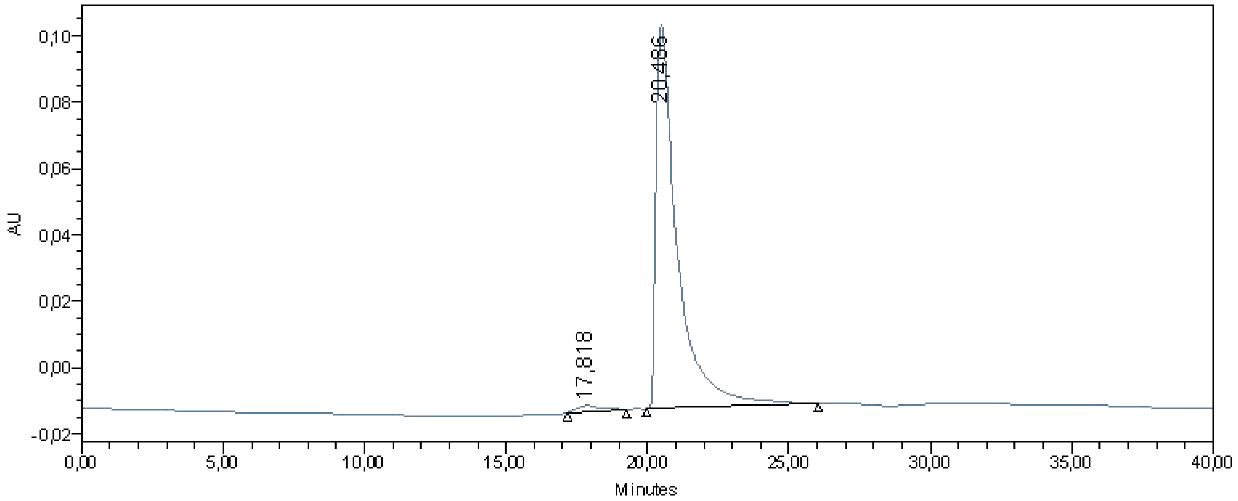

Channel: 2487 Channel 2

	RT (min)	Area (µV*sec)	% Area	Height (µV)
1	17,818	86535	1,39	1483
2	20,486	6124545	98,61	115233

SAMPLE INFORMATION

Sample Name:	ISAM-M89A rac analysis	Acquired By:	Breeze
Sample Type:	Unknown	Sample Set Name	
Vial:	1	Acq. Method:	20%HexiPAISOF10 set
Injection #:	1	Date Acquired:	22/03/2022 10:31:55 CET
Run Time:	30,00 Minutes	Injection Volume:	250,00 ul

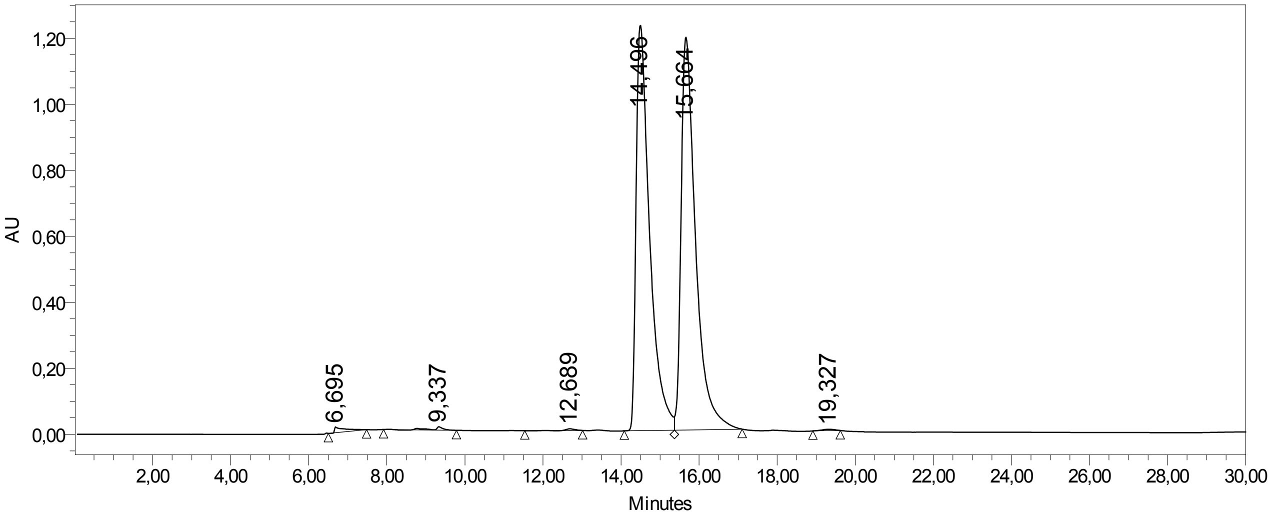

Channel: 2487Channel 2; Channel Desc.: ; Processing Method: AAA

	RT (min)	Area (µV*sec)	% Area	Height (µV)
1	6,695	331828	0,54	17701
2	9,337	221427	0,36	10075
3	12,689	99422	0,16	5023
4	14,496	29629697	48,52	1228531
5	15,664	30685711	50,25	1190205
6	19,327	95227	0,16	4180

SAMPLE INFORMATION

Sample Name:	ISAM-M89A E1 analysis	Acquired By:	Breeze
Sample Type:	Unknown	Sample Set Name	
Vial:	1	Acq. Method:	20%HexiPAISOF10 set
Injection #:	2	Date Acquired:	22/03/2022 11:17:56 CET
Run Time:	30,00 Minutes	Injection Volume:	250,00 ul

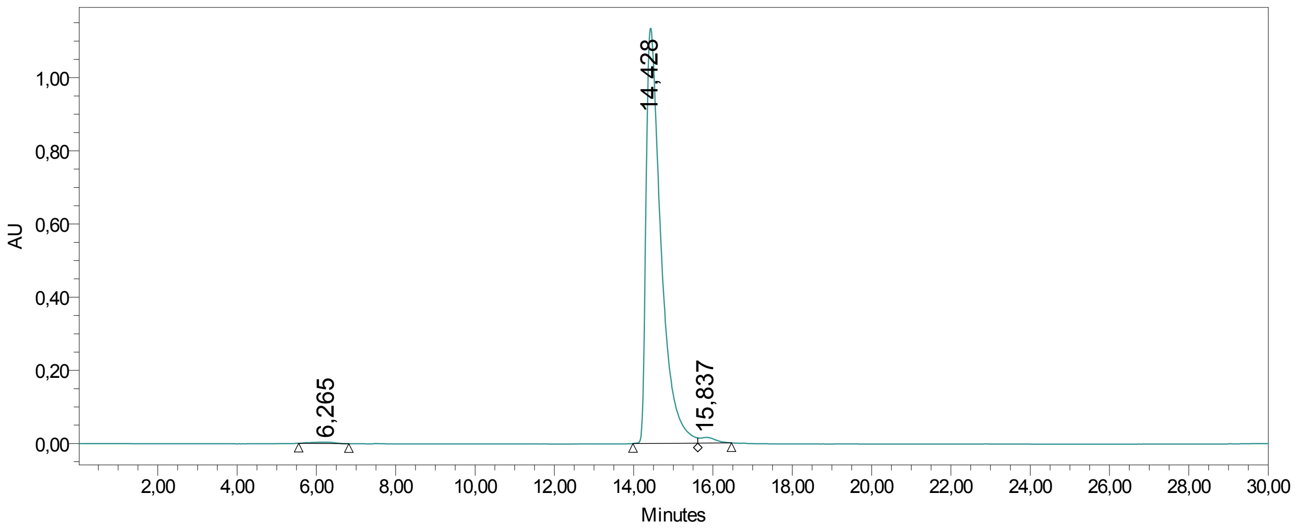

Channel: 2487Channel 2; Channel Desc.: ; Processing Method: AAA

	RT (min)	Area (µV*sec)	% Area	Height (µV)
1	6,265	153541	0,51	3538
2	14,428	29751250	98,05	1135412
3	15,837	438757	1,45	15317

SAMPLE INFORMATION

Sample Name:	ISAM-M89A E2 analysis	Acquired By:	Breeze
Sample Type:	Unknown	Sample Set Name	
Vial:	1	Acq. Method:	20%HexiPAISOF10 set
Injection #:	3	Date Acquired:	22/03/2022 11:51:40 CET
Run Time:	30,00 Minutes	Injection Volume:	250,00 ul

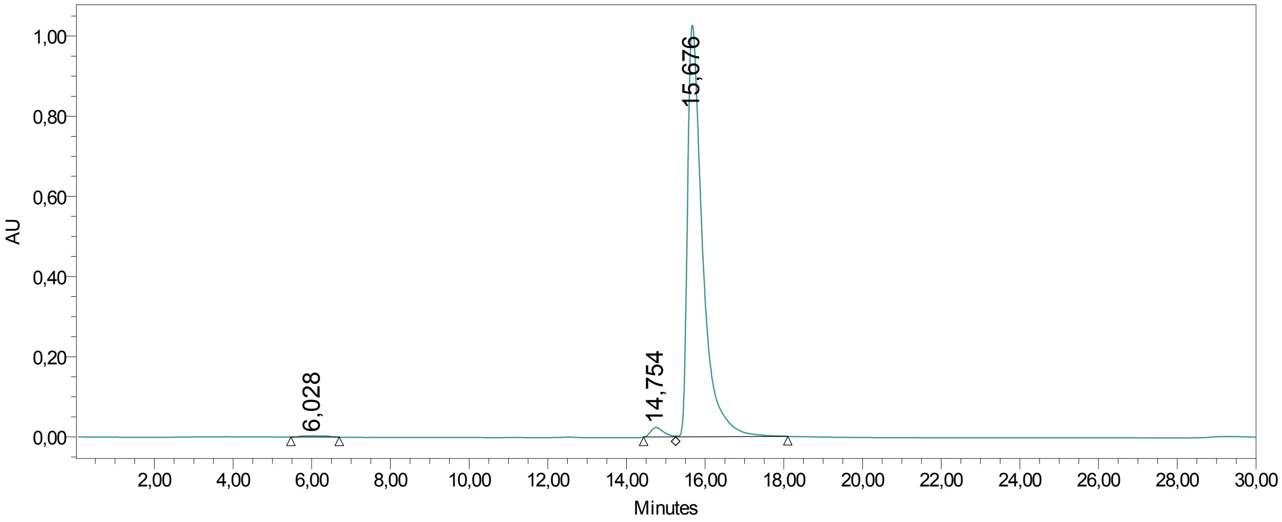

Channel: 2487Channel 2; Channel Desc.: ; Processing Method: AAAAAA

	RT (min)	Area (µV*sec)	% Area	Height (µV)
1	6,028	131923	0,47	3053
2	14,754	552450	1,98	23908
3	15,676	27279141	97,55	1027996

SAMPLE INFORMATION

Sample Name:	ISAM-157	Acquired By:	Breeze
Sample Type:	Racemate	Sample Set Name:	Rubén Prieto
Vial:	1	Acq. Method:	10%HexiPAISOF10 set
Injection #:	1	Date Acquired:	12/11/2019 16:12:28 CET
Run Time:	30,00 Minutes	Injection Volume:	200,00 ul

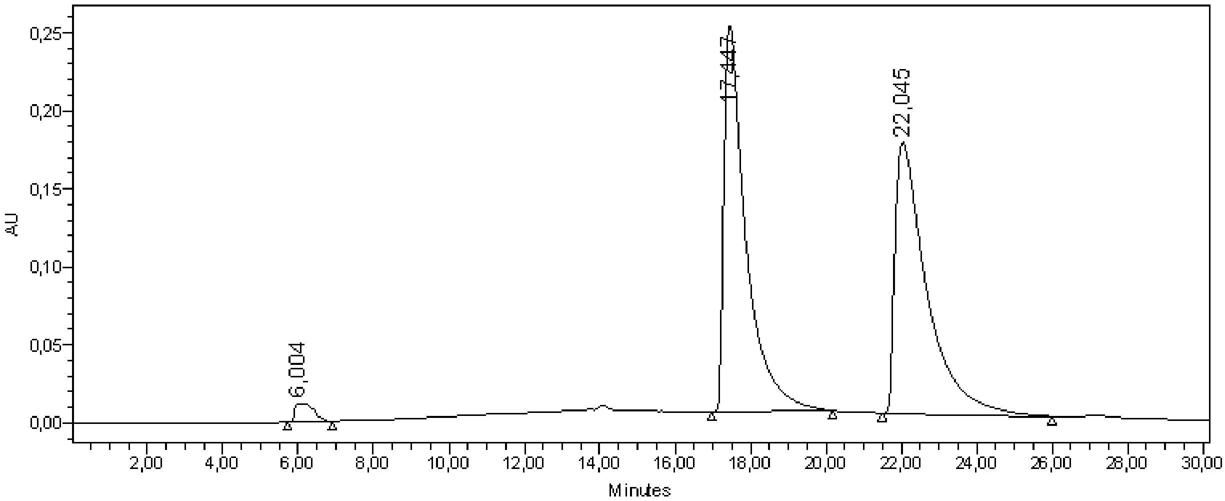

Channel: 2487 Channel 1

	RT (min)	Area (µV*sec)	% Area	Height (µV)
1	6,004	389275	1,88	11558
2	17,447	10137209	49,05	247407
3	22,045	10142524	49,07	173791

SAMPLE INFORMATION

Sample Name:	ISAM-157	Acquired By:	Breeze
Sample Type:	Enantiomer A	Sample Set Name:	Halogenated
Vial:	1	Acq. Method:	10%HexiPAISOF10 set
Injection #:	2	Date Acquired:	12/11/2019 16:43:55 CET
Run Time:	30,00 Minutes	Injection Volume:	200,00 ul

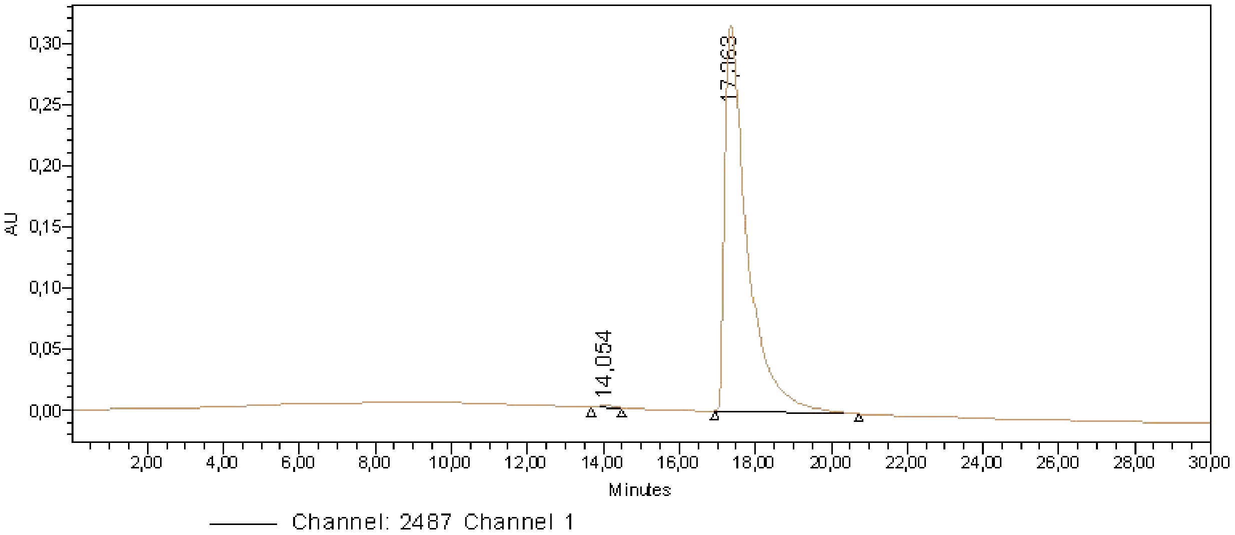

	RT (min)	Area (µV*sec)	% Area	Height (µV)
1	14,054	36505	0,28	2158
2	17,363	13014953	99,72	315685

SAMPLE INFORMATION

Sample Name:	ISAM-157	Acquired By:	Breeze
Sample Type:	Enantiomer B	Sample Set Name:	Halogenated
Vial:	1	Acq. Method:	10%HexiPAISOF10 set
Injection #:	3	Date Acquired:	12/11/2019 17:14:39 CET
Run Time:	30,00 Minutes	Injection Volume:	200,00 ul

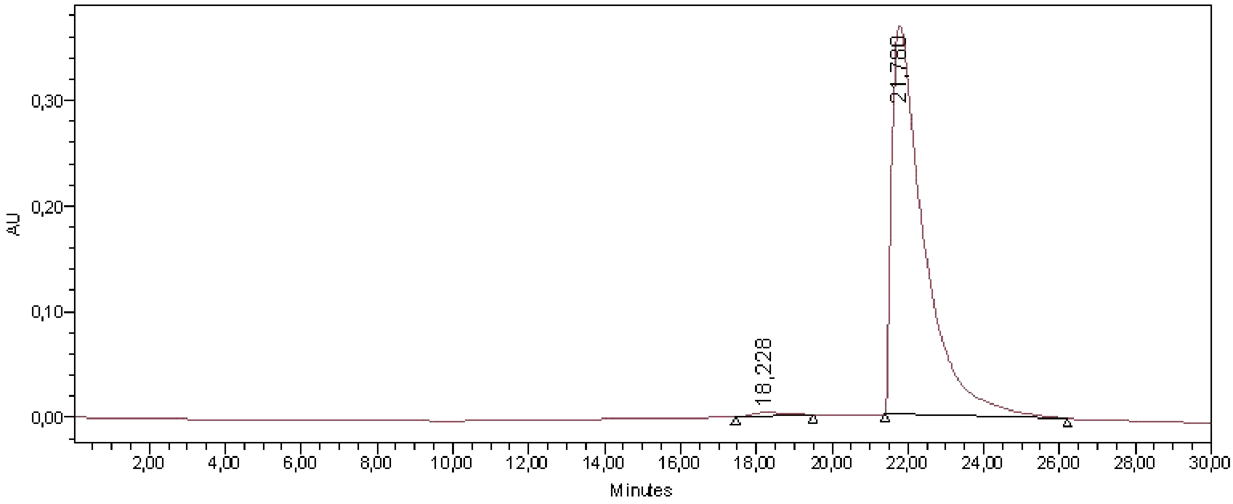

Channel: 2487 Channel 1

	RT (min)	Area (µV*sec)	% Area	Height (µV)
1	18,228	202867	0,93	3354
2	21,780	21518158	99,07	367510

S A M P L E   I N F O R M A T I O N

Sample Name:	ISAM-M114A	Acquired By:	Breeze
Sample Type:	Racemate	Sample Set Name:	Halogenated
Vial:	1	Acq. Method:	20%HexiPAISOF10 set
Injection #:	1	Date Acquired:	13/11/2019 18:11:30 CET
Run Time:	60,00 Minutes	Injection Volume:	200,00 ul

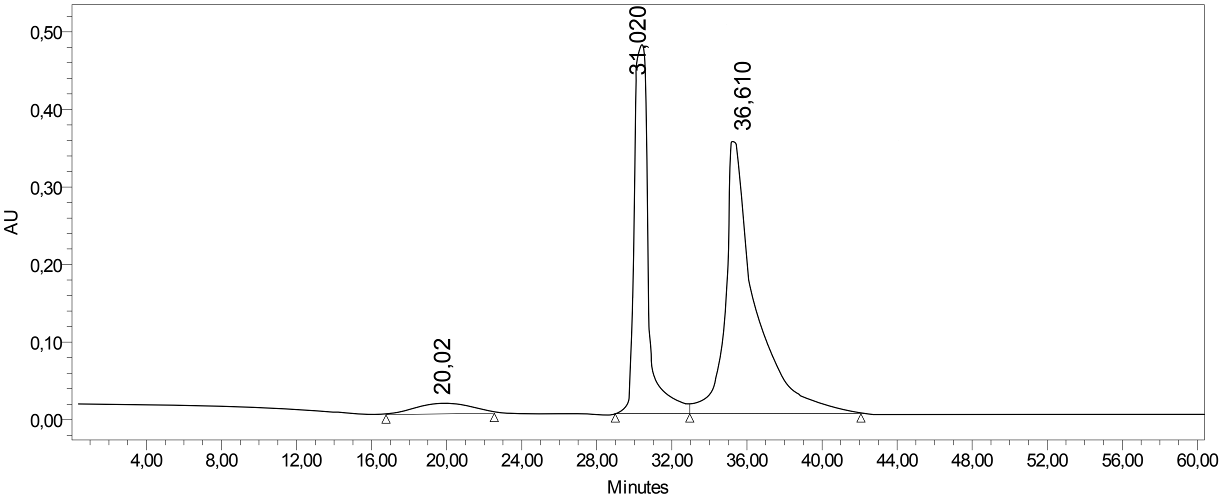

Channel: 2487 Channel 1

	RT (min)	Area (µV*sec)	% Area	Height (µV)
1	20,02	248921	1,20	23116
2	31,020	20274418	49,35	494814
3	36,610	20285048	49,45	347582

SAMPLE INFORMATION

Sample Name:	ISAM-M114A	Acquired By:	Breeze
Sample Type:	Enantiomer A	Sample Set Name:	Halogenated
Vial:	1	Acq. Method:	20%HexiPAISOF10 set
Injection #:	1	Date Acquired:	13/11/2019 19:15:22 CET
Run Time:	60,00 Minutes	Injection Volume:	200,00 ul

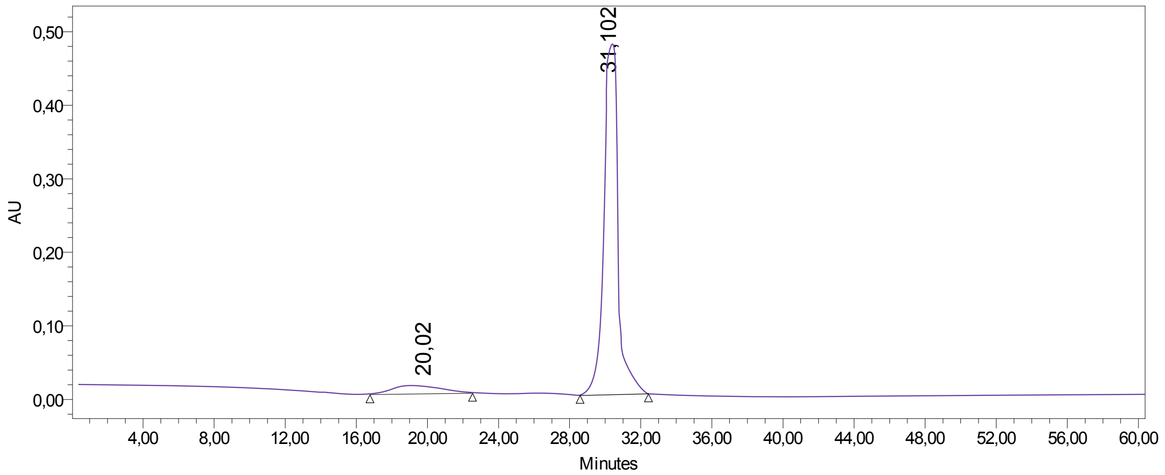

Channel: 2487 Channel 1

	RT (min)	Area (µV*sec)	% Area	Height (µV)
1	20,020	127111	0,44	1661
2	31,102	27819287	99,56	347811

SAMPLE INFORMATION

Sample Name:	ISAM-M114A	Acquired By:	Breeze
Sample Type:	Enantiomer B	Sample Set Name:	Halogenated
Vial:	1	Acq. Method:	20%HexiPAISOF10 set
Injection #:	1	Date Acquired:	13/11/2019 20:11:11 CET
Run Time:	60,00 Minutes	Injection Volume:	200,00 ul

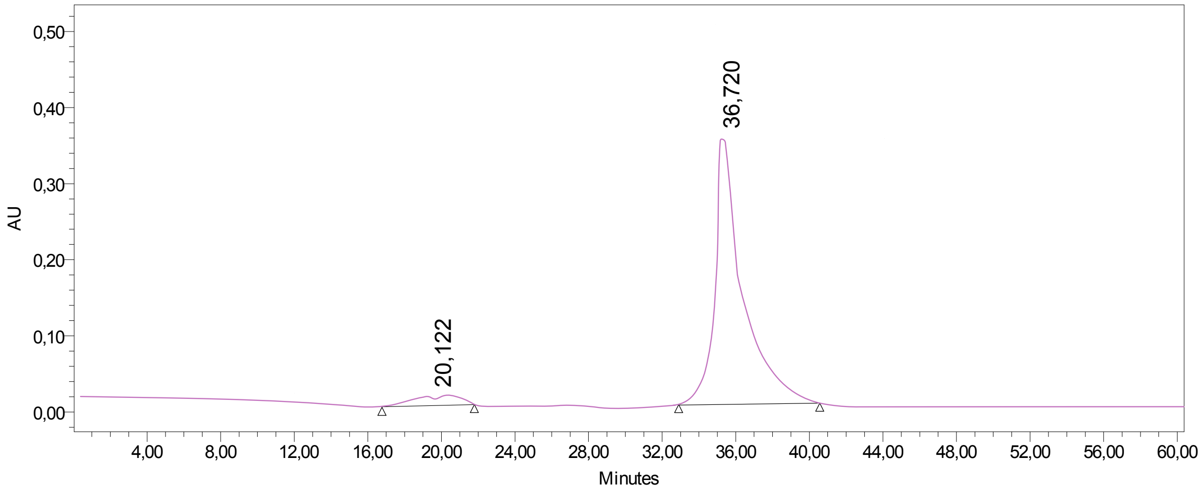

Channel: 2487 Channel 1

	RT (min)	Area (µV*sec)	% Area	Height (µV)
1	20,122	294666	1,02	3421
2	36,720	28594221	98,98	440192

SAMPLE INFORMATION			
Sample Name:	ISAM-R316A	Acquired By:	Breeze
Sample Type:	Racemate	Sample Set Name	
Vial:	2	Acq. Method:	30%DCMiPAISOF10 set
Injection #:	5	Date Acquired:	18/01/2022 16:39:27 CET
Run Time:	12,00 Minutes	Injection Volume:	500,00 ul

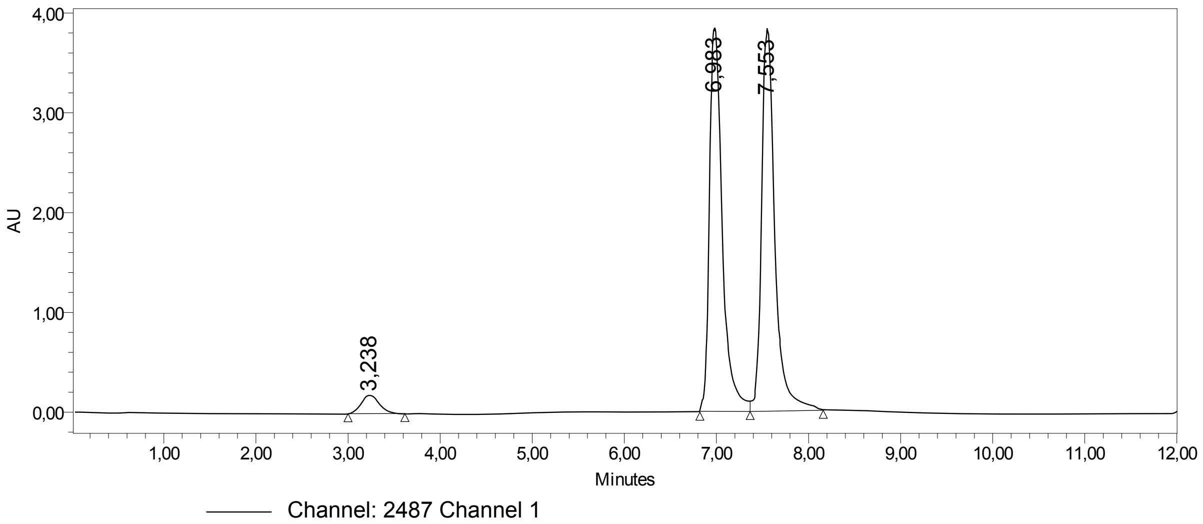

	RT (min)	Area (µV*sec)	% Area	Height (µV)
1	3,238	2470910	3,52	183100
2	6,983	33585027	47,89	3758787
3	7,553	34067111	48,58	3706311

SAMPLE INFORMATION

Sample Name:	ISAM-R316A E1	Acquired By:	Breeze
Sample Type:	Enantiomer A	Sample Set Name	
Vial:	1	Acq. Method:	30%DCMiPAISOF10 set
Injection #:	1	Date Acquired:	18/01/2022 10:25:33 CET
Run Time:	12,00 Minutes	Injection Volume:	500,00 ul

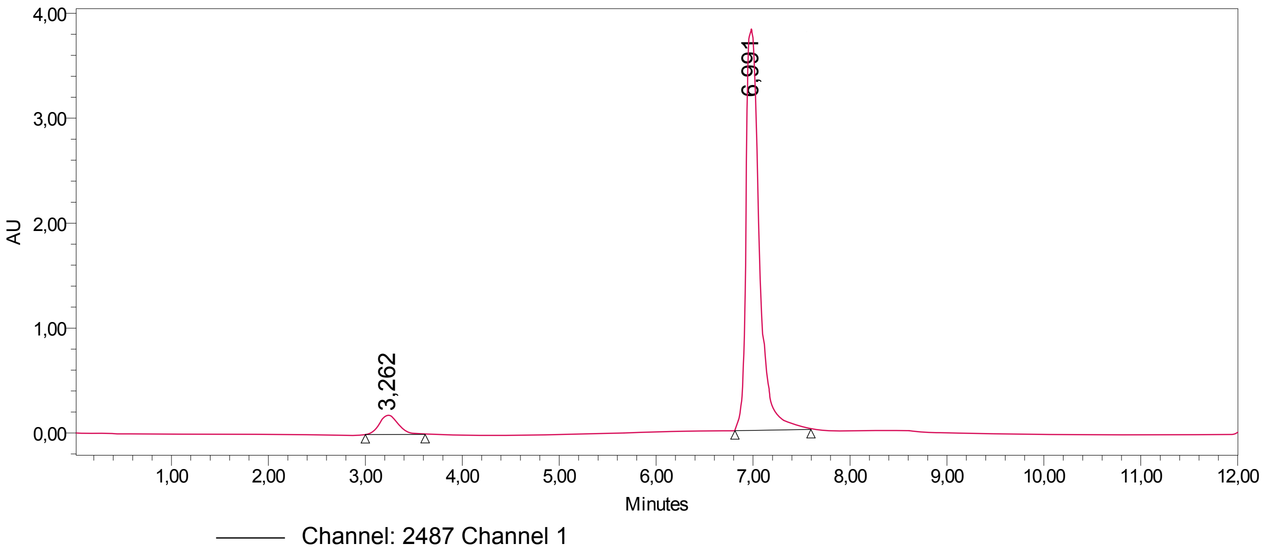

	RT (min)	Area (µV*sec)	% Area	Height (µV)
1	3,262	1254747	3,48	182982
2	6,991	34801190	96,52	3758562

SAMPLE INFORMATION

Sample Name:	ISAM-R316A E2	Acquired By:	Breeze
Sample Type:	Enantiomer B	Sample Set Name	
Vial:	3	Acq. Method:	30%DCMiPAISOF10 set
Injection #:	3	Date Acquired:	18/01/2022 11:22:21 CET
Run Time:	12,00 Minutes	Injection Volume:	500,00 ul

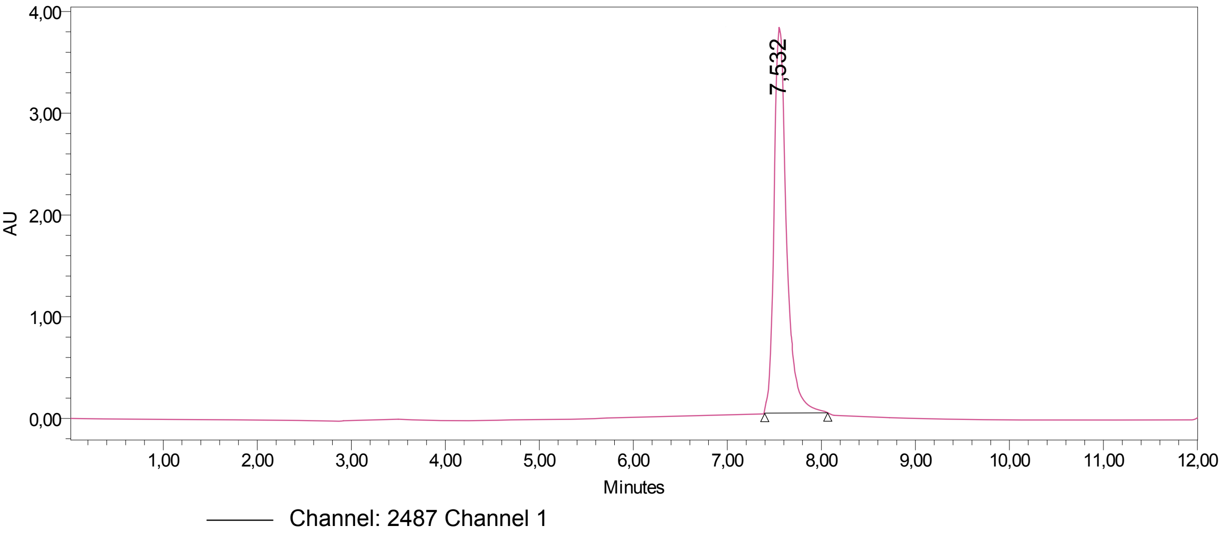

	RT (min)	Area (µV*sec)	% Area	Height (µV)
1	7,532	34801190	100,0	3824892

X-ray crystallography of compounds 16s (*S*)-ISAM-M114A and (*R*)-ISAM-M114A

Crystals of **(*S*)-ISAM-M114A** and **(*R*)-ISAM-M114A** were grown by slow evaporation from ethanol solutions. For the crystal structure determination, the data were collected by applying the omega and phi scans method on a Bruker D8 VENTURE PHOTON III-14 diffractometer using Incoatec multilayer mirror monochromated with Cu-Kα radiation (λ = 1.54178 Å) from a microfocus sealed tube source at 100 K with detector resolution of 7.3910 pixels mm-1. Computing data and reduction were made with the APEX3 v2018.7-2 (BRUKER AXS, 2005). The structure was solved using SHELXT2018/22 and finally refined by full-matrix least-squares based on F2 by SHELXL2018/3.3 An empirical absorption correction was applied using the SADABS2016/2 program. Software used to molecular graphics: ORTEP for Windows. Software used to prepare material for publication: WinGX2018.3 publication routines4 and Mercury.

Detailed crystallographic data for ligands **(*S*)-ISAM-M114A** and **I-ISAM-M114A** have been deposited at the Cambridge Crystallographic Data Centre (deposition number CCDC: 2047365, 2048281) and are available on request.

Refinement

All non-hydrogen atoms were refined anisotropically, and the hydrogen atom positions were included in the model based on Fourier difference electron density maps. All aromatic CH hydrogen (C-H = 0.95 Å), methine hydrogen (C-H = 1.0 Å) and methylene hydrogen (C-H=0.99 Å) atoms were refined using a riding model with Uiso(H) = 1.2 UeqI. The methyl hydrogen (C-H = 0.98 Å) atoms were refined as a rigid group with torsional freedom [Uiso(H) = 1.5 UeqI] and the hydrogens atom of NH groups (HiN) as a free atom with Uiso(H) = 1.2 UeqI. Details of the experiment shown in the table below.

Crystal data	(*S*)-ISAM-M114A	I-ISAM-M114A
**CCDC**	2047365	2048281
Chemical formula	$2(C_{18}H_{16}BrN_3O_3)\cdot C_3H_8O$	$2(C_{18}H_{16}BrN_3O_3)\cdot C_3H_8O$
M_r	864.59	337.37
Crystal system	Triclinic	Triclinic
Space group	P1	P1
Temperature (K)	100	100
a (Å)	8.1434 (1)	8.1451 (1)
b (Å)	10.0812 (2)	10.0886 (1)
c (Å)	11.7595 (2)	11.7642 (2)
α (°)	99.2705 (9)	99.2342 (6)
β (°)	98.3976 (9)	98.4614 (6)
γ (°)	99.2899 (8)	99.3197 (6)
V ($Å^3$)	925.51 (3)	926.58 (2)
Z	1	1
Radiation type	Cu-Kα	Cu-Kα
μ (mm^{-1})	3.27	3.27
Crystal size (mm)	0.09 × 0.06 × 0.05	0.20 × 0.14 × 0.06
Tmin, Tmax	0.740, 0.850	0.600, 0.730
Measured/Independent/ observed [I>2σ(I)reflection	42137/7470/6822	67154/7500/7331
Rint	0.058	0.042
$R[F^2>2\sigma(F^2)], wR(F2), S$	0.041, 0.102, 1.11	0.022, 0.057, 1.04
$\Delta\rho max/\Delta\rho min$ ($eÅ^{-3}$)	0.38, −0.57	0.38, -0.31
Absolute structure (Flack)	-0.017 (18)	-0.031 (8)

Supplementary Figures and Tables

Supplementary Figures

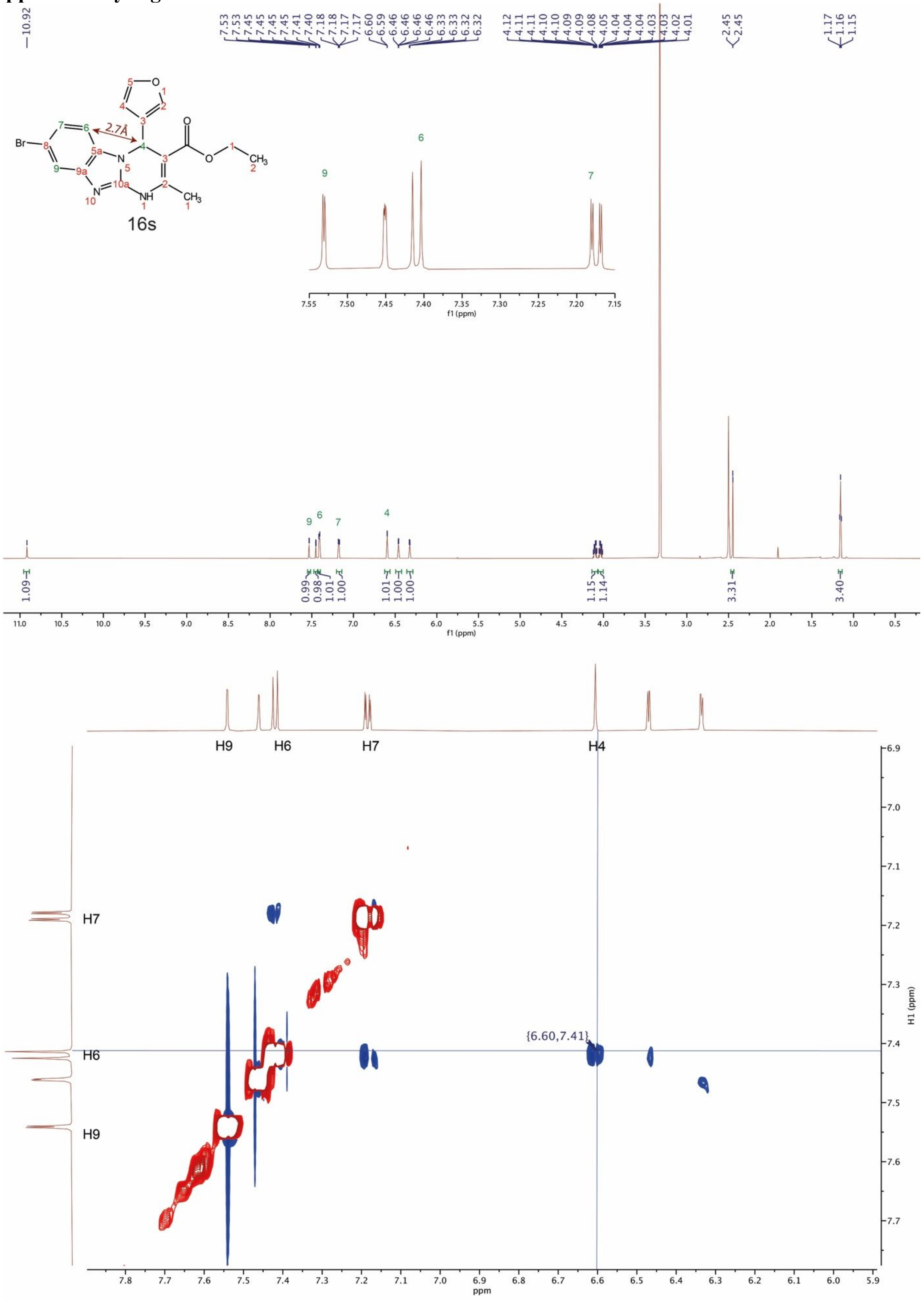

Supplementary Figure S1A. ^{1}H NMR and 2D-ROESY of **16s**.

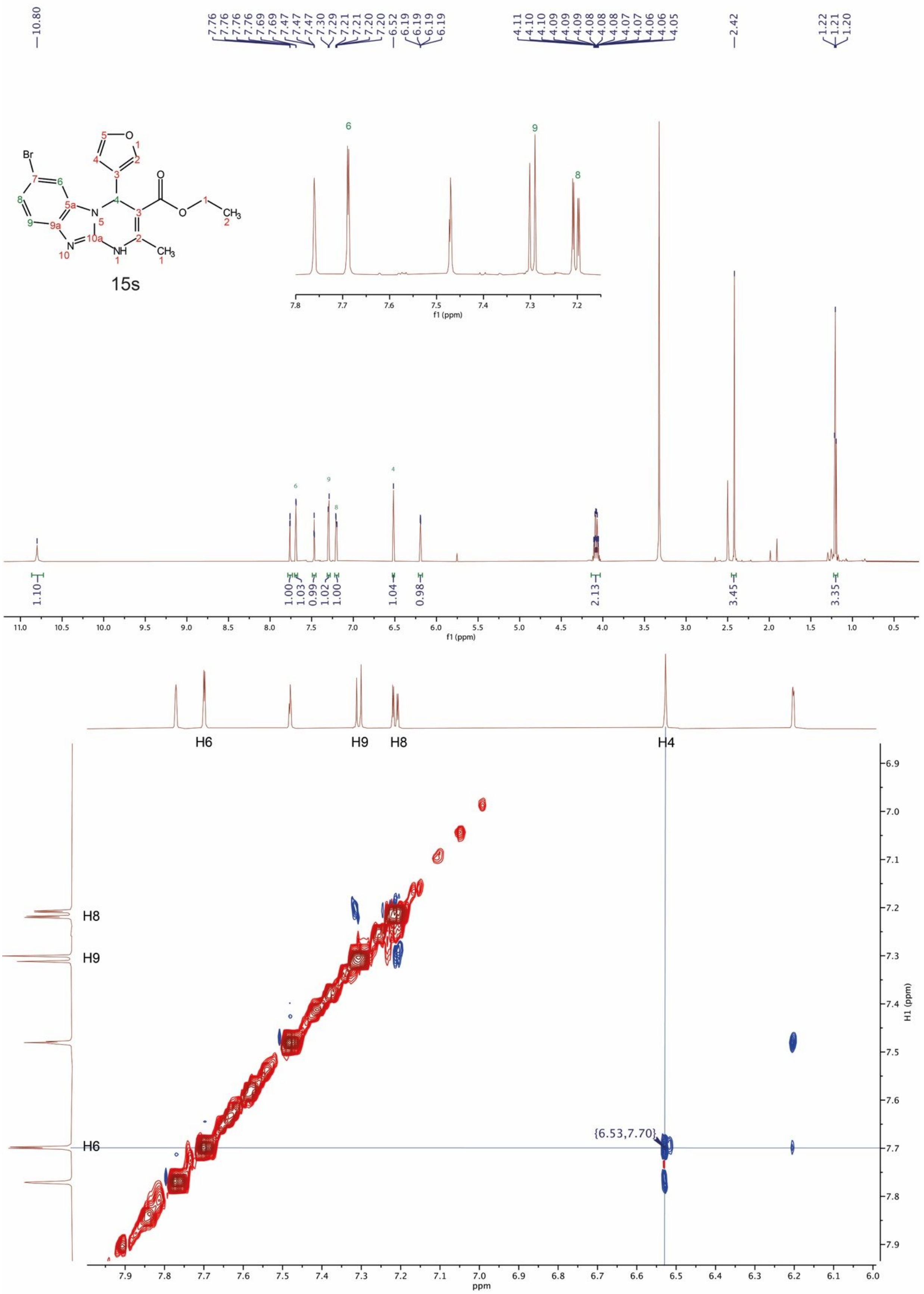

Supplementary Figure S1B. ^{1}H NMR and 2D-ROESY of **15s**.

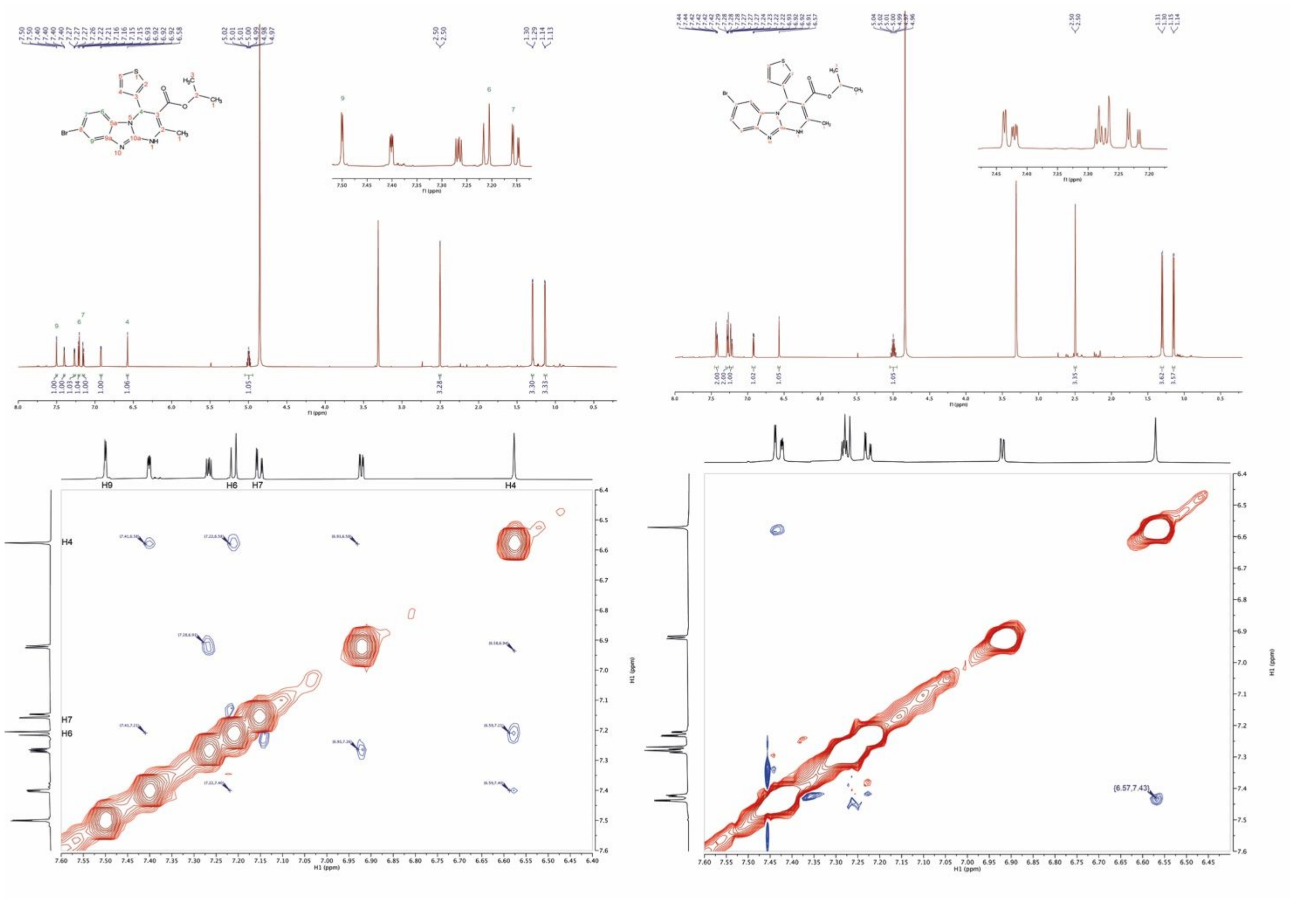

Supplementary Figure S2. ^{1}H NMR (top) and 2D-NOESY (down) of **16x** (left) and **15x** (right)

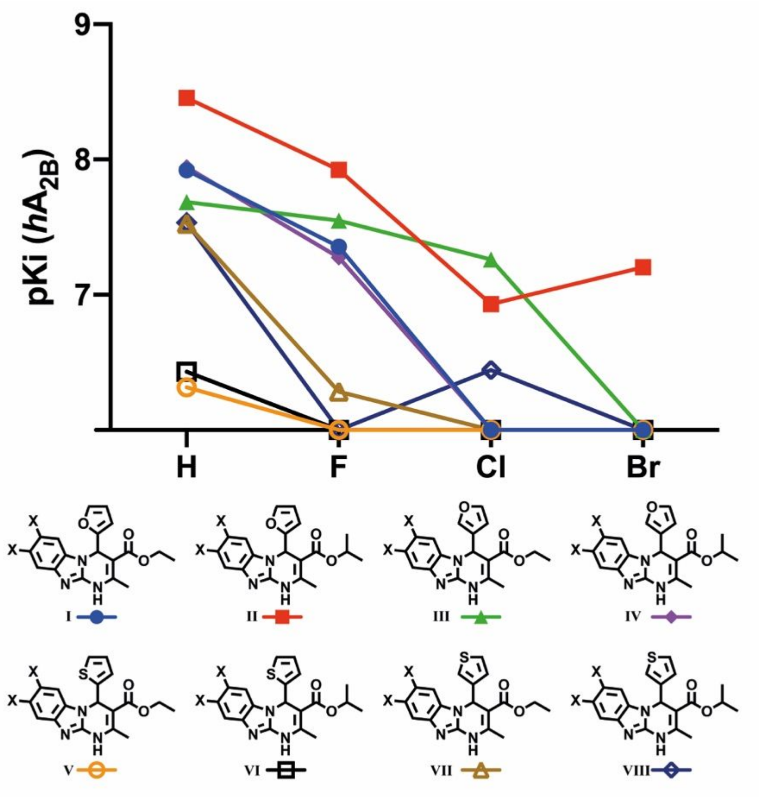

Supplementary Figure S3. pK_i (hA$_{2B}$AR) versus halogen in 7,8-halogenated derivatives (Series **I**). Filled and unfilled shapes represent, respectively, ligands containing furyl and thienyl group at position 4.

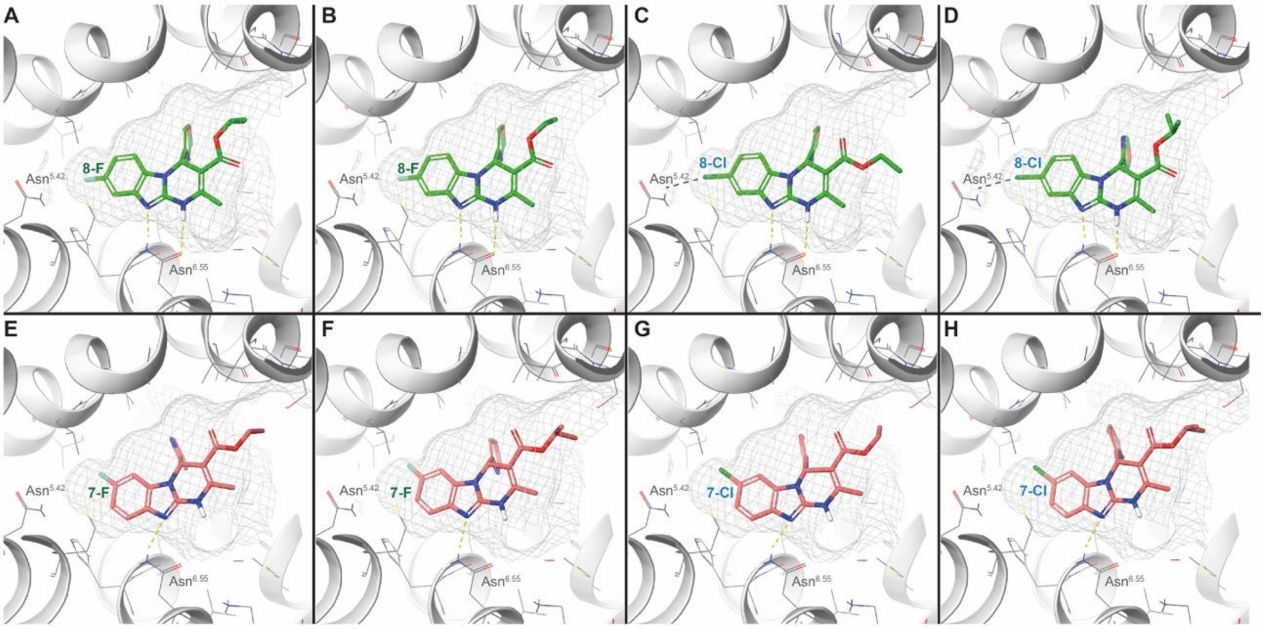

Supplementary Figure S4. Docking poses for Series **IV**. **A) 18a**, **B) 18b**, **C) 18c**, **D) 18d**, **E) 17a**, **F) 17b**, **G) 17c** and **H) 17d**. Grey mesh represent A_{2B}AR binding site surface. Note the loss of a hydrogen bond with $Asn^{6.55}$ in 7-halo derivatives (F-H), and the formation of a halogen bond with $Asn^{5.42}$on 8-chlorine compounds (C-D).

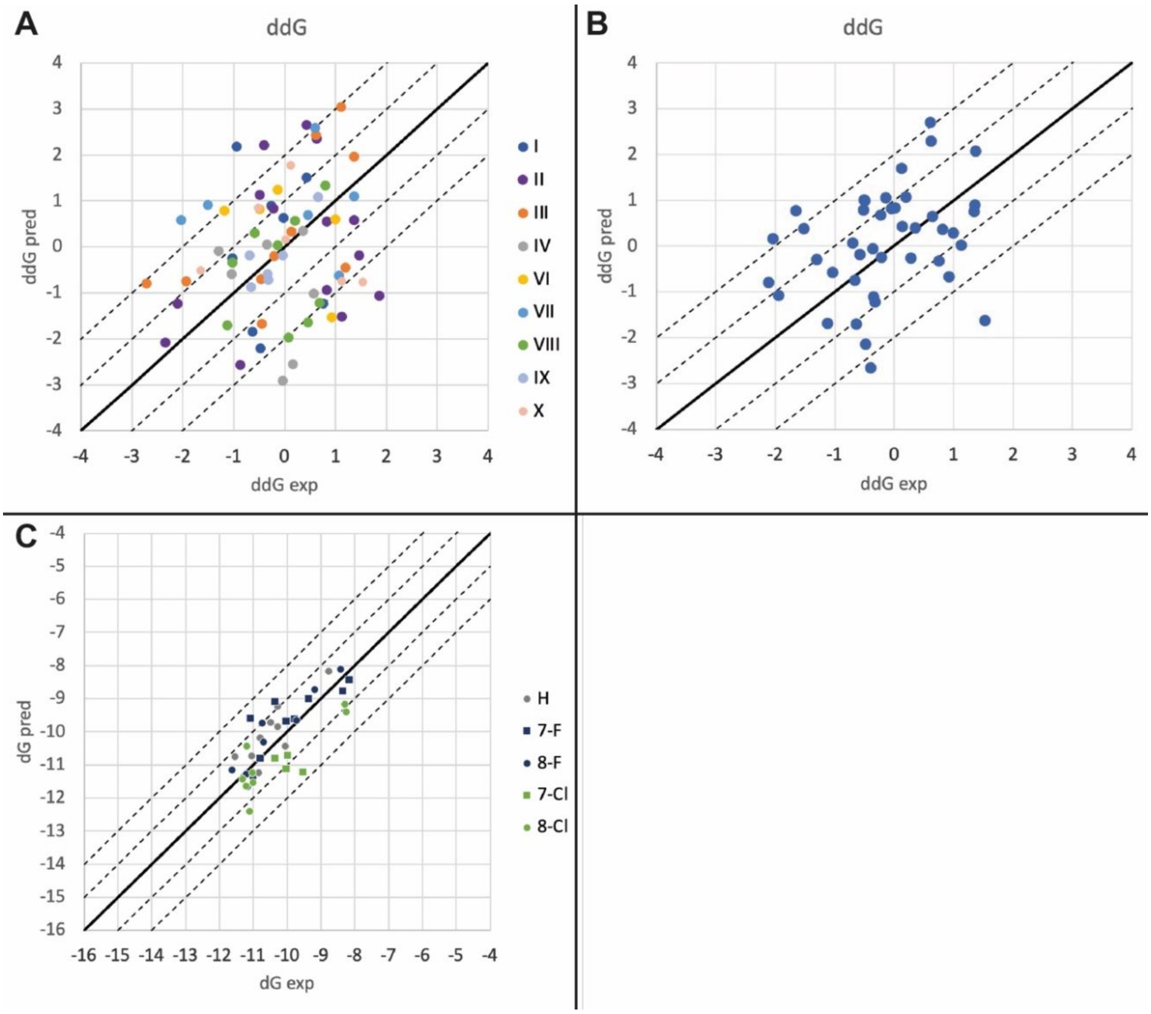

Supplementary Figure S5. A) ΔΔG FEP correlation between all compounds showing K_i in pharmacology experimental assays, grouped by scaffold I-X. B) ΔΔG FEP correlation between all compounds showing K_i in pharmacology experimental assays, removing brominated and dihalogenated compounds. C) ΔG FEP correlation between all compounds showing K_i in pharmacology experimental assays, removing brominated and dihalogenated compounds, grouped by position and halogen.

Supplementary Tables

Supplementary Table S1: Relative binding free energies ($\Delta\Delta G_{bind}$) obtained from FEP simulations between diasteroisomer pairs. Data is shown as average ± s.e.m. of 10 independent MD simulations in each case. Values correspond to the bar plot in Supplementary Figure S2.

		$\Delta\Delta G_{bind}$ ± s.e.m (kcal/mol)	
**Eutomer**	**Distomer**	**Eutomer to Distomer**	**Distomer to Eutomer**
(*S*)-II	(*R*)-II	6.36±0.47	-3.42±0.72
(*S*)-16b	(*R*)-16b	3.89±0.53	-2.39±0.67
(*S*)-16j	(*R*)-16j	3.51±0.63	-3.27±0.76
(*R*)-16l	(*S*)-16l	1.02±0.70	-0.7±0.49
(*S*)-18c	(*R*)-18c	0.72±0.44	-1.23±0.50

Supplementary Table S2. ΔΔG FEP data for Supplementary Figure S3B.

**Ligand 1**	**Ligand 2**	**Exp. ddG**	**Pred. ddG**	**SEM**
15a	I	-1.021	-0.578	0.505
15b	15j	0.764	-0.325	0.485
15c	15k	-0.627	-1.716	0.183
15d	IV	-0.466	-2.148	0.375
15g	VII	-2.1	-0.8	0.16
15h	VIII	-1.93	-1.08	0.208
15j	16j	-1.288	-0.306	0.485
15j	II	-1.505	0.372	0.439
15k	16k	-1.114	-1.691	0.135
15k	III	-0.492	1.005	0.183
16a	15a	1.377	2.062	0.505
16a	16i	0.144	0.418	0.505
16b	15b	0.821	0.355	0.485
16b	16j	0.296	-0.275	0.485
16c	15c	1.36	0.744	0.183
16c	16k	-0.38	-2.664	0.183
16d	15d	0.633	2.292	0.375
16d	16l	-0.203	-0.252	0.375
16f	VI	-0.348	-0.06	0.156
16g	15g	1.01	0.28	0.16
16g	16o	0.937	-0.68	0.16
16h	15h	1.37	0.89	0.208
16h	VIII	-0.56	-0.19	0.208
16i	I	0.211	1.068	0.505
16j	II	-0.216	0.678	0.439
16k	III	0.622	2.695	0.183
16l	IV	0.37	0.397	0.375
16n	16f	-0.133	1.05	0.156
16n	VI	-0.482	0.99	0.156
16o	VII	-2.027	0.16	0.16
17a	17c	-0.33	-1.12	0.665
17b	17d	1.542	-1.634	0.322
17c	18c	-0.646	-0.75	0.665
17c	IX	-0.681	0.06	0.434
17d	18d	-1.644	0.771	0.548
17d	X	-0.51	0.785	0.548
18a	17a	0.659	0.64	0.665
18a	18c	-0.317	-1.23	0.665
18b	17b	0.13	1.696	0.322
18b	18d	0.028	0.834	0.322
18c	IX	-0.035	0.81	0.434
18d	X	1.134	0.015	0.548

Supplementary Table S3. ΔG FEP data for Supplementary Figure S3D.

Ligand	Exp. dG	Pred. dG	SEM
15a	-9.7766742	-9.6127997	0.505
15b	-10.789619	-10.801816	0.485
15c	-9.3605691	-9.0136518	0.183
15d	-10.364094	-9.1013982	0.375
15g	-8.1624459	-8.4482153	0.16
15h	-8.3399159	-8.7834033	0.208
17a	-10.020721	-9.6779061	0.665
17b	-11.069865	-9.593038	0.322
16a	-11.153402	-11.6748	0.71417785
**16b**	**-11.61048**	**-11.156816**	**0**
**16c**	**-10.720956**	**-9.7576518**	**0**
16d	-10.99667	-11.393398	0.53033009
**16f**	**-8.4189833**	**-8.1207072**	**0**
16g	-9.1723599	-8.7282153	0.22627417
**16h**	**-9.7102747**	**-9.6734033**	**0**
**18a**	**-10.67924**	**-10.317906**	**0**
18b	-11.20005	-11.289038	0.45537677
15j	-10.026044	-11.126816	0.68589358
15k	-9.9872445	-10.729652	0.25880108
17c	-10.350761	-10.797906	0.94045202
**17d**	**-9.5275918**	**-11.227038**	**0**
16i	-11.009195	-11.2588	0.505
16j	-11.314252	-11.431816	0.485
16k	-11.101058	-12.421652	0.183
16l	-11.20005	-11.646398	0.375
16n	-8.28581	-9.1707072	0.156
16o	-8.2354565	-9.4082153	0.16
18c	-10.99667	-11.547906	0.665
18d	-11.171622	-10.456038	0.548
**I**	**-10.797928**	**-10.1908**	**0**
II	-11.530685	-10.753816	0.65336667
III	-10.479431	-9.7266518	0.25880108
**IV**	**-10.829779**	**-11.249398**	**0**
IX	-11.032137	-10.737906	0.77711035
VI	-8.7673283	-8.1807072	0.156
**VII**	**-10.262599**	**-9.2482153**	**0**
VIII	-10.270019	-9.8634033	0.208
X	-10.038061	-10.442038	0.548

Project Name Rubén Prieto
Reported by User: Breeze user (Breeze)

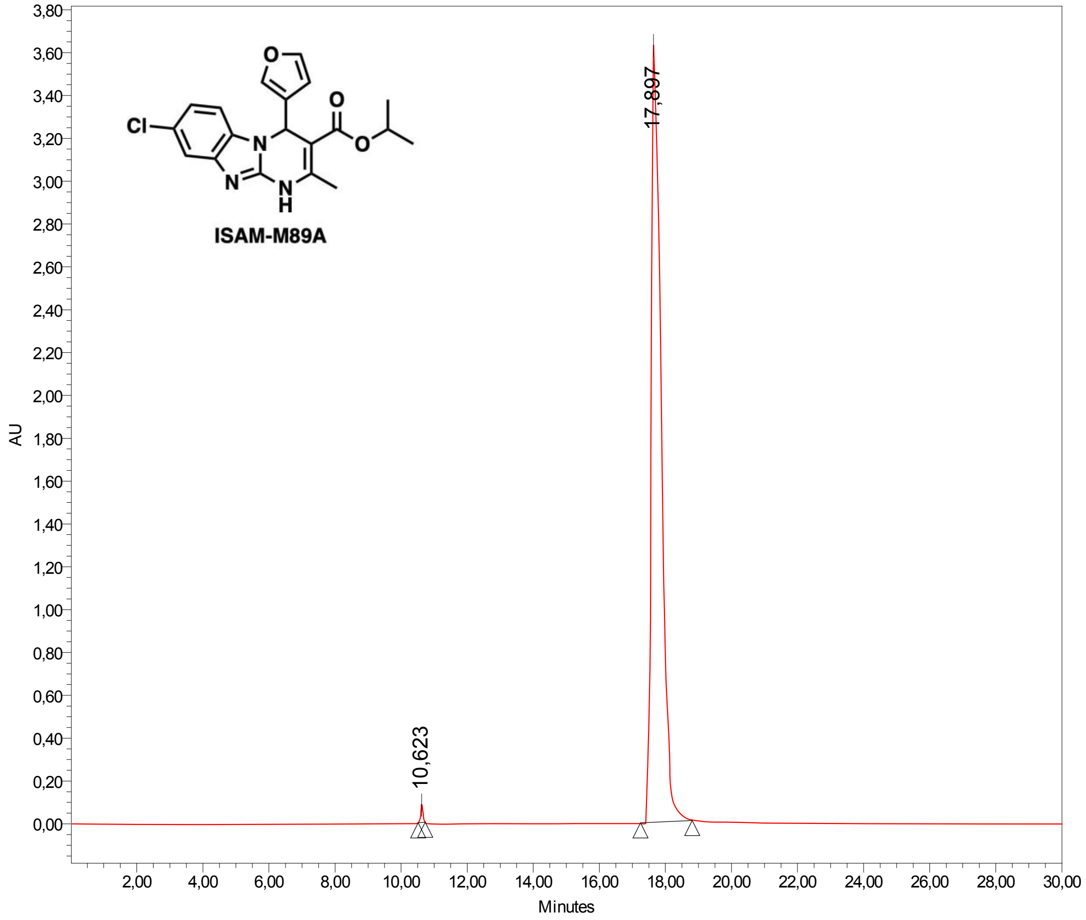

Sample Name: sy1mg-89a; Date Acquired: 23/05/2022 15:09:20 CET; Vial: 1; Injection: 1

Peak Summary with Statistics
Peak Name:

	Sample Name	Vial	Inj.	RT (min)	Area (µV*sec)	% Area	Height (µV)
1	sy1mg-89a	1	1	17,897	79616581	99,46	3740874
2	sy1mg-89a	1	1	10,623	431890	0,54	84429
Mean				14,260	40024236		1912652,54
Std. Dev.				5,14	55992032,02		2585497,20
% RSD				36,07	139,89		135,18

Acquired By	Breeze
Injection	1
Date Acquired	23/05/2022 15:09:20 CET
Run Time	30,00(Minutes)
Acq Method Set	5%DCMiPAGradF1 set
Injection Volume	100,00(uL)
Injection Id	2983
Instrument Method Name	5%DCMiPAGradF1
Superseded	No

Project Name Rubén Prieto
Reported by User: Breeze user (Breeze)

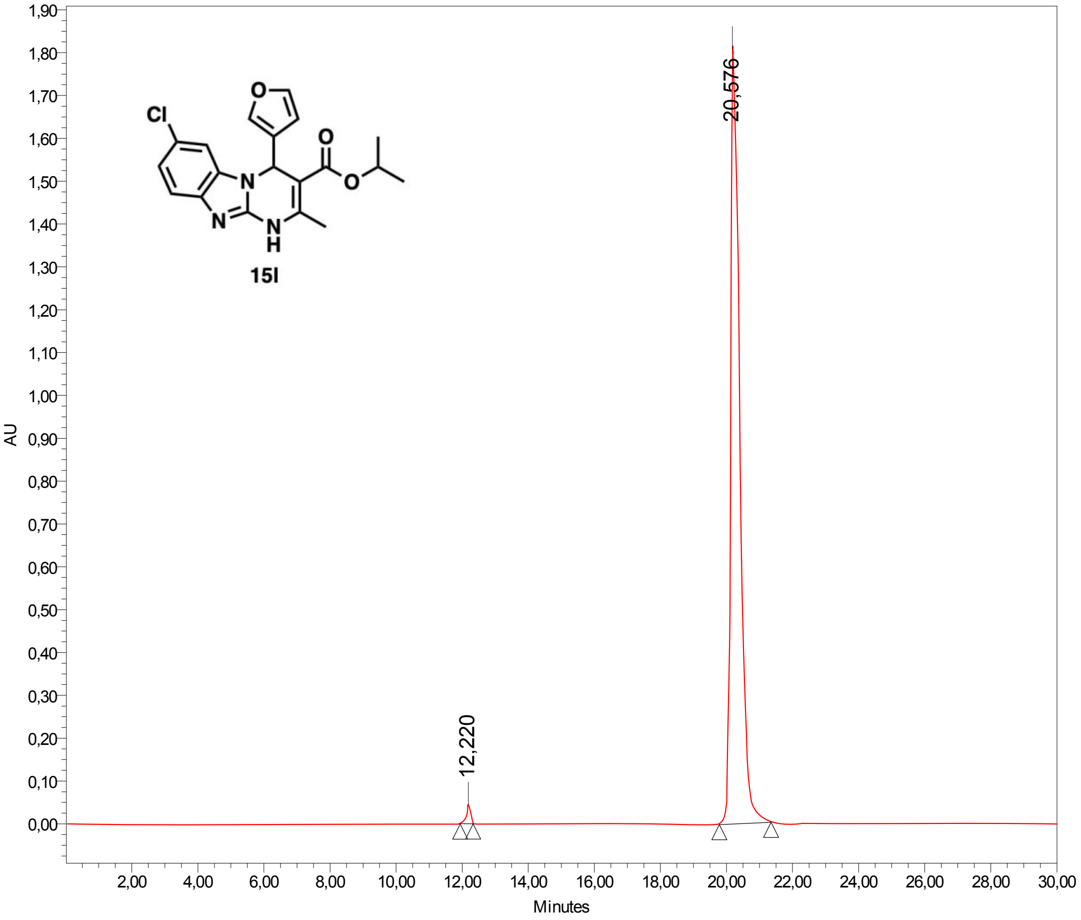

Sample Name: sy1mg-89b; Date Acquired: 23/05/2022 16:42:10 CET; Vial: 1; Injection: 1

Peak Summary with Statistics
Peak Name:

	Sample Name	Vial	Inj.	RT (min)	Area (µV*sec)	% Area	Height (µV)
1	sy1mg-89b	1	1	20,576	81665453	98,02	1799283
2	sy1mg-89b	1	1	12,220	1649639	1,98	33425
Mean				16,398	41657546		916354,00
Std. Dev.				5,90	56579725,07		1248650,17
% RSD				36,03	135.82		136,26

Acquired By	Breeze
Injection	1
Date Acquired	23/05/2022 16:42:10 CET
Run Time	30,00(Minutes)
Acq Method Set	5%DCMiPAGradF1 set
Injection Volume	100,00(uL)
Injection Id	2985
Instrument Method Name	5%DCMiPAGradF1
Superseded	No

Project Name Rubén Prieto
Reported by User: Breeze user (Breeze)

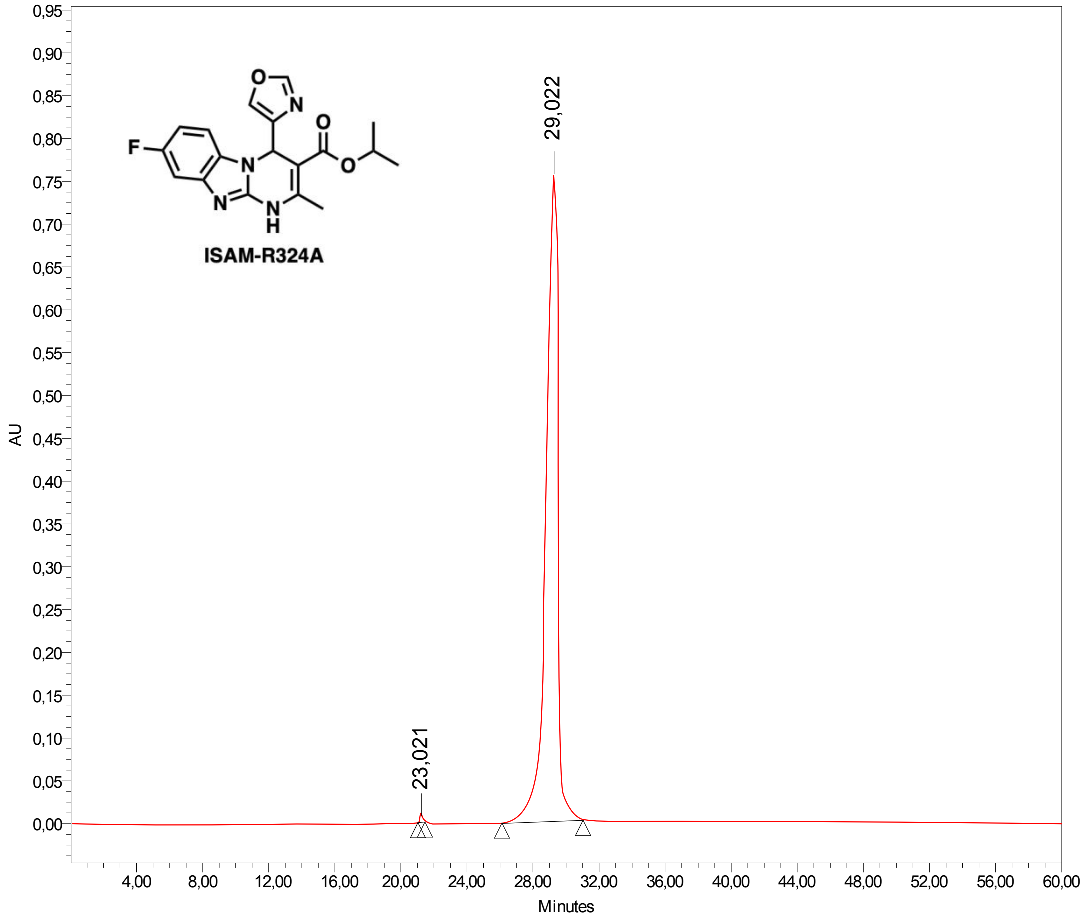

Sample Name: sy1rpd-324a; Date Acquired: 23/05/2022 18:01:22 CET; Vial: 1; Injection: 1

Peak Summary with Statistics
Peak Name:

	Sample Name	Vial	Inj.	RT (min)	Area (µV*sec)	% Area	Height (µV)
1	sy1rpd-324a	1	1	29,022	48814785	99,75	899641,5
2	sy1rpd-324a	1	1	23,021	122343	0,25	16612
Mean				26,02	24468564		458127,16
Std. Dev.				4,24	34430755,83		624395,56
% RSD				16,30	140,71		136,29

Acquired By	Breeze
Injection	1
Date Acquired	23/05/2022 18:01:22 CET
Run Time	60,00(Minutes)
Acq Method Set	5%DCMiPAGradF1 set
Injection Volume	100,00(uL)
Injection Id	2987
Instrument Method Name	5%DCMiPAGradF1
Superseded	No

Project Name Rubén Prieto
Reported by User: Breeze user (Breeze)

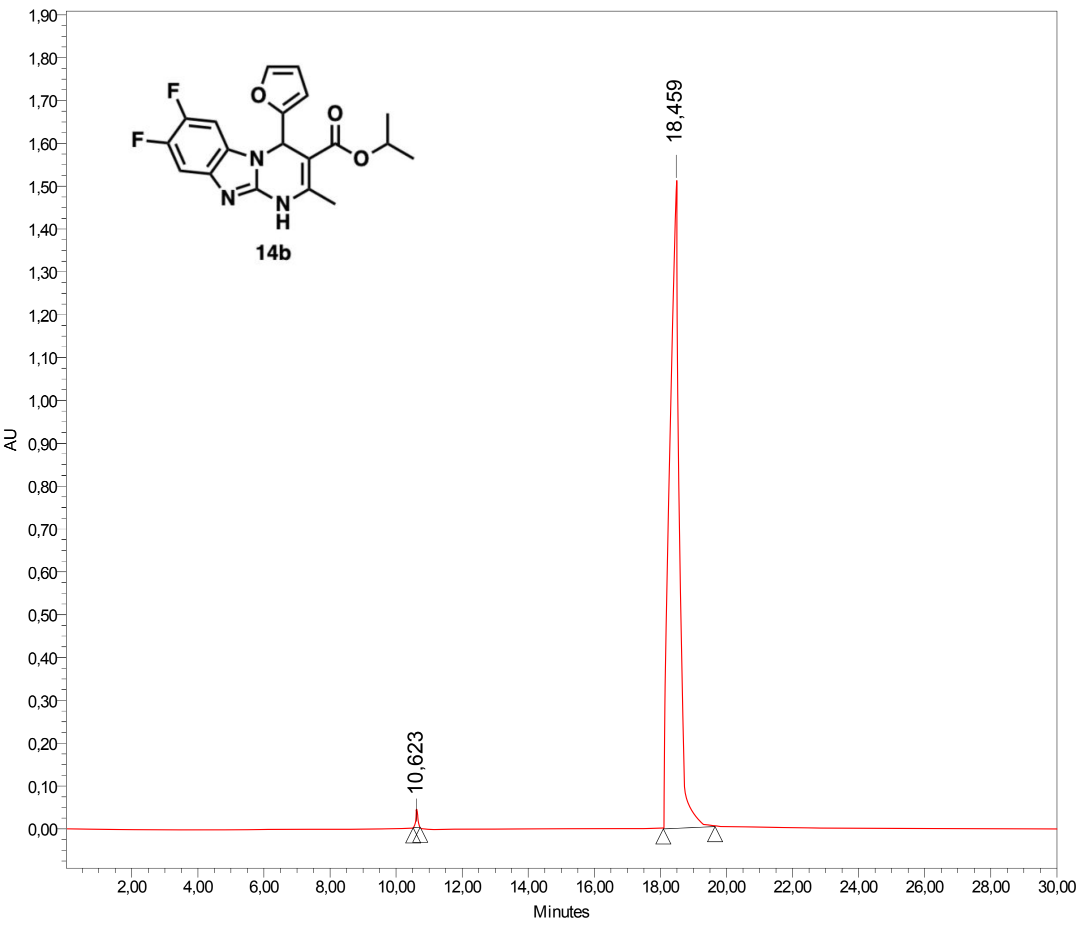

Sample Name: sy1r-440; Date Acquired: 23/05/2022 15:45:20 CET; Vial: 1; Injection: 1

Peak Summary with Statistics
Peak Name:

	Sample Name	Vial	Inj.	RT (min)	Area (µV*sec)	% Area	Height (µV)
1	sy1r-440	1	1	18,459	97874255	99,55	1925278
2	sy1r-440	1	1	10,623	444553	0,45	43452
Mean				14,54	49159404		984365,22
Std. Dev.				5,54	68893203,21		1330651,91
% RSD				38,10	140,14		135,18

Acquired By	Breeze
Injection	1
Date Acquired	23/05/2022 15:45:20 CET
Run Time	30,00(Minutes)
Acq Method Set	5%DCMiPAGradF1 set
Injection Volume	100,00(uL)
Injection Id	2984
Instrument Method Name	5%DCMiPAGradF1
Superseded	No
